# Understanding traffic capacity of urban networks

**DOI:** 10.1038/s41598-019-51539-5

**Published:** 2019-11-08

**Authors:** Allister Loder, Lukas Ambühl, Monica Menendez, Kay W. Axhausen

**Affiliations:** 1 Institute for Transport Planning and Systems, ETH Zurich, Switzerland; 2 Division of Engineering, NYU Abu Dhabi, UAE; 3 Tandon School of Engineering, NYU, USA

**Keywords:** Civil engineering, Statistics, Scientific data

## Abstract

Traffic in an urban network becomes congested once there is a critical number of vehicles in the network. To improve traffic operations, develop new congestion mitigation strategies, and reduce negative traffic externalities, understanding the basic laws governing the network’s critical number of vehicles and the network’s traffic capacity is necessary. However, until now, a holistic understanding of this critical point and an empirical quantification of its driving factors has been missing. Here we show with billions of vehicle observations from more than 40 cities, how road and bus network topology explains around 90% of the empirically observed critical point variation, making it therefore predictable. Importantly, we find a sublinear relationship between network size and critical accumulation emphasizing decreasing marginal returns of infrastructure investment. As transportation networks are the lifeline of our cities, our findings have profound implications on how to build and operate our cities more efficiently.

## Introduction

Fighting congestion is difficult as human travel patterns are repetitive^1,2^ and added capacities are rapidly consumed by induced demand and population growth^3^. Physically, traffic is a many-particle system^4^, where congestion is defined as the state when increasing the number of vehicles decreases travel production. In contrast, during free-flow states, increasing the number of vehicles increases travel production. The system’s *critical point* is then located at the boundary between the network’s free-flow and congested states. At this point, the maximum in travel production, the traffic capacity of urban networks, is reached. Consequently, the critical point marks both, the boundary between free-flow and congestion, and the boundary of possible travel production.

The urban road transportation system can be analyzed at the link and network level. The link level is well understood and design procedures are standardized^5^; but the understanding of entire networks is by far not comparable. Differences in critical points of urban networks have been observed since the 1960s^6–8^. However, until now, a holistic understanding of the critical points of entire networks was missing^9^, as well as an empirical quantification of its driving factors. Fortunately, the recently formulated Macroscopic Fundamental Diagram (MFD) provides new ways to systematically analyze urban traffic at the network level^10–12^, it is consistent with the physics of traffic, and allows to determine the boundary of traffic states of networks and thus the traffic capacity of urban networks.

The MFD relates the vehicle accumulation in an urban network *n* (vehicles) to the travel production *P*(*n*) (vehicle-km h^−1^) with a smooth and reproducible curve exhibiting a maximum P*, the overall traffic capacity of urban networks, at the critical accumulation of vehicles n^*^. Consequently, the critical point is (n^*^, P^*^). Average network speed *v* follows from the fundamental traffic equation *v *= *P*/*n*; and the speed at which congestion starts is the critical speed v^*^ = P^*^/n^*^. In the following, we express *P*(*n*) in units (vehicle-km h^−1^ km^−2^) and *n* in units (vehicles km^−2^) to allow a better comparison across networks of different sizes. If *n *> n^*^, the network is congested and production decreases, and if *n *< n^*^, the network is in free-flow and thus uncongested. To maximize travel production, consequently, an ideal traffic control should act at the critical point^10^, i.e. *n *≈ n^*^.

**Figure 1:**
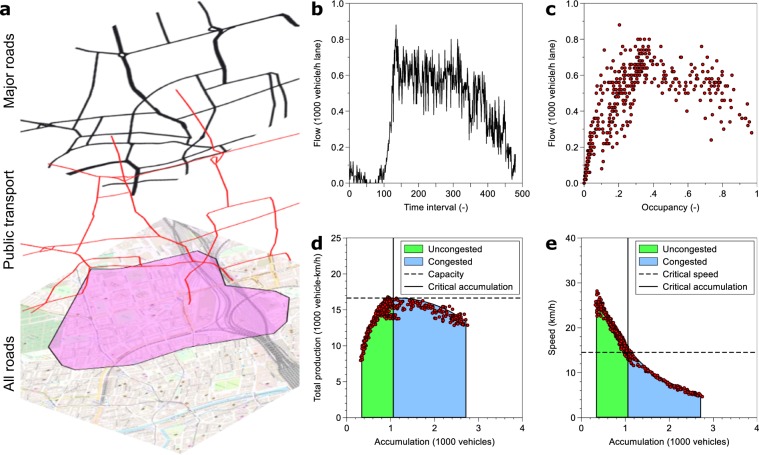
Traffic in urban road networks. (**a**) Shows the layers of urban transportation networks. The city with all streets forms the basis, where a major road network takes over the connecting functions and then the public transport system. Background map is courtesy of OpenStreetMap. (**b**) Time series of a single detector on a street in Zurich, and (**c**) the corresponding scatter plot of detector occupancy versus flow. When aggregating al measurements in the purple area in (**a**), smooth curves in the flow-density domain (**d**), and in the speed density domain (**e**) emerge.

MFDs exist and are well-defined in roughly homogeneously congested networks^10^. Thus, they are not estimated for entire cities, but in smaller *regional networks* as indicated in Fig. 1a. They tend to cover areas less than 10km^2^ in size and can be defined using network partitioning algorithms^13^. In theory, the MFD’s shape results from road and bus network topology, route and mode choice, and traffic signal control^11,14,15^, but it is empirically difficult to directly measure *n* and *P*(*n*) or analytically derive *P*(*n*). Fortunately^12^, showed how the MFD can be approximated with existing traffic monitoring systems, located on main urban roads in many cities as shown in Fig. 1a. Aggregating somehow chaotic single detector measurements - as seen in Fig. 1b,c - inside a regional network, results in the smooth MFDs curve between production and accumulation as seen in Fig. 1d, and between speed and accumulation in Fig. 1e. It can be clearly seen that the critical point defines the border between uncongested and congested traffic states as well as the maximum in the boundary of physically possible traffic states. However, it is not yet known which factors affect the values associated with the critical point.

To address this gap, in this paper, for the first time, we analyze critical points, with data from 41 cities worldwide, including 107 regional networks (see list of cities in Table 1 and networks and MFDs in Fig. 1 to 107 in the Supplementary Information). We use a newly assembled, rich empirical data set of billions of vehicle observations from stationary traffic detectors to estimate critical points. We pair this data with networks from OpenStreetMap to explain how network topology drives the critical point.

**Table 1:** The critical point model. All effects are statistically significant at least at the 5% level of statistical significance.

Covariates	Unit	Dependent variables			
		Critical accumulation n^*^ (vehicle km^−2^)		Capacity P^*^ (vehicle-km h^−1^ km^−2^)	
		*β*	*t-value*	*β*	*t-value*
Constant *n* ^*^ *R* *R* × *I* *b*_*c*_ *B*	(vehicle km^−2^) (lane-km km^−2^) (lane-km km^−2^ × LSA km^−1^) (−) (bus-km h^−1^ km^−2^)	−292.73 24.40 −5.34 −140.67 0.307	(−3.46) (15.31) (−2.00) (−4.02) (2.12)	1282.29 18.63 −6.64	(1.56) (18.87) (−5.19)
N Adj. R^2^		107 0.88		107 0.91	
	**Elasticity**		**Hypothesis test: H**_**0**_ **:** ***ε*** ** = 1**		
	***ε***	**95% CI**			
∂ log P^*^/∂ log n^*^ ∂ log n^*^/∂ log *R*	0.968 0.839	[0.828;1.107] [0.727;0.950]	F(1,40) = 0.20 F(1,40) = 215.60	Prob > F = 0.655 Prob > F = 0.000	not reject reject

The probability that n^*^ is exogenous is p = 0.000. We cluster standard errors at the city level and weigh observations by a measure of certainty in the critical point estimation. As we cluster standard errors, the test for endogeneity is based on Wooldridge’s robust score test (the equivalent in case of unadjusted standard errors would be the Durbin-Wu-Hausman test). In the Supplementary information, we provide in Tables 2–4 robustness analyses of the model without weights, in a log transformation, and critical density (not accumulation) formulation. The lower part of the table shows elasticity estimates (Delta-method) with the 95% confidence intervals between critical accumulation and capacity as well as road network density and accumulation. For each elasticity, we test the hypothesis of being different from one.

The estimation of critical points requires first the identification of suitable regional networks where the MFD, critical points as well as the network topology are estimated and analyzed. We define regional networks heuristically, as existing partitioning algorithms^13,16^ cannot be applied to our data and limited detector coverage usually prevents a systematic and automatic zoning at large scale across all cities. Second, we estimate MFDs using a re-sampling methodology^17^ to account for heterogeneity in the network because we want to clearly identify the boundary of traffic states as independent as possible of demand and traffic irregularities. Third, we fit an MFD function^18^ in the uppermost 20-quantile (quantile regression approach) of the estimated MFDs to functionally describe the boundary of traffic states. The critical point is then the maximum of the estimated function. This allows a more robust estimation of the critical point because this procedure borrows information from all observations in the MFD.

## Results

**Figure 2:**
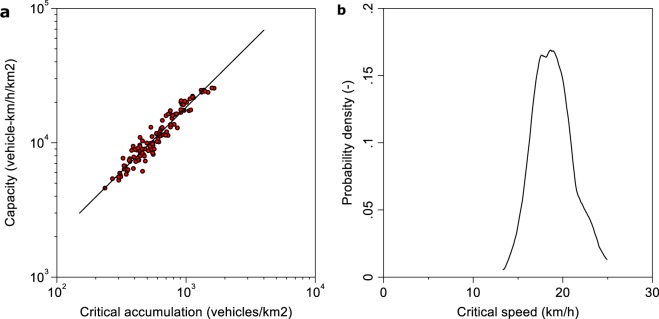
Comparison of urban traffic capacity across 107 urban networks. (**a**) Positive relationship of network capacity against critical accumulation (*R*^2^ = 0.92). (**b**) Kernel density estimate of critical speeds of all networks emphasizes that congestion starts at different speeds in different networks (mean 18.8 km/h).

Figure 2 compares the critical points of all 107 networks with sample statistics $\overline{{P}^{\ast }}=12236\pm 5513$ vehicle-km h^−1^ km^−2^ (mean ± standard deviation) and $\overline{{n}^{\ast }}=653\pm 308$ vehicles km^−2^ (mean ± standard deviation). Figure 2a shows the intuitive positive relationship between critical accumulation and network capacity with *R*^2 ^= 0.91. *R*^2 ^< 1 implies – ignoring measurement uncertainties - that other factors create variation in critical points as well. This variation is also emphasized by the kernel density estimate of the critical speed in Fig. 2b, revealing a substantial variation of critical points across regions. This implies that in some regions, car drivers can experience low speeds, but against intuition, the network is not macroscopically congested. The distribution of critical accumulation in our sample is consistent with the traffic physics literature^19^ that predicts that critical accumulation does not exceed one third of the jam accumulation.

To quantify the critical point relationship in Fig. 2a and the influence of bus and road network topology on the critical point, we use a two-stage least squares regression analysis because the influence of critical accumulation on capacity is endogenous and requires instrumental variables. We focus on exogenous variables linked to the full network properties of bus and road network topology that are not only macroscopic, but also measurable from OpenStreetMap, common to all cities, and clearly interpretable. We do not consider city-specific factors, e.g. bicycle policies, although they undoubtedly also influence the critical point. On a broader level, the exogenous variables should describe conflicts between vehicles that cause delays. For example, delays appear when two vehicles want to be at the same time at the same place (i.e. at intersections), or when cars must follow buses or cyclists. All these conflicts influence the total travel time of drivers or even the travelled distance of vehicles, both of which affect the location of the critical point. Accordingly, we define four variables. First, road network density *R* (lane-km km^−2^) with sample statistics $\bar{R}=23.46\pm 10.36$ lane-km km^−2^ (mean ± standard deviation). Second, network redundancy, measured in the network average betweenness centrality *b*_*c*_ (−) with sample statistics $\overline{{b}_{c}}=0.084\pm 0.039$ (mean ± standard deviation). Third, the average distance between or spacing of signalized intersections (LSA) in the network *I* (LSA network-km^−1^) with sample statistics $\bar{I}=0.385\pm 0.188$ LSA network-km^−1^ (mean ± standard deviation). Fourth, the bus production density *B* (bus-km h^−1^ km^−2^) that combines the density of the bus network per unit area with the average headway with sample statistics $\bar{B}=147\pm 85.5$ bus-km h^−1^ km^−2^ (mean ± standard deviation). We expect that *R*, *b*_*c*_ and *I* affect the critical accumulation (via total travel time) and *B* affects capacity (via total travelled distance). Consequently, the first three variables correspond to the required instruments for the critical accumulation. In particular, we expect that n^*^ increases with *R* as more road space per unit area generally allows more vehicles to circulate, but denser networks provide more opportunities for conflicts (at intersections, through lane changes, etc.), resulting in a sublinear scaling^8,20,21^ between *R* and n^*^. In low redundancy networks with few alternative routes, the concentration of vehicles on these routes reduces the critical accumulation compared to networks with many alternative routes^22–24^, i.e. n^*^ decreases with *b*_*c*_. In a network of density *R*, we expect that a denser intersection spacing increases travel times due to more waiting compared to a network of the same density *R* with a larger intersection spacing^5,6,15,25^, consequently we expect that the interaction of *I* and *R* has a negative effect on P^*^. Last, buses are usually larger than cars and can behave as either stationary bottlenecks at bus stops, or as moving bottlenecks when driving, consequently, P^*^ should decrease with *B*^26–30^.

Table 1 summarizes the model estimates. Tables 2–4 in the Supplementary information provide alternative model specification to check the robustness of the critical point model that are formulated without observation weights, in log-specification and in a critical density (not accumulation). While the first two robustness model specifications test the influence of observation weights and functional form, we rule out with the latter the possibility of a bias caused by scaling density to accumulation. We have to reject the hypothesis that critical accumulation is exogenous, consequently, the two stage least squares is required. We find that we can significantly explain around 90% of the observed critical point variation with just four exogenous variables describing bus and road network topology. We cannot explain the entire variation because we do not include city-specific factors, nor those that are not clearly measurable. Further, we see that the elasticity of n^*^ is not statistically significantly different from one, meaning that efforts to increase the critical accumulation affect capacity proportionally. Contrary, we find that the elasticity of *R* on the critical accumulation is significantly different from one, with *ε* ≈ 0.8. All robustness models in the Supplementary information confirm this result.

**Figure 3:**
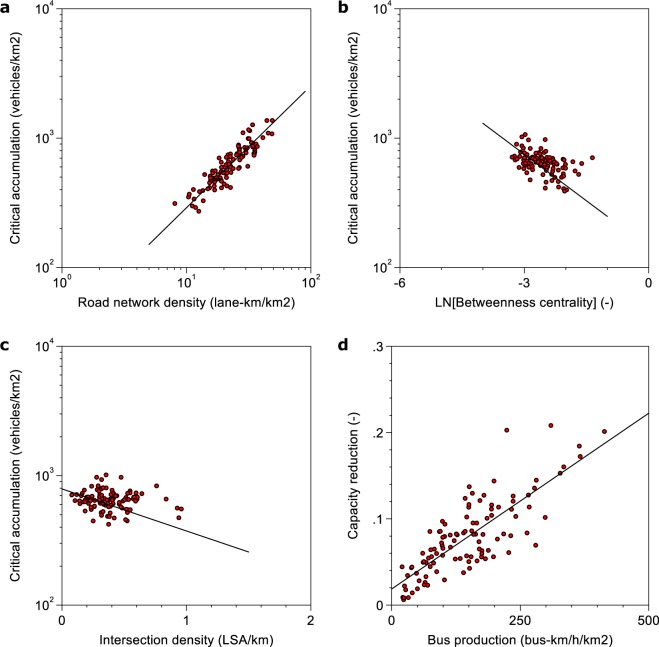
Illustration of the effects in the critical point model. (**a**) Influence of road network density on critical accumulation. (**b**) Influence of betweenness centrality (network redundancy) on critical accumulation. (**c**) Influence of intersection density on critical accumulation. (**d**) Capacity reduction by bus operations in the network. Panels (a–c) are constructed as follows. We first residualize the x- and y-variables on the other control variables of the critical point model as given in Table 1. Secondly, we add back the unconditional mean of the x- and y-variables to the residuals. Panel (d) is constructed by calculating each network’s capacity at zero bus production and then calculating the relative difference to the observed capacity. The solid black line in each panel corresponds to the linear fit of the scatterplots.

**Figure 4:**
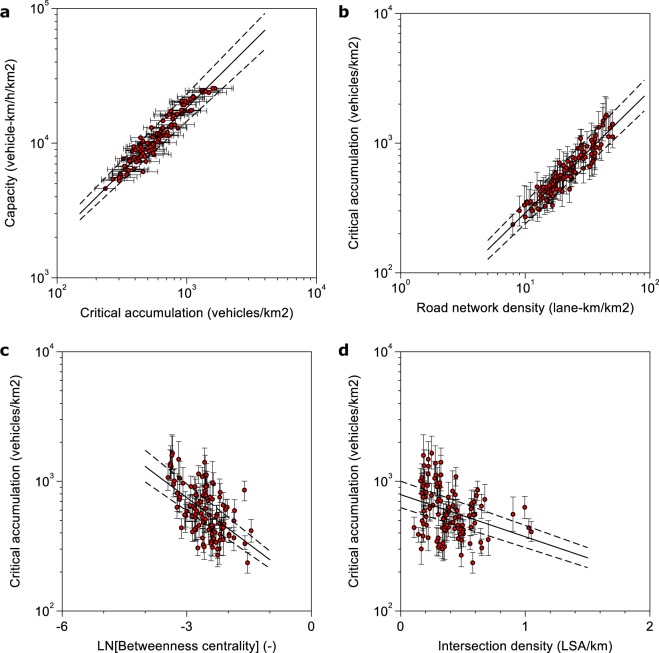
Robustness check for the situation if the critical accumulation becomes an interval. The points in each panel correspond to the estimated maximum of each MFD, while the horizontal or vertical bars describe the interval in accumulation that results from a 5% reduction of the capacity. (**a**) Scatterplot capacity versus accumulation. (**b**) Influence of road network density. (**c**) Influence of betweenness centrality. (**d**) Influence of intersection density.

In Fig. 3, we illustrate the effects underlying the results from Table 1 graphically to show how the variation in the data drives the critical point variation. We conclude that even with the rather small empirical variation in bus and road network topology across cities, there is substantial variation in the critical point. Figure 4 provides a sensitivity analysis of the presented relationships for a likely occurrence of n^*^ becoming an interval (i.e. multiple maxima) instead of a single point^10^. This behavior can occur due to the complexity of urban traffic and for uncertainty in the critical density estimation. This sensitivity analysis shows that the identified relationships are robust against the occurrence of n^*^ becoming an interval.

**Figure 5:**
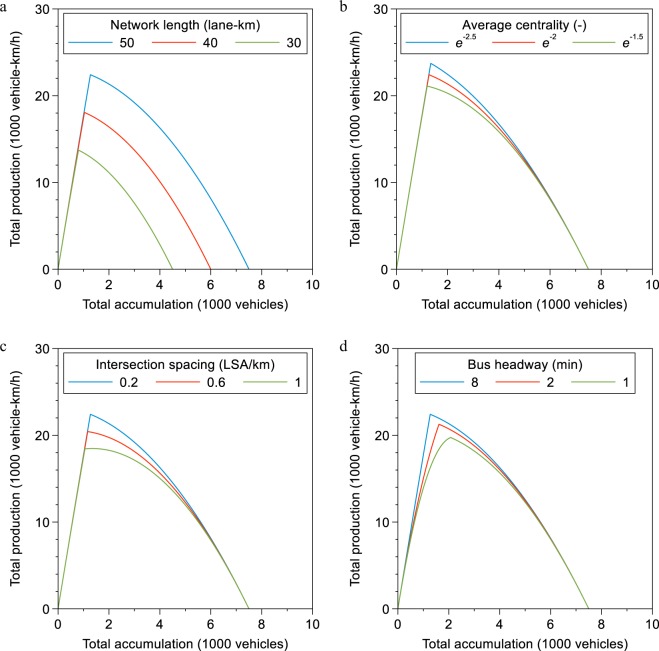
Illustration of the effects in the critical point model. (**a**) Influence of road network density on critical accumulation. (**b**) Influence of betweenness centrality (network redundancy) on critical accumulation. (**c**) Influence of intersection density on critical accumulation. (**d**) Capacity reduction by bus operations in the network. The MFDs are constructed as follows. We use the model estimates from Table 1 to predict the critical point. We use the following values for the variables that remain fixed in each graph: *R* = 50 lane-km km^−2^, *b*_*c*_ = −2, *I* = 0.2 LSA km^−1^, *LBN* = 50 line-network-km km^−2^, *HW* = 8/60 h.

We emphasize in Fig. 5 the effects of changes in bus and road network topology on the critical point and the MFD. In other words, the results in Fig. 5 illustrate macroscopic trade-offs for urban transportation policies. We choose a simple bi-quadratic function^31^ for the MFD to clearly mark the critical point location. In particular, we show in Fig. 5a the influence of road network density, in Fig. 5b the influence of network redundancy, in Fig. 5c the influence of intersection spacing, and in Fig. 5d the influence of bus headways. Clearly, Fig. 5a shows that more roads in the network increase the total production of the network, but the elasticity of road network density from Table 1 points towards the important policy implication of decreasing marginal returns of road network expansion. Figure 5b,c describe the macroscopic trade-off between vehicular and non-vehicular traffic and walkability of a city. More intersections provide more right of way to pedestrians and a concentration of vehicular traffic on few routes could mean less negative car externalities for non-vehicular modes elsewhere in the network. Figure 5d emphasizes a universal trade-off between buses and cars allowing cities to optimize passenger throughput under further assumptions on vehicle occupancy levels and bus services. The results from Table 1 and the illustrations in Fig. 5 imply that cities can now understand how investment measures benefit or harm the capacity of entire networks. Importantly, these measures go beyond just building roads.

## Discussion

In this paper, we empirically analyzed for the first time how network topology affects the critical point of the macroscopic fundamental diagram (MFD) and thus network-wide traffic. We used traffic and network data from 41 cities worldwide with billions of vehicle observations and found that just four variables of road and bus network topology explain around 90% of the empirically observed critical point variation. Importantly, we find a sublinear relationship between network size and critical accumulation emphasizing decreasing marginal returns of infrastructure investment. Consequently, our findings make the critical point predictable and the MFD more reliable for economic and engineering applications as well as policy making.

Our findings have practical implications. First, the possibility to identify a city’s optimal infrastructure may help to attain the full benefits of urban scaling^32^. Second, the estimates provide means for discussing urban space allocation. However, our study also faces limitations. First, our sample is still relatively small and mostly located in Europe, limiting our findings’ (geographic) validity. Second, we cannot consider some topological and operational features (e.g. intersection design, transit priority) as they are either unavailable or not clearly measurable. Last, we do not cover traffic control. However, since control strategies are typically derived from between-vehicle conflicts, which we do consider, we expect to explain many traffic signal effects with our four variables^11,15,33^. Future research can use the collected data to analyze the dynamics of network traffic, in particular to investigate at network level the determinants of when they reach congestion and for how long cities stay congested.

In closing, the variables derived from bus and road networks, significantly explain the critical point and the traffic capacity of urban networks, making it predictable. This has profound implications for transportation investments. Cities can now identify their optimal infrastructure level and the macroscopic effects of investment decisions on the (multimodal) network performance. This new understanding of traffic capacity of urban networks may not solve congestion problems, but it is crucial for the development of new strategies to improve traffic.

## Methods

### Initial dataset

MFDs are estimated from stationary traffic sensors, most of them inductive loop detectors. They report vehicle flow *q*_*i*_(*t*) and the fraction of time the detector is occupied *o*_*i*_(*t*), during an observation interval *t* with a typical duration of 300 seconds. Here, we use detectors located on roads with the following OpenStreetMap attributes: trunk, primary, secondary, and tertiary roads. We filter the data for obvious errors^34^ (e.g. flow but no occupancy measurements), removed obvious outliers^35^ and obvious noise^36^. Figure 1 to 107 in the Supplementary information show for each regional network the unfiltered MFD. While some networks already exhibit a smooth and well-defined MFD, others do not and clearly require a careful data preparation.

### MFD estimation and calibration

We estimate MFDs using the *loops method*^12,37^, and account for the bias caused by the detector location^37–39^. To account for the heterogeneity of link flows and densities, to identify pockets of congestion, and to clearly identify the boundary of traffic states as independent as possible of demand and traffic irregularities we use a re-sampling methodology in the MFD estimation^17^ (300 draws at 80% sample size each). This approach, despite not capturing the temporal aspects of traffic, identifies the boundary of physically possible traffic states as it significantly removes the influence of demand and traffic irregularities.

Loop detectors usually do not reliably report speeds as they require calibration with detector and car length information^34,40,41^, which are unknown in most cities in our sample. Consequently, we employ a two-step calibration routine, where in the first step we reduce the uncertainty at the detector level and in the second step we calibrate the entire MFD to external speed information. The first step values are irrelevant as they are completely re-calibrated in the second step. First, to reduce the noise at the detector level, we rescale occupancy in such a way that *q*/*o* (approximation of speed) for each detector in free flow conditions corresponds to the average speed expected at that location^42^. This does not change the information provided by the detector, but reduces the scatter in the aggregation as rescaled occupancy measurements now have a similar physical interpretation. In the second step, we use Google Directions API to provide reference speed levels. We calibrate the estimated MFD by matching the average speed from the fastest observed hour in the MFD to the average speed in the regional network queried from the API for that same hour (1000 random origin-destination pairs, 1–6 kilometers long, excluding freeways).

The estimated MFD from single detectors results in average flow and density per lane that need to be scaled by the network length^10^ *L* to represent the production versus accumulation MFD. We expect that this scaling is not introducing a bias to the MFD as we measure the traffic data and L on the same road hierarchy level and ensured that our sample only includes cities where detectors are distributed across all considered road hierarchy levels.

### Critical point estimation

We estimate the critical point by fitting a non-linear MFD function^18^ with nonlinear least squares to the upper bound of the re-sampled MFD and calculate the maximum of the fitted function as the critical point. This procedure borrows information from all observations in the MFD, resulting in a more robust estimate. Taking just the maximum value of the measured values would ignore the available information in all the data, and would be sensitive to outliers. To extract the upper bound of the re-sampled MFD that most likely describes the boundary of traffic states, we follow a quantile regression approach^43^: we identify a stable upper bound by extracting the flow’s 95^th^–97.5^th^ percentile at density bins. Figure 1 to 107 in the Supplementary information show the estimated upper bound and the fitted MFD curve.

### Calculation of observation weights

Each observation of a critical point comes with uncertainty due to potential measurement, data filtering, and calibration errors. We calculate for each critical point *i* a weight ${w}_{i}=\frac{\log \,N}{{P}_{95}^{\ast }-{P}_{5}^{\ast }}\exp (-\frac{{s}_{high}-{s}_{low}}{{s}_{mean}})$. The weights increase with the number of observed intervals *N*. This is intuitive as we expect that an MFD and its critical point observed for a longer period are more reliable. For the uncertainty in flow measurements, we assign a higher weight if the travel production distribution at the critical accumulation is more dense (compact) and has less scatter and long tails. We quantify this by the difference between the 95^th^ quantile ${P}_{95}^{\ast }$ and the 5^th^ quantile ${P}_{5}^{\ast }$ of travel production at the critical accumulation n^*^. For the uncertainty in density, we consider the dispersion of the speed calibration scalar distributions using *s*_*mean*_, *s*_*low*_, and *s*_*high*_. *s*_*mean*_ is the calibration scalar obtained for the MFD calibration, while *s*_*low*_ is the 85^th^ percentile of the API speed divided by the 95^th^ percentile of MFD speed, and *s*_*high*_ is the 95^th^ percentile of the API speed divided by the 85^th^ percentile of MFD speed. If the relative dispersion of *s* increases, the weight decreases. We use the exp(*x*) function to obtain unity as a factor for the uncertainty in critical density if no speed calibration is necessary due to the availability of speed measurements. In case the MFD has a travel production plateau, uncertainty in critical density increases and thus decreases the attributed observation weight.

### Derivation of bus and road network topology variables

We use network data from OpenStreetMap queried in autumn 2017. We use a slightly larger area than that for which traffic data is available to reduce the error associated with the omission of important links and to keep a proper level of connectivity in the graph. For the analysis of network features in each region of size *A*, we only consider the elements inside that region.

We consider only roads with a connecting function, and exclude residential and service roads. In the context of OpenStreetMap we focus on tertiary, secondary, primary, and trunk roads. We obtained for each road segment the number of lanes from OpenStreetMap. We validated each network in terms of connectivity and completeness and simplified intersections where applicable, so that a simple four-way intersection was represented by a single node. We marked each node representing an intersection in the network. We updated link attributes if necessary (either changed attributes manually or replaced missing information by the average for the given functional road class). We processed each network further by amalgamating street segments at pseudo nodes, i.e. a node with degree of two (except if the node was an intersection). As a last step, we created a directed network with nodes corresponding to intersections and edges to streets^44^. We then calculate the road network density as *R *= *L*/*A*, where *L* is the total network length in lane-kilometers. The intersection spacing is then obtained as $I=\#LSA/L/\bar{l}$, where #*LSA* is the number of signalized intersections and roundabouts in the network and $\bar{l}$ is the average number of lanes in the network. Last, betweenness centrality, *b*_*c*_, measures the number of shortest paths over a node divided by the total number of possible origin-destination pairs^45,46^. Thus, centrality measures how likely it is for several routes in the network to overlap. For comparing different networks, we limit shortest paths to 6 km and we add a buffer of 1 km to avoid boundary effects within our region, but we consider only the nodes within each analyzed urban region. The variable *b*_*c*_ here then corresponds to the network average centrality in the analyzed region.

To describe a city’s road-based public transportation system we use the line network of buses and trams, which length in the network is *LBN*. Line network in this context means that we use each direction of each line. All public transportation data, except for Greater Manchester and Cagliari, which were downloaded from their OpenData Portal, were downloaded from the OVERPASS API (OSM based). We only consider services that run within a city’s local public transportation network, e.g. in Zurich all lines belonging to the Zürcher Verkehrsverbund (ZVV). We remove all long-distance services (buses) because we expect the long-distance services to run less frequently. In the case that more than two routes per line appear (e.g. due to deviations in the time table), we select for each direction the longest route. School bus services are not considered in this data set. We measure the average headway *HW* in the network by random OD requests in google transit directions in the network and extract the headway attribute thereof in peak hour. Then, we calculate the bus production density by *B* = *LBN*/*HW*/*A*.

## Supplementary Information

Supplementary Information

## Data Availability

All relevant data is available from the authors upon request, after consultation with those cities where a non-disclosure agreement applies. Freely accessible data will be hosted at ETH’s open access data archive (can be accessed via http://www.ivt.ethz.ch/forschung/mfd.html).
