# Supplementary material for: Understanding traffic capacity of urban networks
Source: Sci Rep. 2019 Nov 8;9:16283. doi: 10.1038/s41598-019-51539-5 (PMC6841661; doi:10.1038/s41598-019-51539-5)
Supplement: Supplementary file 1 — Supplementary Information [file 41598_2019_51539_MOESM1_ESM.pdf]

Understanding traffic capacity of urban networks

Allister Loder, Lukas Ambühl, Monica Menendez, Kay W. Axhausen

August 2019

Supplementary information

No	City	Country	Population [1000]	Detectors	Days	Regions
1	Augsburg	Germany	277	777	20	1
2	Basel	Switzerland	167	83	7	1
3	Bern	Switzerland	129	769	7	1
4	Birmingham	United Kingdom	1097	114	6	1
5	Bolton	United Kingdom	128	202	22	1
6	Bordeaux	France	754	591	7	4
7	Bremen	Germany	549	583	14	2
8	Cagliari	Italy	154	133	50	1
9	Constance	Germany	81	129	7	1
10	Darmstadt	Germany	150	393	5	1
11	Duisburg	Germany	487	590	14	1
12	Essen	Germany	570	38	36	1
13	Frankfurt	Germany	701	112	1	1
14	Graz	Austria	270	300	10	1
15	Groningen	Netherlands	198	55	6	1
16	Hamburg	Germany	1746	419	105	1
17	Innsbruck	Austria	125	49	30	1
18	Kassel	Germany	194	601	4	3
19	London	United Kingdom	8478	5804	22	16
20	Los Angeles	USA	3970	4072	14	3
21	Luzern	Switzerland	81	159	361	1
22	Madrid	Spain	3142	2123	20	10
23	Manchester	United Kingdom	517	221	22	1
24	Marseille	France	1054	178	32	2
25	Melbourne	Australia	4820	1649	15	3
26	Munich	Germany	1408	548	1	2
27	Paris	France	3236	513	366	4
28	Rotterdam	Netherlands	618	227	6	1
29	Santander	Spain	176	378	3	2
30	Speyer	Germany	50	199	14	1
31	Strasbourg	France	228	220	25	1
32	Stuttgart	Germany	604	298	8	1
33	Taipei	Taiwan	2674	445	14	6
34	Tokyo	Japan	9273	2111	30	9
35	Torino	Italy	902	787	21	3
36	Toronto	Canada	2809	298	61	2
37	Toulouse	France	747	910	7	4
38	Utrecht	Netherlands	328	1072	4	1
39	Vilnius	Lithuania	540	581	1	2
40	Wolfsburg	Germany	122	405	14	1
41	Zurich	Switzerland	385	1225	7	7

Table 1: Sample statistics

		Dependent variables			
		Critical accumulation n^* (veh km^{-2})		Capacity P^* (veh-km h^{-1} km^{-2})	
Covariates	Unit	β	t-value	β	t-value
Constant		-269.5	(-3.23)	769.7	(1.71)
n^*	(veh km^{-2})			18.80	(20.77)
R	(lane-km km^{-2})	25.63	(12.85)		
$R \times I$	(lane-km km^{-2} $\times$ LSA km^{-1})	-5.748	(-2.08)		
b_c	(-)	-128.9	(-3.52)		
B	(bus-km h^{-1} km^{-2})	0.255	(1.41)	-5.60	(-3.71)
N		107		107	
Adj. R^2		0.88		0.93	

Elasticity	ε	p-value	$H_0 : \varepsilon = 1$
$\partial \log P^* / \partial \log n^*$	1.00	0.989	not reject
$\partial \log n^* / \partial \log R$	0.85	0.000	reject

Table 2: Robustness check, critical point model without observation weights. The probability that n^* is exogenous is $p = 0.0000$.

		Dependent variables			
		Critical accumulation n^* (veh km^{-2})		Capacity P^* (veh-km h^{-1} km^{-2})	
Covariates	Unit	β	t-value	β	t-value
Constant		3.359	(23.53)	2.904	(8.00)
n^*	(veh km^{-2})			1.01	(18.92)
R	(lane-km km^{-2})	0.825	(25.46)		
$R \times I$	(lane-km km^{-2} $\times$ LSA km^{-1})	-0.018	(-0.590)		
b_c	(-)	-0.183	(-4.50)		
B	(bus-km h^{-1} km^{-2})	0.032	(1.67)	-0.054	(-2.87)
N		107		107	
Adj. R^2		0.89		0.91	

Elasticity	ε	p-value	$H_0 : \varepsilon = 1$
$\partial \log P^* / \partial \log n^*$	1.01	0.876	not reject
$\partial \log n^* / \partial \log R$	0.82	0.000	reject

Table 3: Robustness check, critical point model in log-log specification. Variables P^*, n^*, B and R have been log-transformed, while the others have been left unchanged. The probability that n^* is exogenous is $p = 0.0000$.

		Dependent variables			
		Critical density k^* (veh lane-km^{-1})		Critical density k^* (veh lane-km^{-1})	
		with weights		without weights	
Covariates	Unit	β	t-value	β	t-value
Constant		18.90	(6.53)	20.08	(7.07)
R	(lane-km km^{-2})	-0.162	(-3.53)	-0.120	(-2.04)
b_c	(-)	-4.928	(-4.38)	-4.188	(-3.62)
N		107		107	
Adj. R^2		0.25		0.16	

Elasticity		ε	p-value	$H_0 : \varepsilon = 0$
With weights	$\partial \log k^* / \partial \log R$	-0.143	0.001	reject
Without weights	$\partial \log k^* / \partial \log R$	-0.102	0.035	reject

Table 4: Robustness check, critical density equation in veh lane-km^{-1} formulation, i.e. not production normalized by unit area. The effects of I and B are statistically not significant. Note that the elasticity here is negative as the dependent variable is density, not accumulation, i.e. a sublinear increase in accumulation means that the average density decreases.

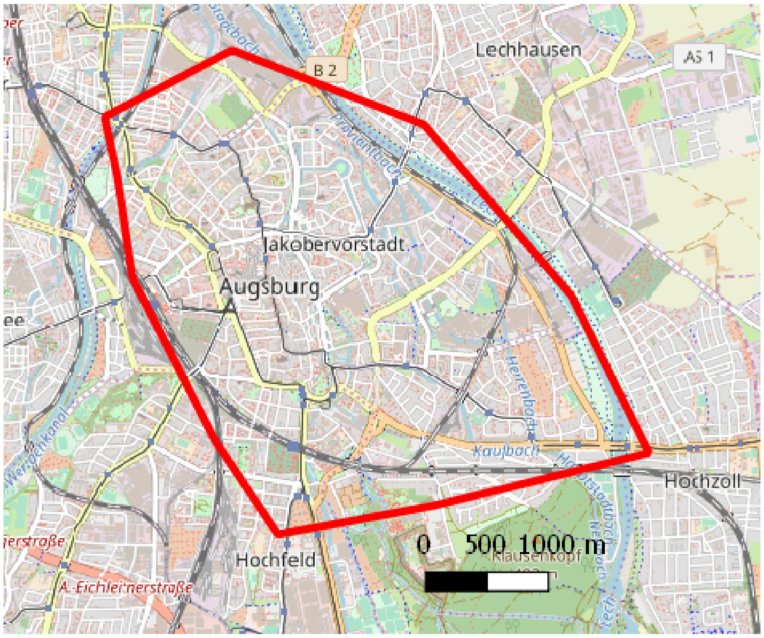

(a) Network exhibit

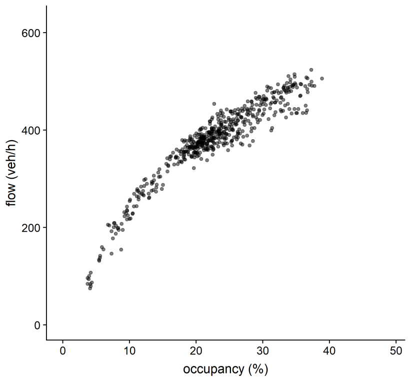

(b) Raw data

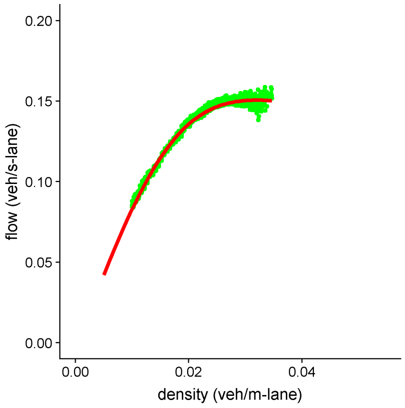

(c) Upper bound and fitted curve

Figure 1: Augsburg Region I. (**a**) shows the network exhibit used for the MFD estimation and calculation of network topology variables. The map is oriented to the north and is obtained from OpenStreetMap. (**b**) presents the scatter plot of the raw, i.e., unfiltered, data of the network shown in **a**. This includes all malfunctioning detectors, oversampling of lanes, detectors on residential roads etc. (**c**) shows the upper-bound of the estimated MFD and the fitted curve based on the log-exp function by Ambühl et al. (2018). Flow and density are per lane. Occupancy is in the interval 0 to 1 and means the fraction of time in the observation interval a detector is covered by vehicles, i.e., it approximates density.

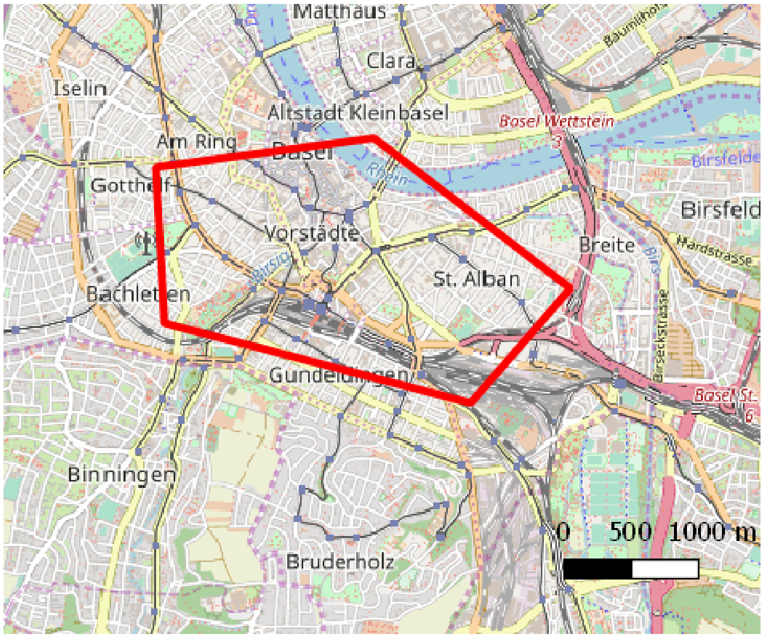

(a) Network exhibit

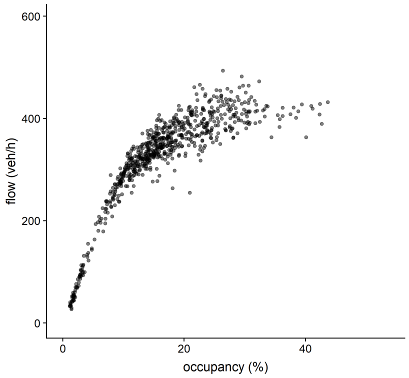

(b) Raw data

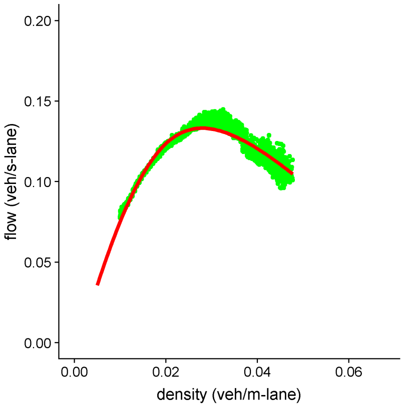

(c) Upper bound and fitted curve

Figure 2: Basel Region I. (**a**) shows the network exhibit used for the MFD estimation and calculation of network topology variables. The map is oriented to the north and is obtained from OpenStreetMap. (**b**) presents the scatter plot of the raw, i.e., unfiltered, data of the network shown in **a**. This includes all malfunctioning detectors, oversampling of lanes, detectors on residential roads etc. (**c**) shows the upper-bound of the estimated MFD and the fitted curve based on the log-exp function by Ambühl et al. (2018). Flow and density are per lane. Occupancy is in the interval 0 to 1 and means the fraction of time in the observation interval a detector is covered by vehicles, i.e., it approximates density.

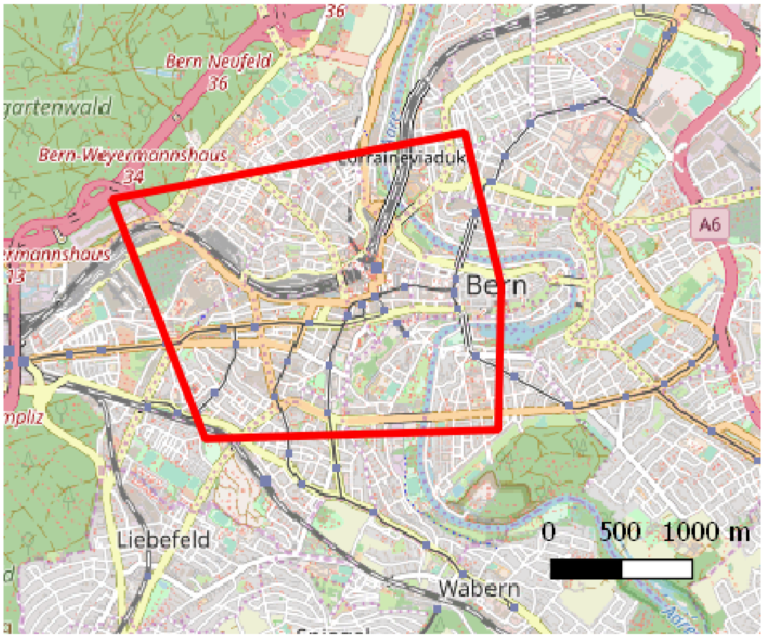

(a) Network exhibit

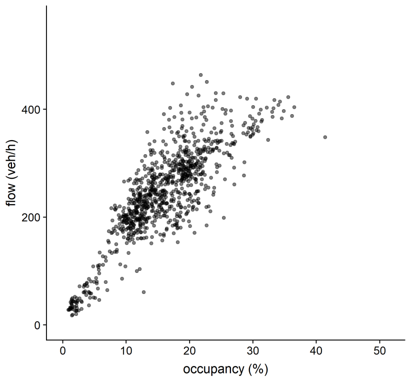

(b) Raw data

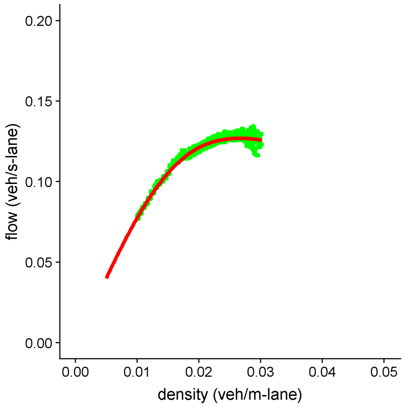

(c) Upper bound and fitted curve

Figure 3: Bern Region I. (**a**) shows the network exhibit used for the MFD estimation and calculation of network topology variables. The map is oriented to the north and is obtained from OpenStreetMap. (**b**) presents the scatter plot of the raw, i.e., unfiltered, data of the network shown in **a**. This includes all malfunctioning detectors, oversampling of lanes, detectors on residential roads etc. (**c**) shows the upper-bound of the estimated MFD and the fitted curve based on the log-exp function by Ambühl et al. (2018). Flow and density are per lane. Occupancy is in the interval 0 to 1 and means the fraction of time in the observation interval a detector is covered by vehicles, i.e., it approximates density.

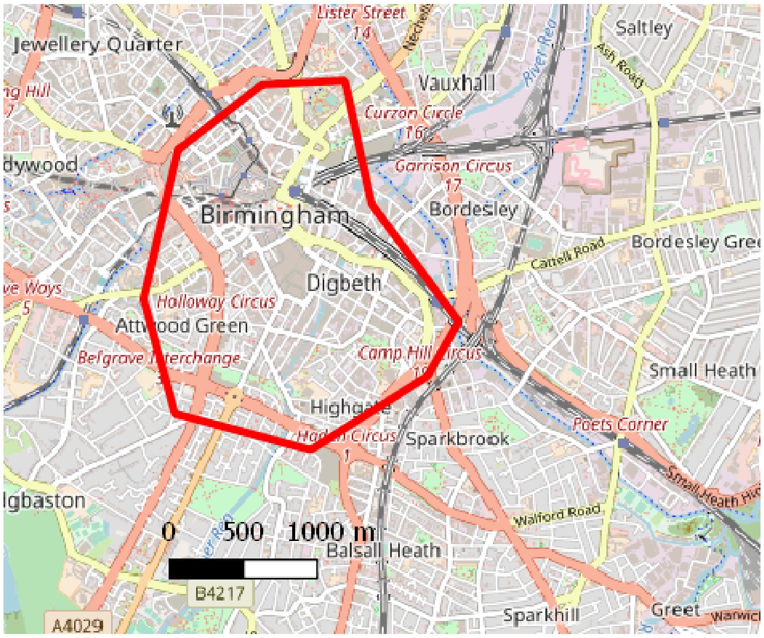

(a) Network exhibit

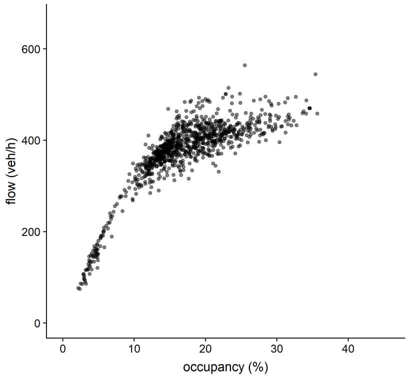

(b) Raw data

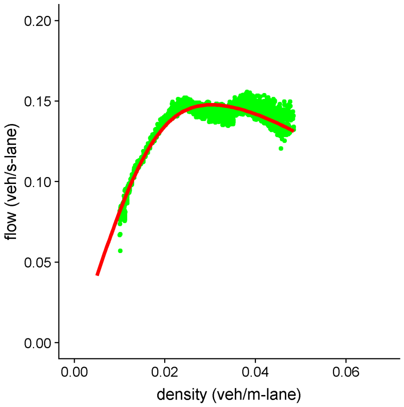

(c) Upper bound and fitted curve

Figure 4: Birmingham Region I. (**a**) shows the network exhibit used for the MFD estimation and calculation of network topology variables. The map is oriented to the north and is obtained from OpenStreetMap. (**b**) presents the scatter plot of the raw, i.e., unfiltered, data of the network shown in **a**. This includes all malfunctioning detectors, oversampling of lanes, detectors on residential roads etc. (**c**) shows the upper-bound of the estimated MFD and the fitted curve based on the log-exp function by Ambühl et al. (2018). Flow and density are per lane. Occupancy is in the interval 0 to 1 and means the fraction of time in the observation interval a detector is covered by vehicles, i.e., it approximates density.

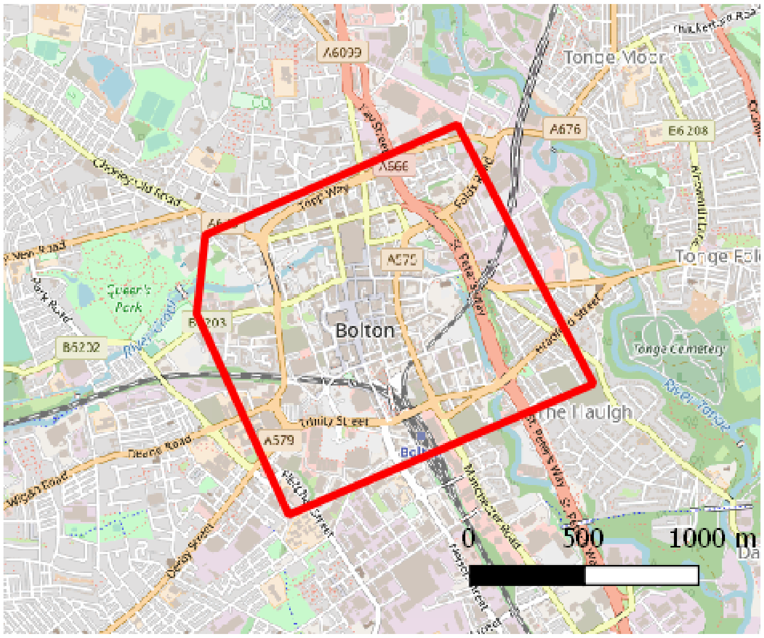

(a) Network exhibit

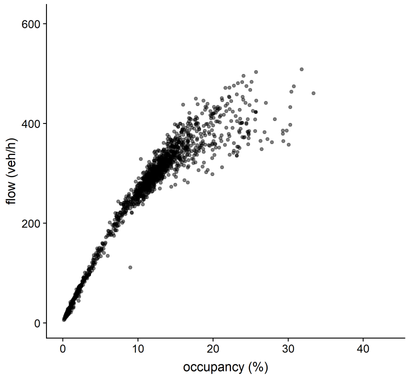

(b) Raw data

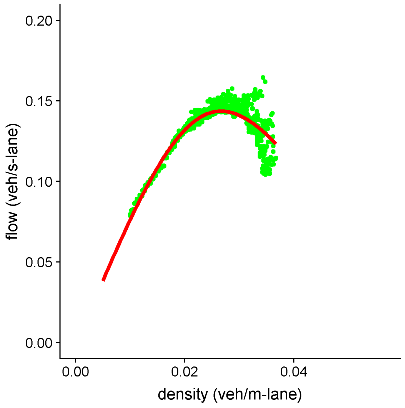

(c) Upper bound and fitted curve

Figure 5: Bolton Region I. (**a**) shows the network exhibit used for the MFD estimation and calculation of network topology variables. The map is oriented to the north and is obtained from OpenStreetMap. (**b**) presents the scatter plot of the raw, i.e., unfiltered, data of the network shown in **a**. This includes all malfunctioning detectors, oversampling of lanes, detectors on residential roads etc. (**c**) shows the upper-bound of the estimated MFD and the fitted curve based on the log-exp function by Ambühl et al. (2018). Flow and density are per lane. Occupancy is in the interval 0 to 1 and means the fraction of time in the observation interval a detector is covered by vehicles, i.e., it approximates density.

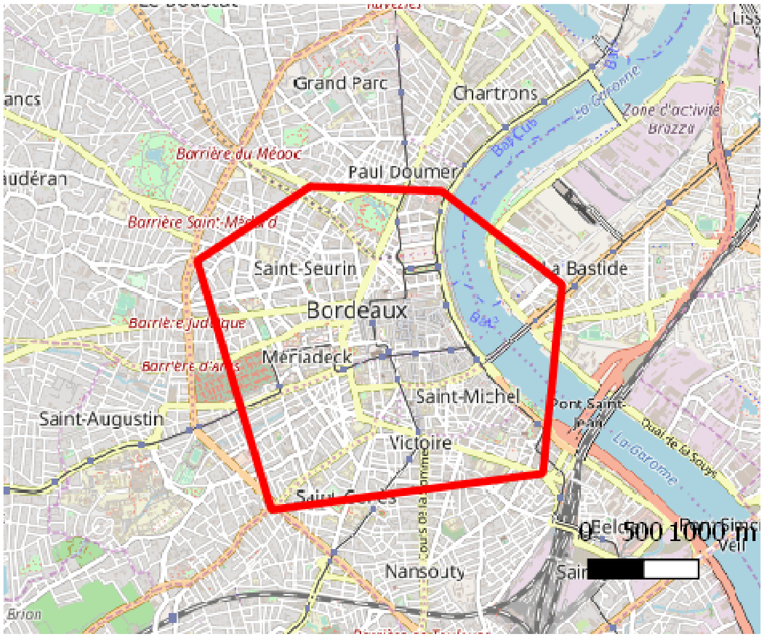

(a) Network exhibit

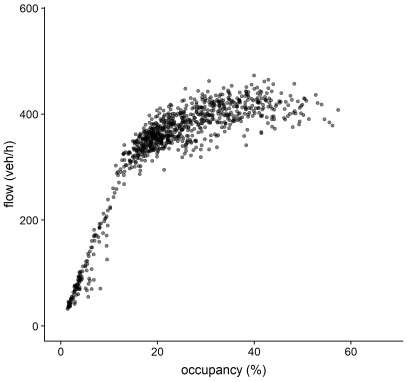

(b) Raw data

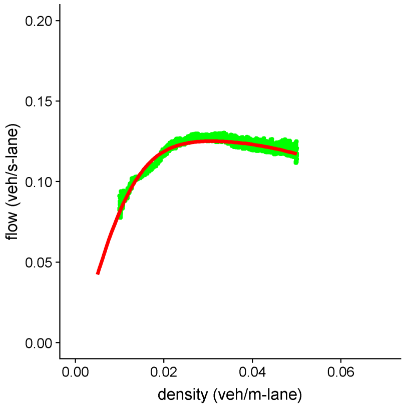

(c) Upper bound and fitted curve

Figure 6: Bordeaux Region I. (**a**) shows the network exhibit used for the MFD estimation and calculation of network topology variables. The map is oriented to the north and is obtained from OpenStreetMap. (**b**) presents the scatter plot of the raw, i.e., unfiltered, data of the network shown in **a**. This includes all malfunctioning detectors, oversampling of lanes, detectors on residential roads etc. (**c**) shows the upper-bound of the estimated MFD and the fitted curve based on the log-exp function by Ambühl et al. (2018). Flow and density are per lane. Occupancy is in the interval 0 to 1 and means the fraction of time in the observation interval a detector is covered by vehicles, i.e., it approximates density.

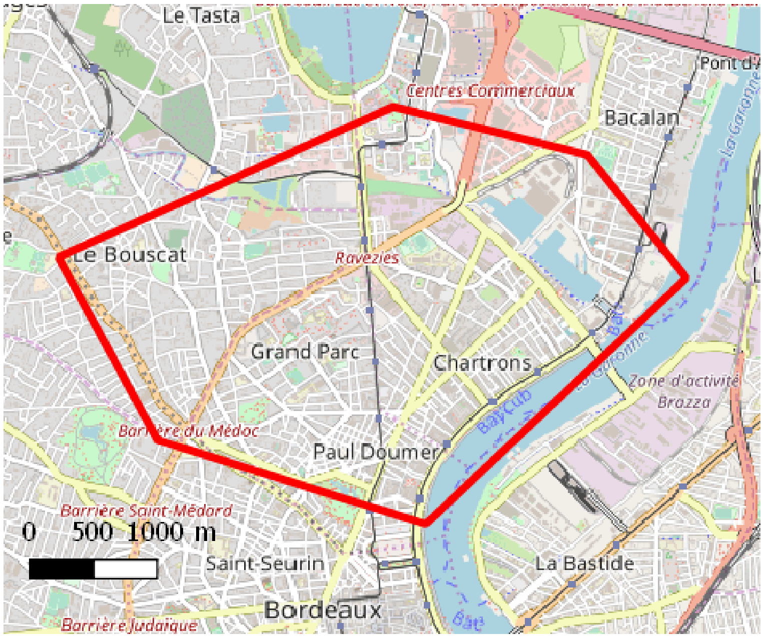

(a) Network exhibit

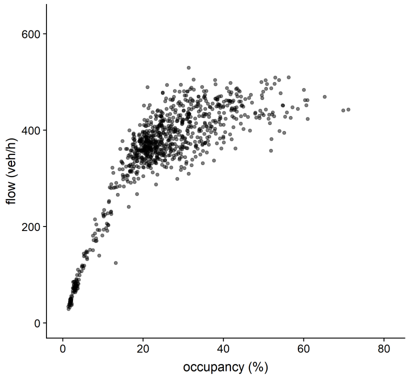

(b) Raw data

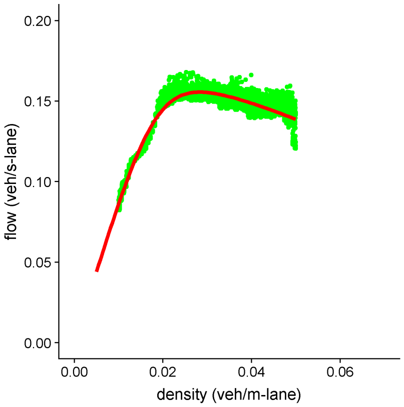

(c) Upper bound and fitted curve

Figure 7: Bordeaux Region II. (**a**) shows the network exhibit used for the MFD estimation and calculation of network topology variables. The map is oriented to the north and is obtained from OpenStreetMap. (**b**) presents the scatter plot of the raw, i.e., unfiltered, data of the network shown in **a**. This includes all malfunctioning detectors, oversampling of lanes, detectors on residential roads etc. (**c**) shows the upper-bound of the estimated MFD and the fitted curve based on the log-exp function by Ambühl et al. (2018). Flow and density are per lane. Occupancy is in the interval 0 to 1 and means the fraction of time in the observation interval a detector is covered by vehicles, i.e., it approximates density.

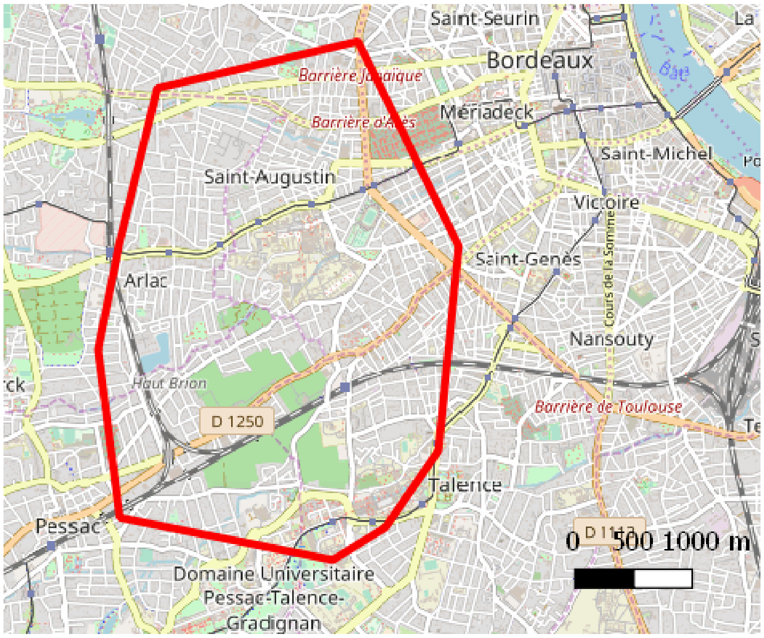

(a) Network exhibit

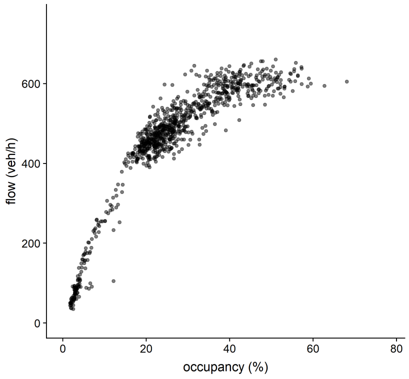

(b) Raw data

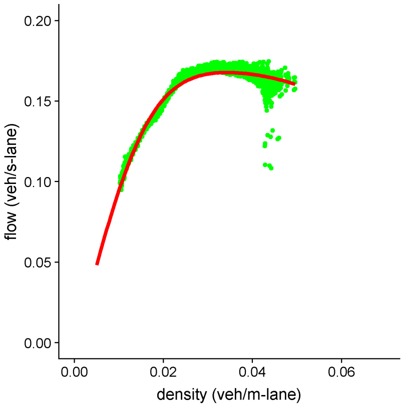

(c) Upper bound and fitted curve

Figure 8: Bordeaux Region III. (**a**) shows the network exhibit used for the MFD estimation and calculation of network topology variables. The map is oriented to the north and is obtained from OpenStreetMap. (**b**) presents the scatter plot of the raw, i.e., unfiltered, data of the network shown in **a**. This includes all malfunctioning detectors, oversampling of lanes, detectors on residential roads etc. (**c**) shows the upper-bound of the estimated MFD and the fitted curve based on the log-exp function by Ambühl et al. (2018). Flow and density are per lane. Occupancy is in the interval 0 to 1 and means the fraction of time in the observation interval a detector is covered by vehicles, i.e., it approximates density.

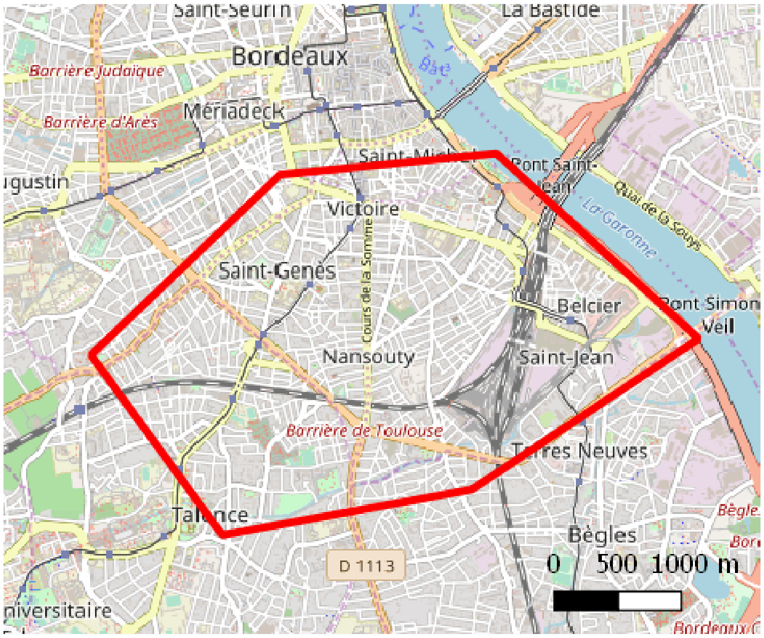

(a) Network exhibit

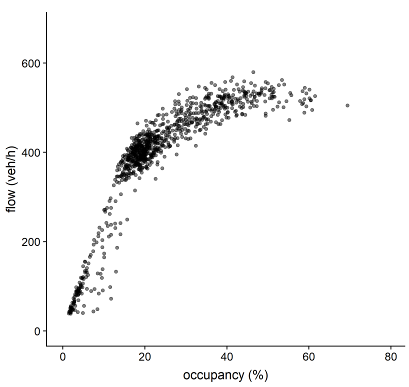

(b) Raw data

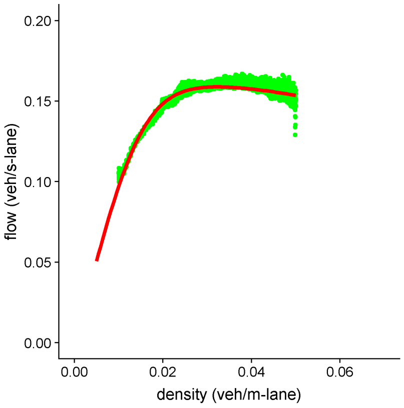

(c) Upper bound and fitted curve

Figure 9: Bordeaux Region IV. (**a**) shows the network exhibit used for the MFD estimation and calculation of network topology variables. The map is oriented to the north and is obtained from OpenStreetMap. (**b**) presents the scatter plot of the raw, i.e., unfiltered, data of the network shown in **a**. This includes all malfunctioning detectors, oversampling of lanes, detectors on residential roads etc. (**c**) shows the upper-bound of the estimated MFD and the fitted curve based on the log-exp function by Ambühl et al. (2018). Flow and density are per lane. Occupancy is in the interval 0 to 1 and means the fraction of time in the observation interval a detector is covered by vehicles, i.e., it approximates density.

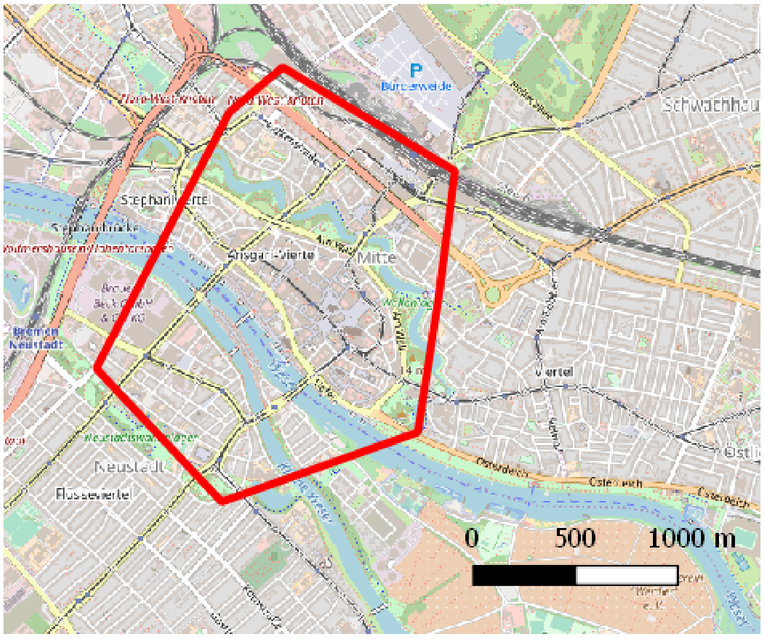

(a) Network exhibit

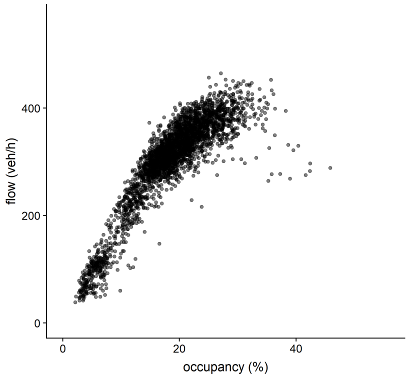

(b) Raw data

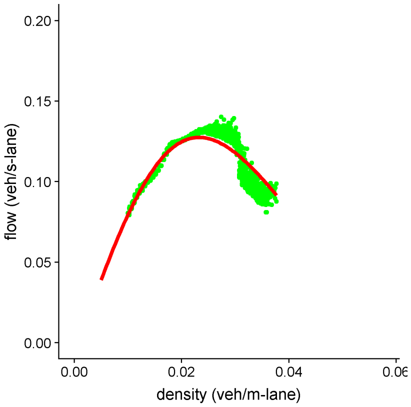

(c) Upper bound and fitted curve

Figure 10: Bremen Region I. (**a**) shows the network exhibit used for the MFD estimation and calculation of network topology variables. The map is oriented to the north and is obtained from OpenStreetMap. (**b**) presents the scatter plot of the raw, i.e., unfiltered, data of the network shown in **a**. This includes all malfunctioning detectors, oversampling of lanes, detectors on residential roads etc. (**c**) shows the upper-bound of the estimated MFD and the fitted curve based on the log-exp function by Ambühl et al. (2018). Flow and density are per lane. Occupancy is in the interval 0 to 1 and means the fraction of time in the observation interval a detector is covered by vehicles, i.e., it approximates density.

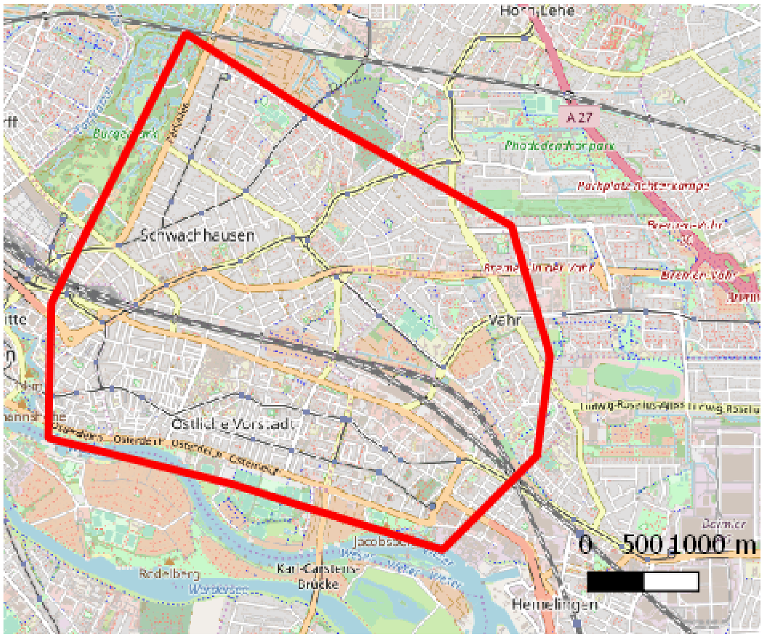

(a) Network exhibit

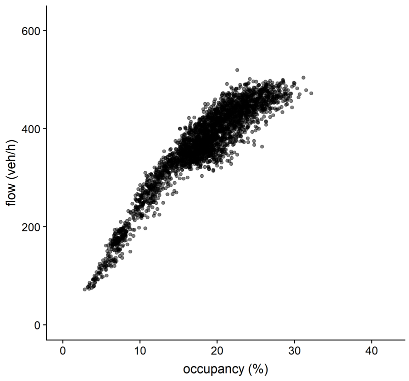

(b) Raw data

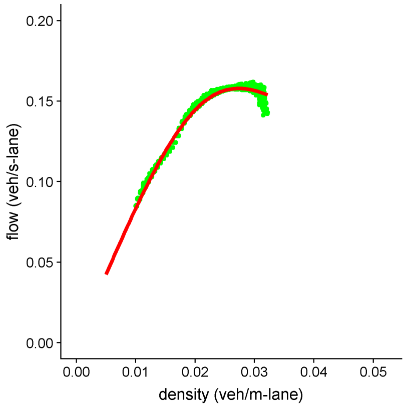

(c) Upper bound and fitted curve

Figure 11: Bremen Region II. (**a**) shows the network exhibit used for the MFD estimation and calculation of network topology variables. The map is oriented to the north and is obtained from OpenStreetMap. (**b**) presents the scatter plot of the raw, i.e., unfiltered, data of the network shown in **a**. This includes all malfunctioning detectors, oversampling of lanes, detectors on residential roads etc. (**c**) shows the upper-bound of the estimated MFD and the fitted curve based on the log-exp function by Ambühl et al. (2018). Flow and density are per lane. Occupancy is in the interval 0 to 1 and means the fraction of time in the observation interval a detector is covered by vehicles, i.e., it approximates density.

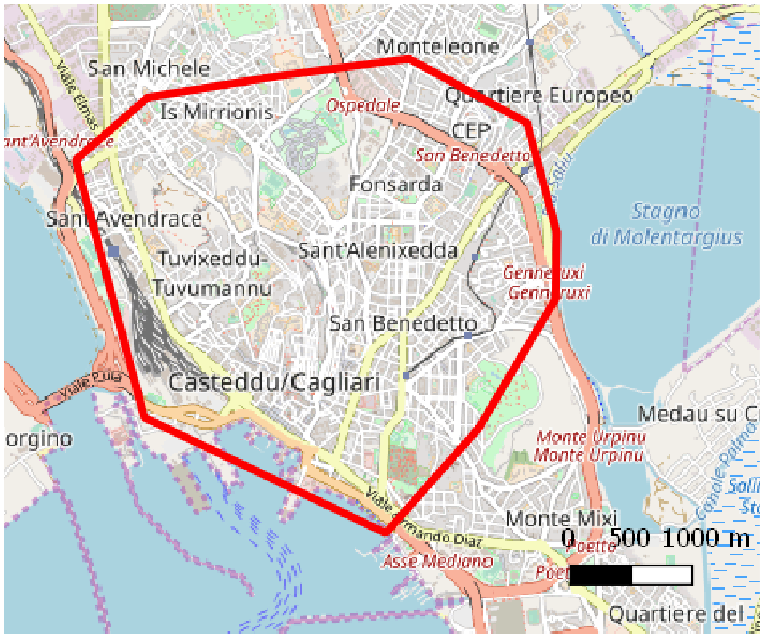

(a) Network exhibit

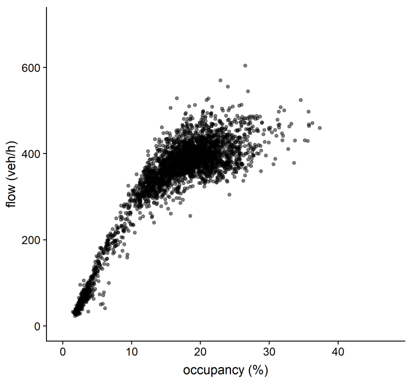

(b) Raw data

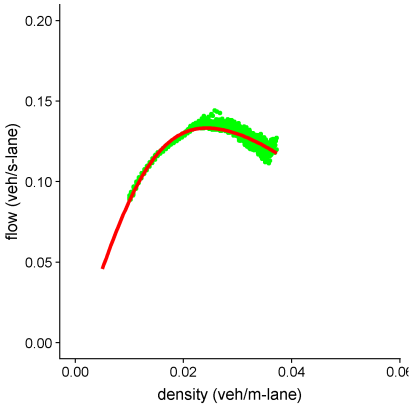

(c) Upper bound and fitted curve

Figure 12: Cagliari Region I. (**a**) shows the network exhibit used for the MFD estimation and calculation of network topology variables. The map is oriented to the north and is obtained from OpenStreetMap. (**b**) presents the scatter plot of the raw, i.e., unfiltered, data of the network shown in **a**. This includes all malfunctioning detectors, oversampling of lanes, detectors on residential roads etc. (**c**) shows the upper-bound of the estimated MFD and the fitted curve based on the log-exp function by Ambühl et al. (2018). Flow and density are per lane. Occupancy is in the interval 0 to 1 and means the fraction of time in the observation interval a detector is covered by vehicles, i.e., it approximates density.

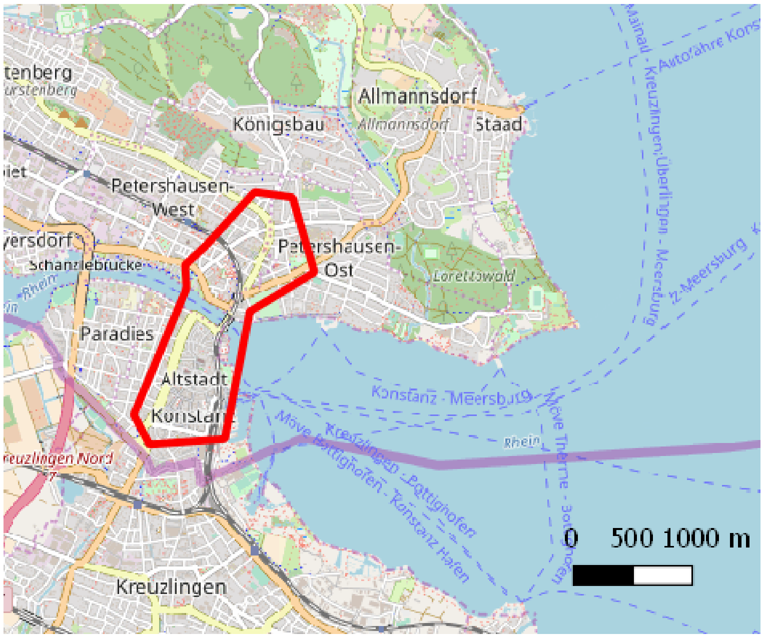

(a) Network exhibit

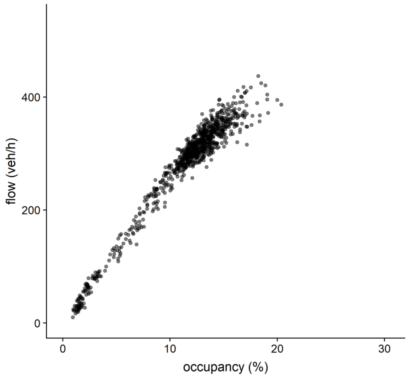

(b) Raw data

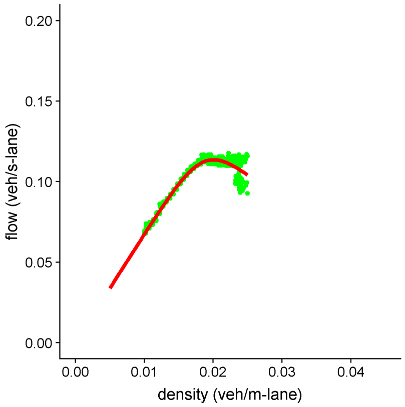

(c) Upper bound and fitted curve

Figure 13: Constance Region I. (**a**) shows the network exhibit used for the MFD estimation and calculation of network topology variables. The map is oriented to the north and is obtained from OpenStreetMap. (**b**) presents the scatter plot of the raw, i.e., unfiltered, data of the network shown in **a**. This includes all malfunctioning detectors, oversampling of lanes, detectors on residential roads etc. (**c**) shows the upper-bound of the estimated MFD and the fitted curve based on the log-exp function by Ambühl et al. (2018). Flow and density are per lane. Occupancy is in the interval 0 to 1 and means the fraction of time in the observation interval a detector is covered by vehicles, i.e., it approximates density.

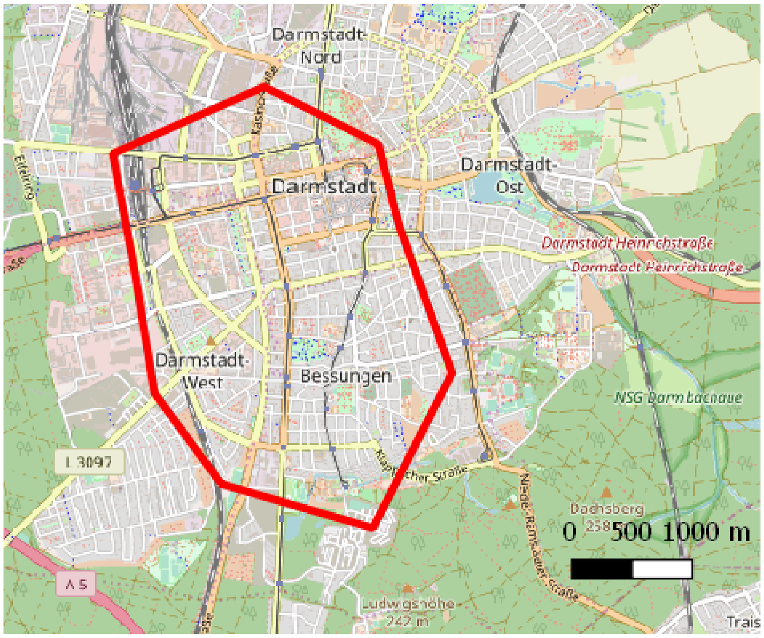

(a) Network exhibit

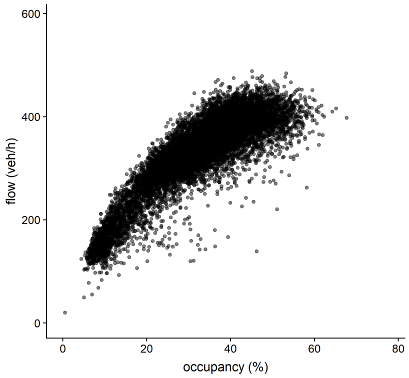

(b) Raw data

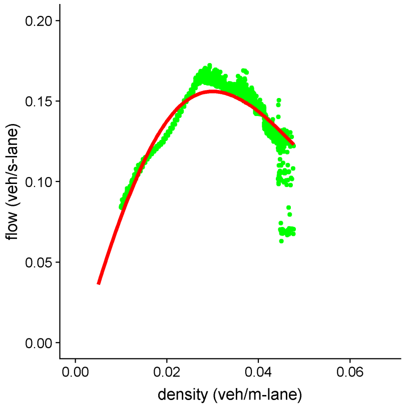

(c) Upper bound and fitted curve

Figure 14: Darmstadt Region I. (**a**) shows the network exhibit used for the MFD estimation and calculation of network topology variables. The map is oriented to the north and is obtained from OpenStreetMap. (**b**) presents the scatter plot of the raw, i.e., unfiltered, data of the network shown in **a**. This includes all malfunctioning detectors, oversampling of lanes, detectors on residential roads etc. (**c**) shows the upper-bound of the estimated MFD and the fitted curve based on the log-exp function by Ambühl et al. (2018). Flow and density are per lane. Occupancy is in the interval 0 to 1 and means the fraction of time in the observation interval a detector is covered by vehicles, i.e., it approximates density.

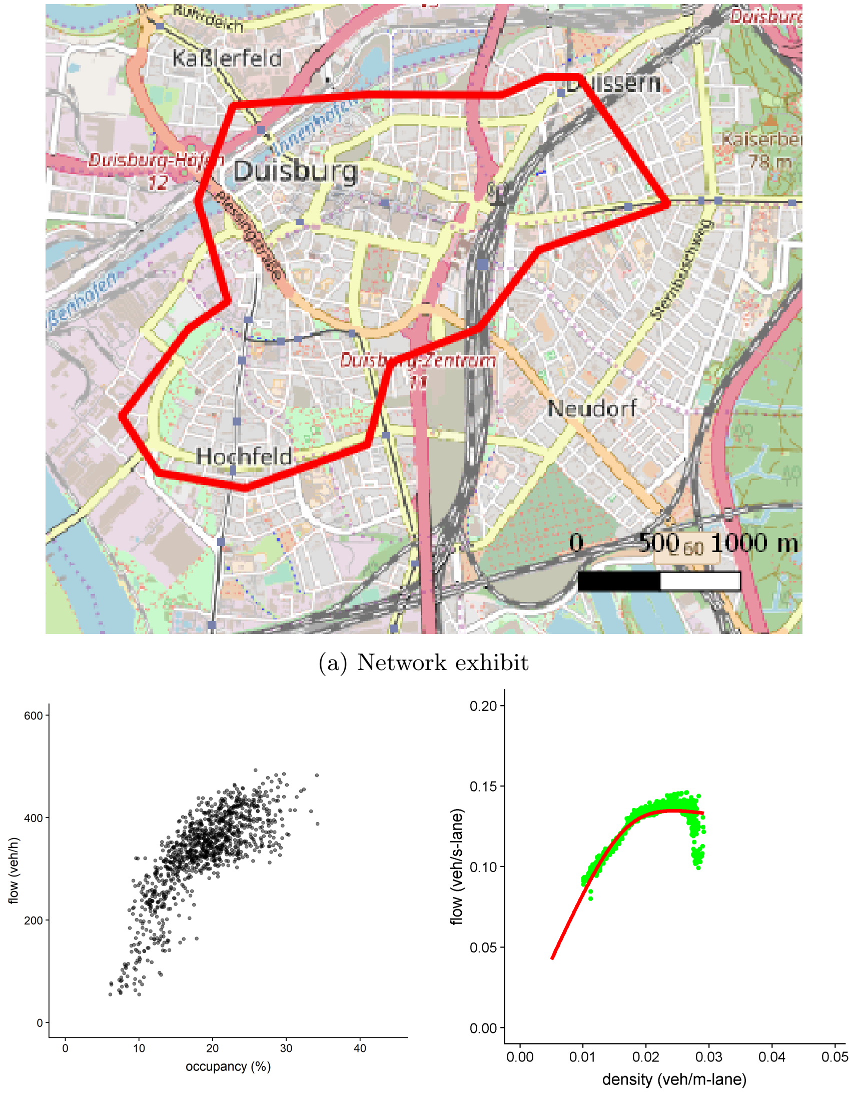

(a) Network exhibit

(b) Raw data

(c) Upper bound and fitted curve

Figure 15: Duisburg Region I. (**a**) shows the network exhibit used for the MFD estimation and calculation of network topology variables. The map is oriented to the north and is obtained from OpenStreetMap. (**b**) presents the scatter plot of the raw, i.e., unfiltered, data of the network shown in **a**. This includes all malfunctioning detectors, oversampling of lanes, detectors on residential roads etc. (**c**) shows the upper-bound of the estimated MFD and the fitted curve based on the log-exp function by Ambühl et al. (2018). Flow and density are per lane. Occupancy is in the interval 0 to 1 and means the fraction of time in the observation interval a detector is covered by vehicles, i.e., it approximates density.

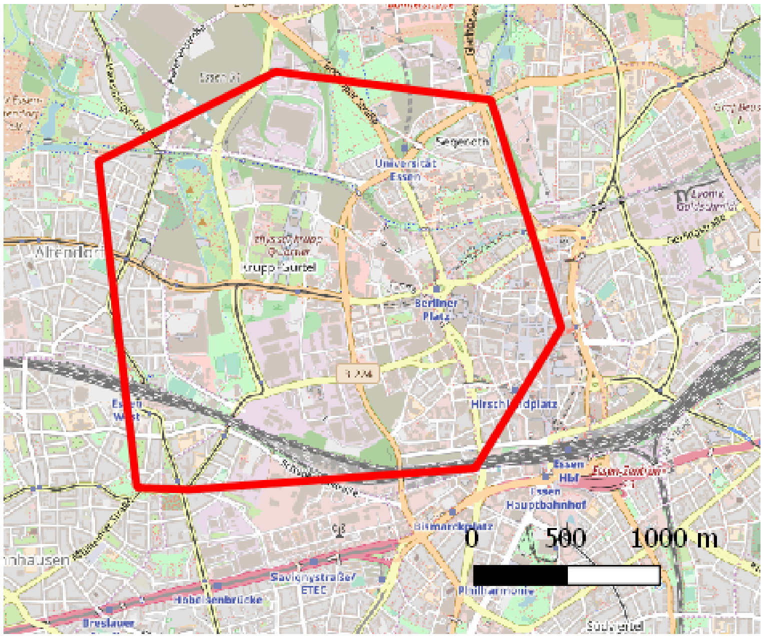

(a) Network exhibit

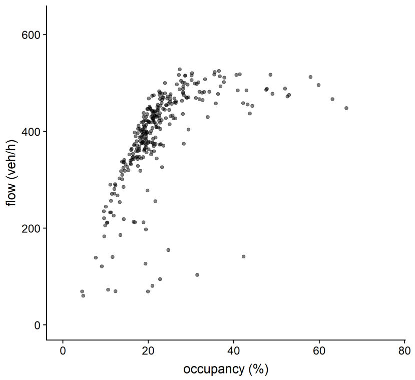

(b) Raw data

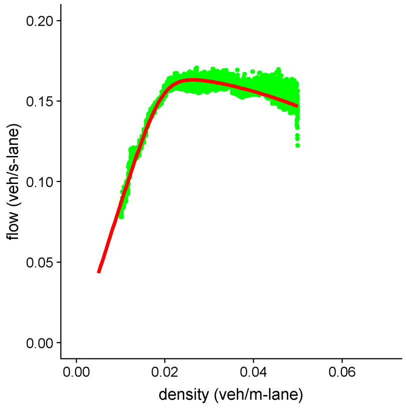

(c) Upper bound and fitted curve

Figure 16: Essen Region I. (**a**) shows the network exhibit used for the MFD estimation and calculation of network topology variables. The map is oriented to the north and is obtained from OpenStreetMap. (**b**) presents the scatter plot of the raw, i.e., unfiltered, data of the network shown in **a**. This includes all malfunctioning detectors, oversampling of lanes, detectors on residential roads etc. (**c**) shows the upper-bound of the estimated MFD and the fitted curve based on the log-exp function by Ambühl et al. (2018). Flow and density are per lane. Occupancy is in the interval 0 to 1 and means the fraction of time in the observation interval a detector is covered by vehicles, i.e., it approximates density.

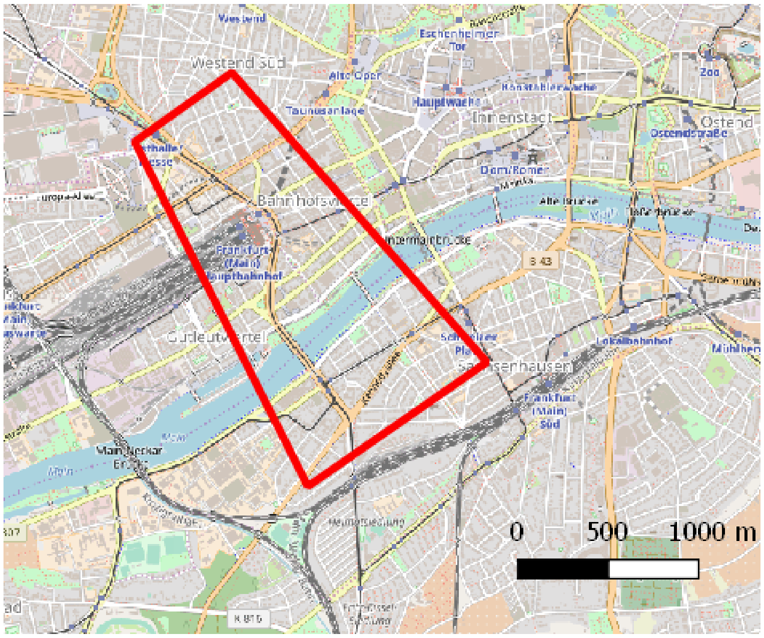

(a) Network exhibit

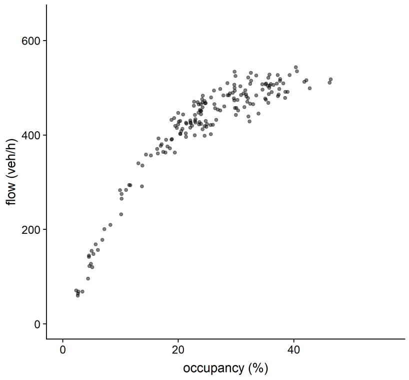

(b) Raw data

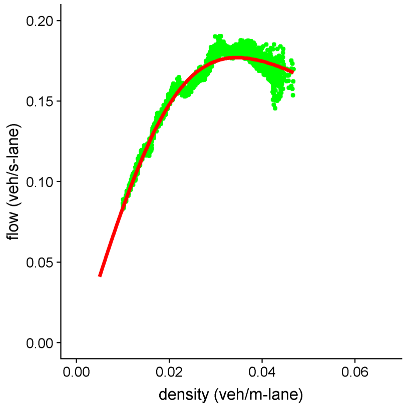

(c) Upper bound and fitted curve

Figure 17: Frankfurt Region I. (**a**) shows the network exhibit used for the MFD estimation and calculation of network topology variables. The map is oriented to the north and is obtained from OpenStreetMap. (**b**) presents the scatter plot of the raw, i.e., unfiltered, data of the network shown in **a**. This includes all malfunctioning detectors, oversampling of lanes, detectors on residential roads etc. (**c**) shows the upper-bound of the estimated MFD and the fitted curve based on the log-exp function by Ambühl et al. (2018). Flow and density are per lane. Occupancy is in the interval 0 to 1 and means the fraction of time in the observation interval a detector is covered by vehicles, i.e., it approximates density.

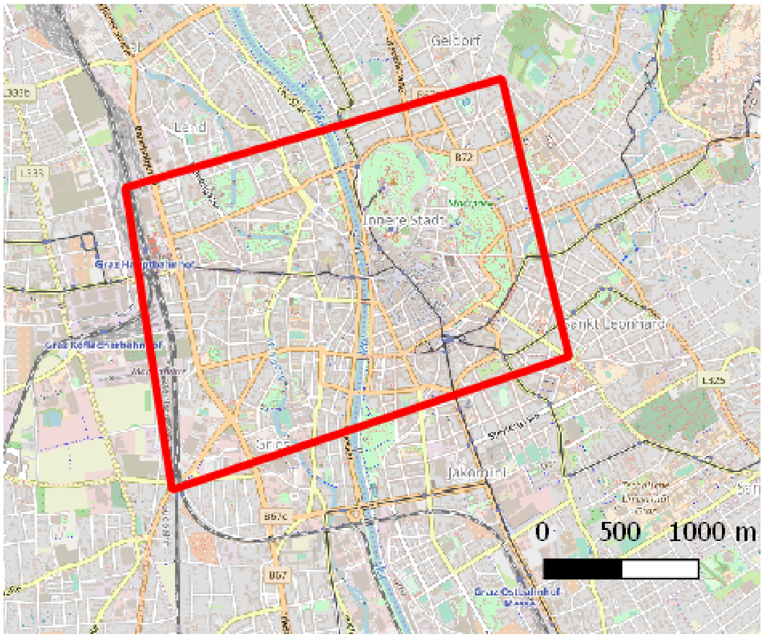

(a) Network exhibit

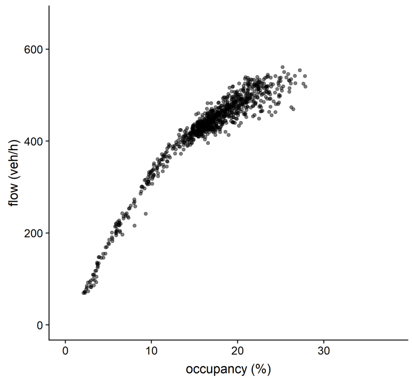

(b) Raw data

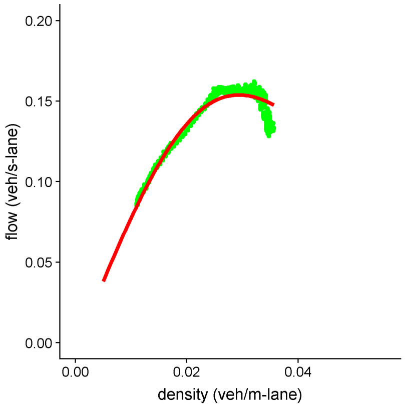

(c) Upper bound and fitted curve

Figure 18: Graz Region I. (**a**) shows the network exhibit used for the MFD estimation and calculation of network topology variables. The map is oriented to the north and is obtained from OpenStreetMap. (**b**) presents the scatter plot of the raw, i.e., unfiltered, data of the network shown in **a**. This includes all malfunctioning detectors, oversampling of lanes, detectors on residential roads etc. (**c**) shows the upper-bound of the estimated MFD and the fitted curve based on the log-exp function by Ambühl et al. (2018). Flow and density are per lane. Occupancy is in the interval 0 to 1 and means the fraction of time in the observation interval a detector is covered by vehicles, i.e., it approximates density.

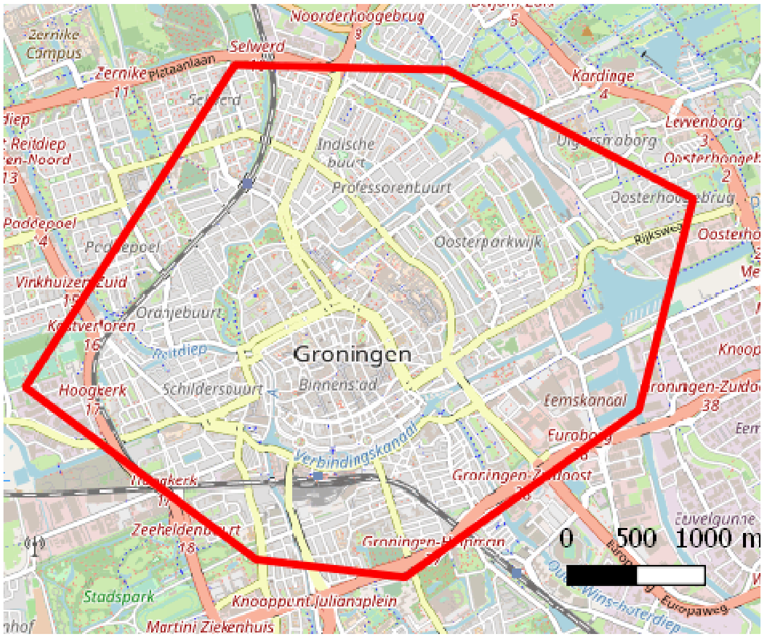

(a) Network exhibit

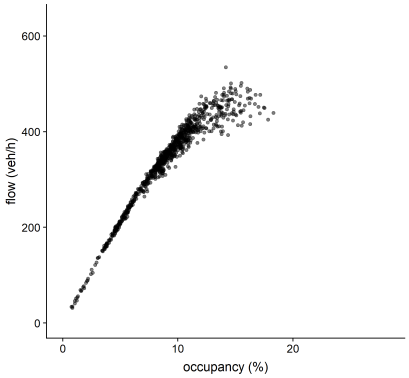

(b) Raw data

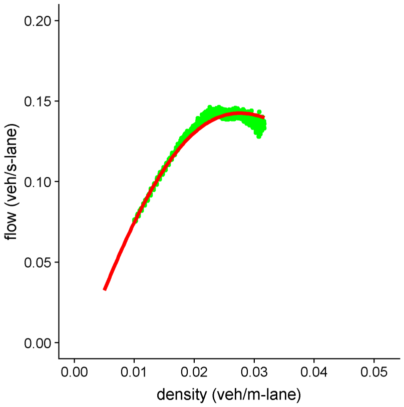

(c) Upper bound and fitted curve

Figure 19: Groningen Region I. (**a**) shows the network exhibit used for the MFD estimation and calculation of network topology variables. The map is oriented to the north and is obtained from OpenStreetMap. (**b**) presents the scatter plot of the raw, i.e., unfiltered, data of the network shown in **a**. This includes all malfunctioning detectors, oversampling of lanes, detectors on residential roads etc. (**c**) shows the upper-bound of the estimated MFD and the fitted curve based on the log-exp function by Ambühl et al. (2018). Flow and density are per lane. Occupancy is in the interval 0 to 1 and means the fraction of time in the observation interval a detector is covered by vehicles, i.e., it approximates density.

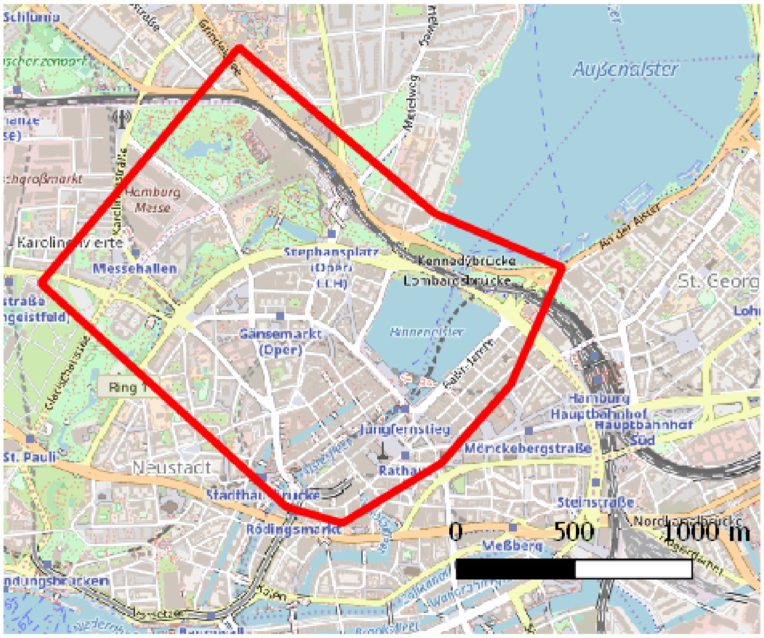

(a) Network exhibit

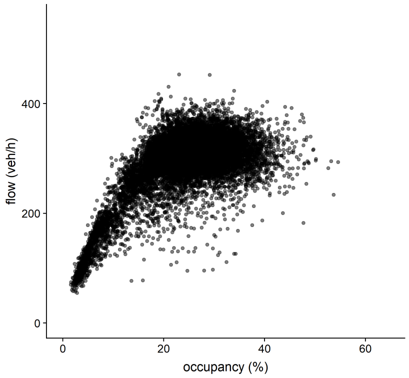

(b) Raw data

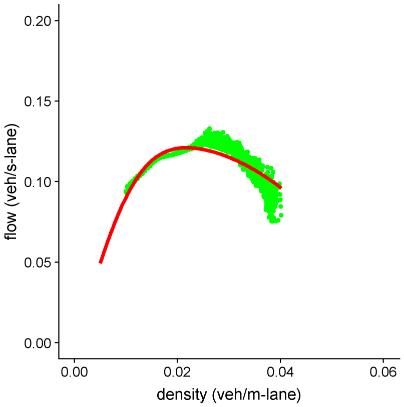

(c) Upper bound and fitted curve

Figure 20: Hamburg Region I. (**a**) shows the network exhibit used for the MFD estimation and calculation of network topology variables. The map is oriented to the north and is obtained from OpenStreetMap. (**b**) presents the scatter plot of the raw, i.e., unfiltered, data of the network shown in **a**. This includes all malfunctioning detectors, oversampling of lanes, detectors on residential roads etc. (**c**) shows the upper-bound of the estimated MFD and the fitted curve based on the log-exp function by Ambühl et al. (2018). Flow and density are per lane. Occupancy is in the interval 0 to 1 and means the fraction of time in the observation interval a detector is covered by vehicles, i.e., it approximates density.

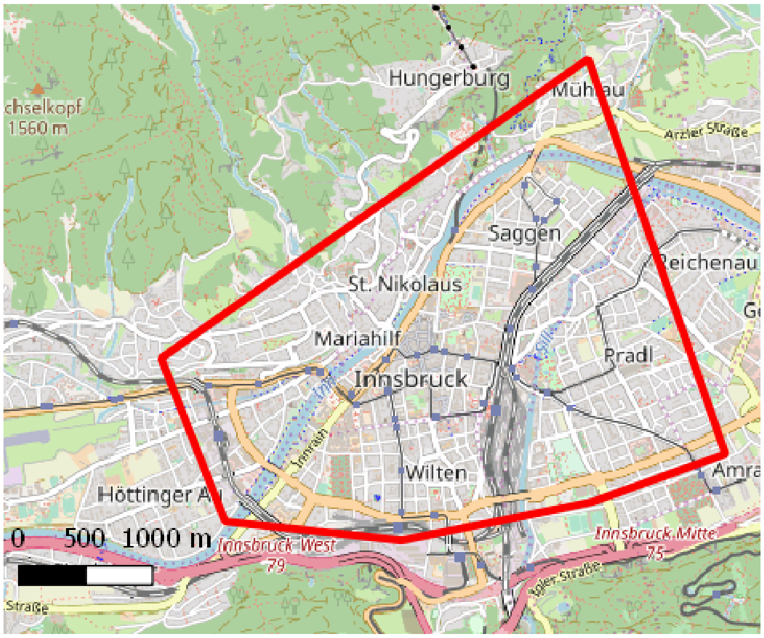

(a) Network exhibit

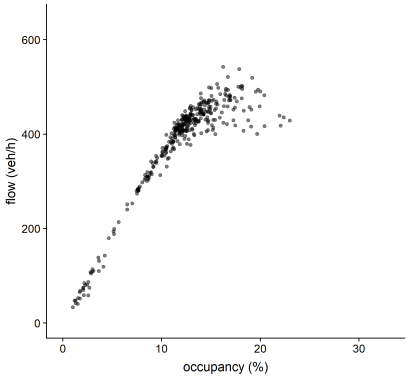

(b) Raw data

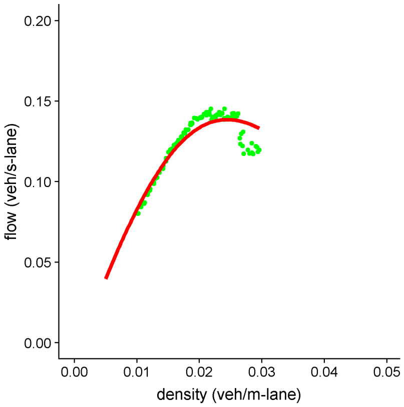

(c) Upper bound and fitted curve

Figure 21: Innsbruck Region I. (**a**) shows the network exhibit used for the MFD estimation and calculation of network topology variables. The map is oriented to the north and is obtained from OpenStreetMap. (**b**) presents the scatter plot of the raw, i.e., unfiltered, data of the network shown in **a**. This includes all malfunctioning detectors, oversampling of lanes, detectors on residential roads etc. (**c**) shows the upper-bound of the estimated MFD and the fitted curve based on the log-exp function by Ambühl et al. (2018). Flow and density are per lane. Occupancy is in the interval 0 to 1 and means the fraction of time in the observation interval a detector is covered by vehicles, i.e., it approximates density.

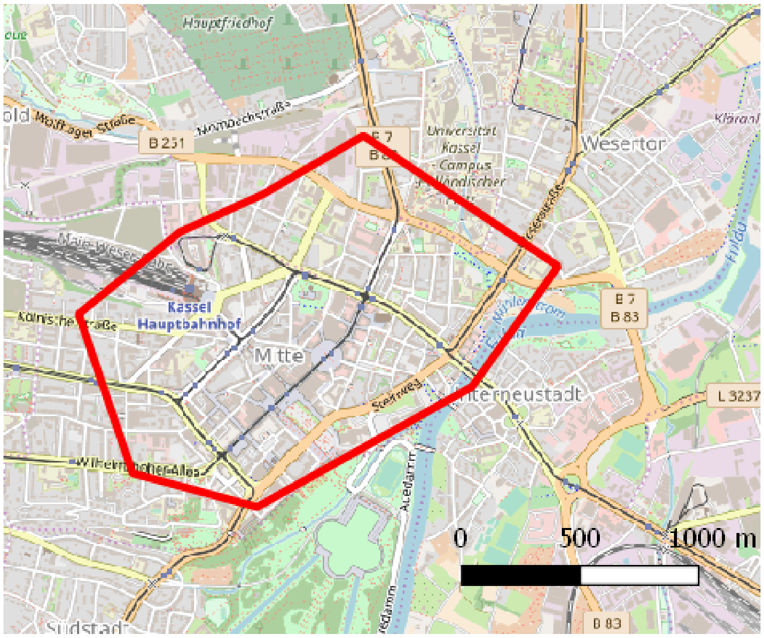

(a) Network exhibit

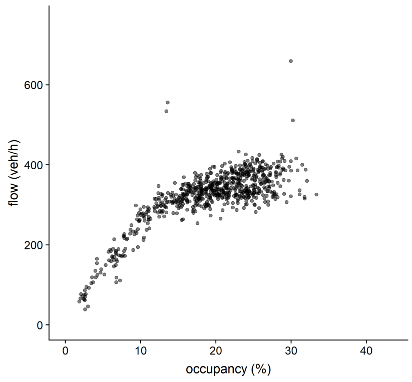

(b) Raw data

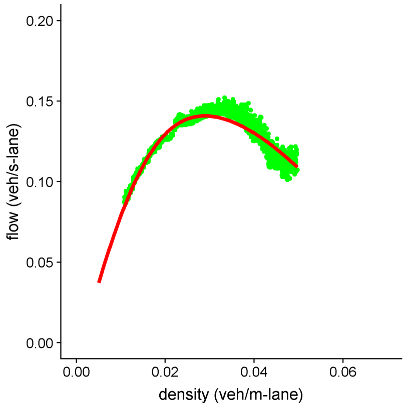

(c) Upper bound and fitted curve

Figure 22: Kassel Region I. (**a**) shows the network exhibit used for the MFD estimation and calculation of network topology variables. The map is oriented to the north and is obtained from OpenStreetMap. (**b**) presents the scatter plot of the raw, i.e., unfiltered, data of the network shown in **a**. This includes all malfunctioning detectors, oversampling of lanes, detectors on residential roads etc. (**c**) shows the upper-bound of the estimated MFD and the fitted curve based on the log-exp function by Ambühl et al. (2018). Flow and density are per lane. Occupancy is in the interval 0 to 1 and means the fraction of time in the observation interval a detector is covered by vehicles, i.e., it approximates density.

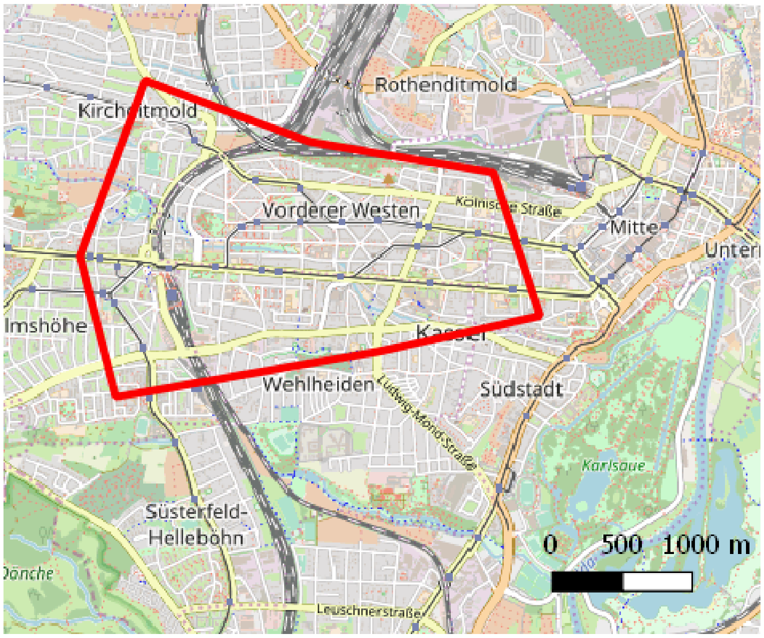

(a) Network exhibit

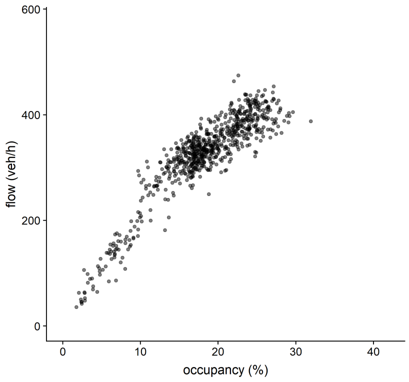

(b) Raw data

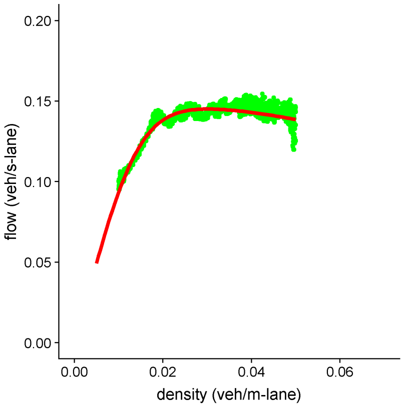

(c) Upper bound and fitted curve

Figure 23: Kassel Region II. (**a**) shows the network exhibit used for the MFD estimation and calculation of network topology variables. The map is oriented to the north and is obtained from OpenStreetMap. (**b**) presents the scatter plot of the raw, i.e., unfiltered, data of the network shown in **a**. This includes all malfunctioning detectors, oversampling of lanes, detectors on residential roads etc. (**c**) shows the upper-bound of the estimated MFD and the fitted curve based on the log-exp function by Ambühl et al. (2018). Flow and density are per lane. Occupancy is in the interval 0 to 1 and means the fraction of time in the observation interval a detector is covered by vehicles, i.e., it approximates density.

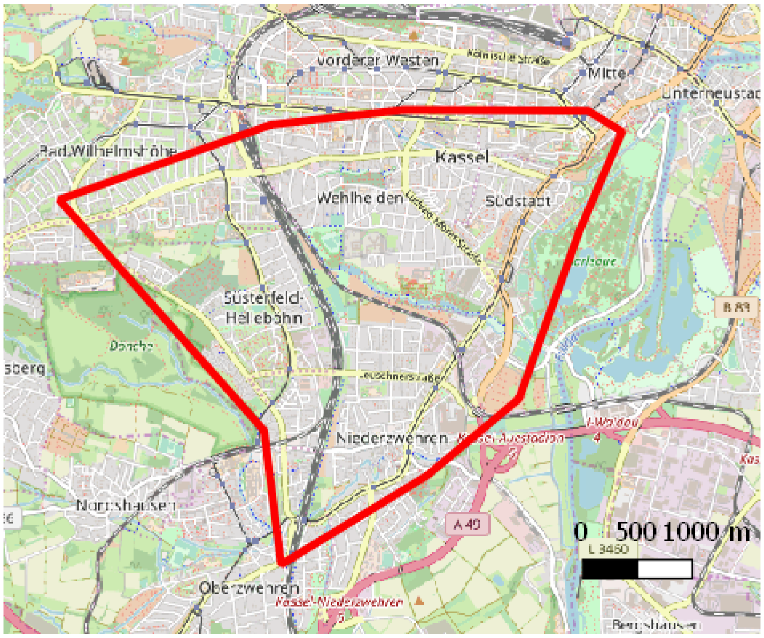

(a) Network exhibit

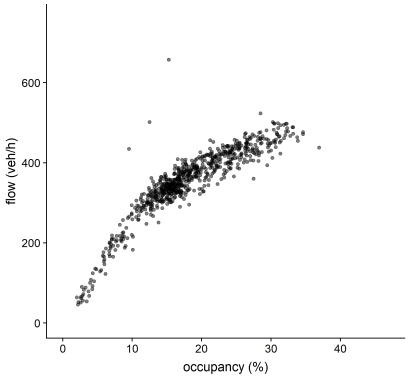

(b) Raw data

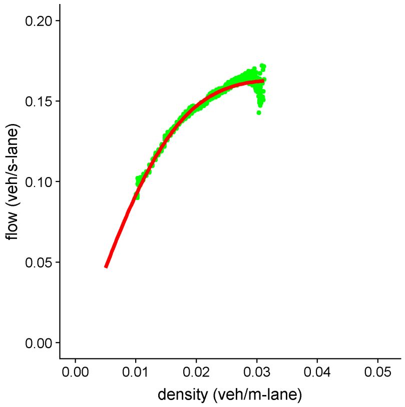

(c) Upper bound and fitted curve

Figure 24: Kassel Region III. (**a**) shows the network exhibit used for the MFD estimation and calculation of network topology variables. The map is oriented to the north and is obtained from OpenStreetMap. (**b**) presents the scatter plot of the raw, i.e., unfiltered, data of the network shown in **a**. This includes all malfunctioning detectors, oversampling of lanes, detectors on residential roads etc. (**c**) shows the upper-bound of the estimated MFD and the fitted curve based on the log-exp function by Ambühl et al. (2018). Flow and density are per lane. Occupancy is in the interval 0 to 1 and means the fraction of time in the observation interval a detector is covered by vehicles, i.e., it approximates density.

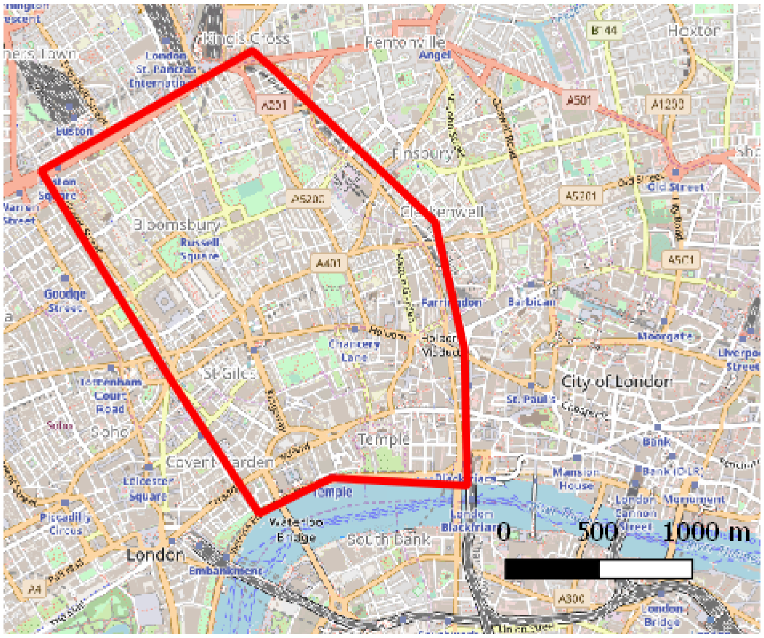

(a) Network exhibit

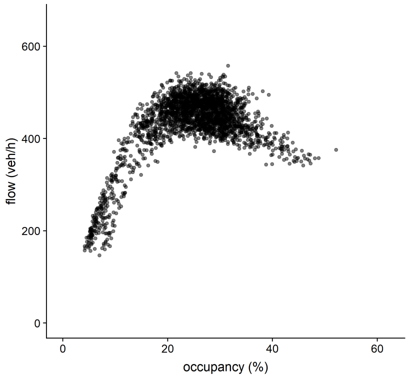

(b) Raw data

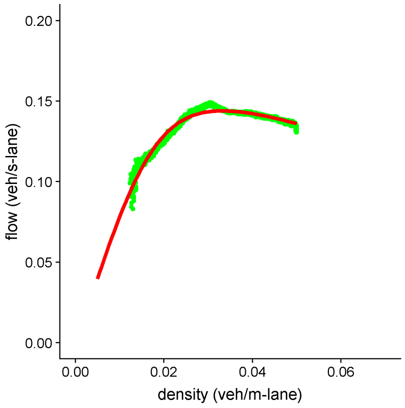

(c) Upper bound and fitted curve

Figure 25: London Region I. (**a**) shows the network exhibit used for the MFD estimation and calculation of network topology variables. The map is oriented to the north and is obtained from OpenStreetMap. (**b**) presents the scatter plot of the raw, i.e., unfiltered, data of the network shown in **a**. This includes all malfunctioning detectors, oversampling of lanes, detectors on residential roads etc. (**c**) shows the upper-bound of the estimated MFD and the fitted curve based on the log-exp function by Ambühl et al. (2018). Flow and density are per lane. Occupancy is in the interval 0 to 1 and means the fraction of time in the observation interval a detector is covered by vehicles, i.e., it approximates density.

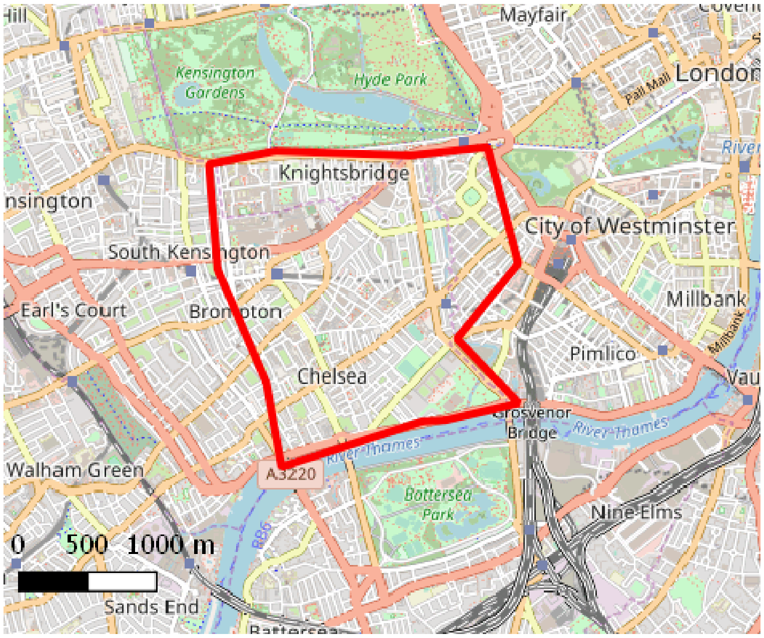

(a) Network exhibit

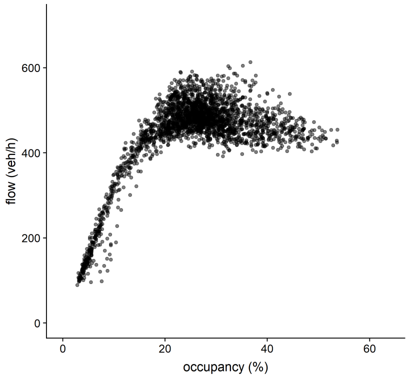

(b) Raw data

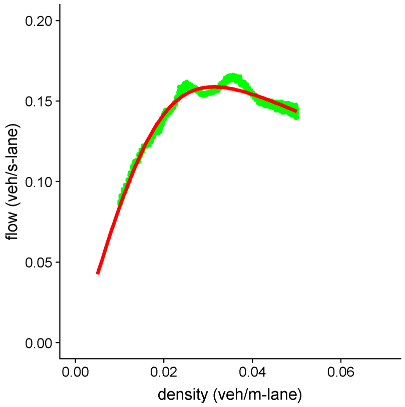

(c) Upper bound and fitted curve

Figure 26: London Region II. (**a**) shows the network exhibit used for the MFD estimation and calculation of network topology variables. The map is oriented to the north and is obtained from OpenStreetMap. (**b**) presents the scatter plot of the raw, i.e., unfiltered, data of the network shown in **a**. This includes all malfunctioning detectors, oversampling of lanes, detectors on residential roads etc. (**c**) shows the upper-bound of the estimated MFD and the fitted curve based on the log-exp function by Ambühl et al. (2018). Flow and density are per lane. Occupancy is in the interval 0 to 1 and means the fraction of time in the observation interval a detector is covered by vehicles, i.e., it approximates density.

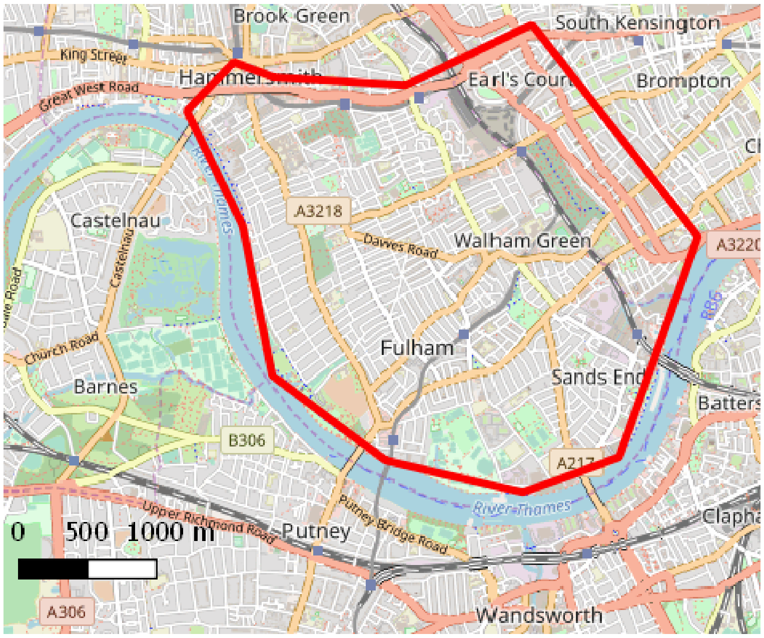

(a) Network exhibit

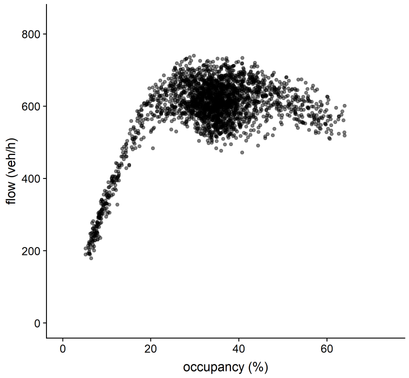

(b) Raw data

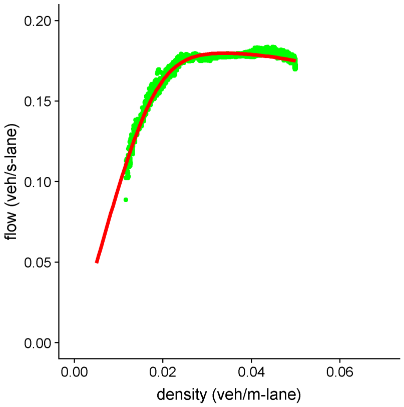

(c) Upper bound and fitted curve

Figure 27: London Region II. (**a**) shows the network exhibit used for the MFD estimation and calculation of network topology variables. The map is oriented to the north and is obtained from OpenStreetMap. (**b**) presents the scatter plot of the raw, i.e., unfiltered, data of the network shown in **a**. This includes all malfunctioning detectors, oversampling of lanes, detectors on residential roads etc. (**c**) shows the upper-bound of the estimated MFD and the fitted curve based on the log-exp function by Ambühl et al. (2018). Flow and density are per lane. Occupancy is in the interval 0 to 1 and means the fraction of time in the observation interval a detector is covered by vehicles, i.e., it approximates density.

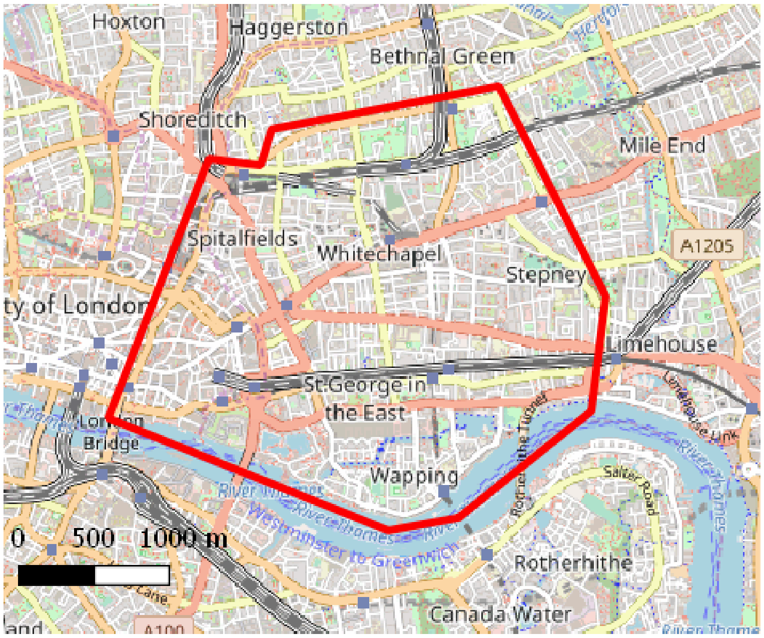

(a) Network exhibit

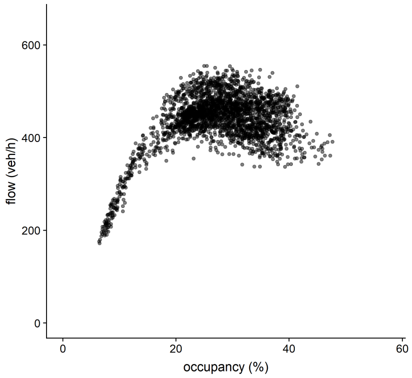

(b) Raw data

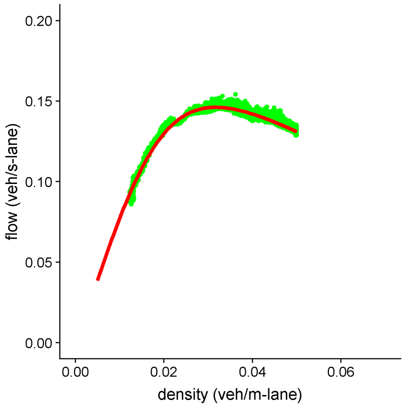

(c) Upper bound and fitted curve

Figure 28: London Region III. (**a**) shows the network exhibit used for the MFD estimation and calculation of network topology variables. The map is oriented to the north and is obtained from OpenStreetMap. (**b**) presents the scatter plot of the raw, i.e., unfiltered, data of the network shown in **a**. This includes all malfunctioning detectors, oversampling of lanes, detectors on residential roads etc. (**c**) shows the upper-bound of the estimated MFD and the fitted curve based on the log-exp function by Ambühl et al. (2018). Flow and density are per lane. Occupancy is in the interval 0 to 1 and means the fraction of time in the observation interval a detector is covered by vehicles, i.e., it approximates density.

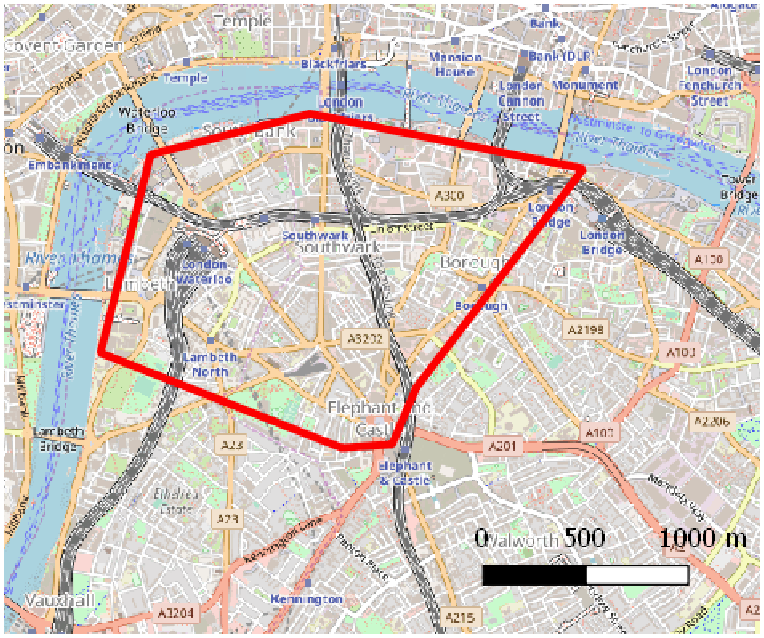

(a) Network exhibit

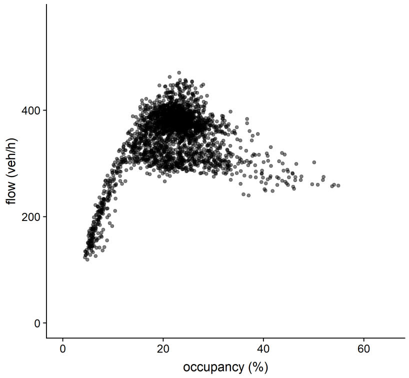

(b) Raw data

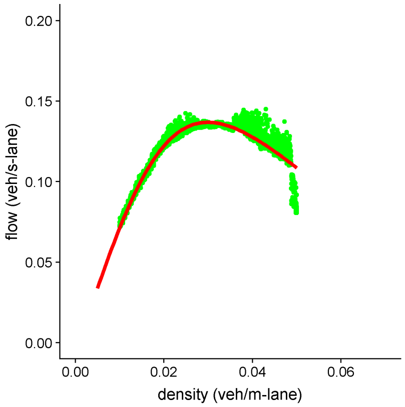

(c) Upper bound and fitted curve

Figure 29: London Region IV. (**a**) shows the network exhibit used for the MFD estimation and calculation of network topology variables. The map is oriented to the north and is obtained from OpenStreetMap. (**b**) presents the scatter plot of the raw, i.e., unfiltered, data of the network shown in **a**. This includes all malfunctioning detectors, oversampling of lanes, detectors on residential roads etc. (**c**) shows the upper-bound of the estimated MFD and the fitted curve based on the log-exp function by Ambühl et al. (2018). Flow and density are per lane. Occupancy is in the interval 0 to 1 and means the fraction of time in the observation interval a detector is covered by vehicles, i.e., it approximates density.

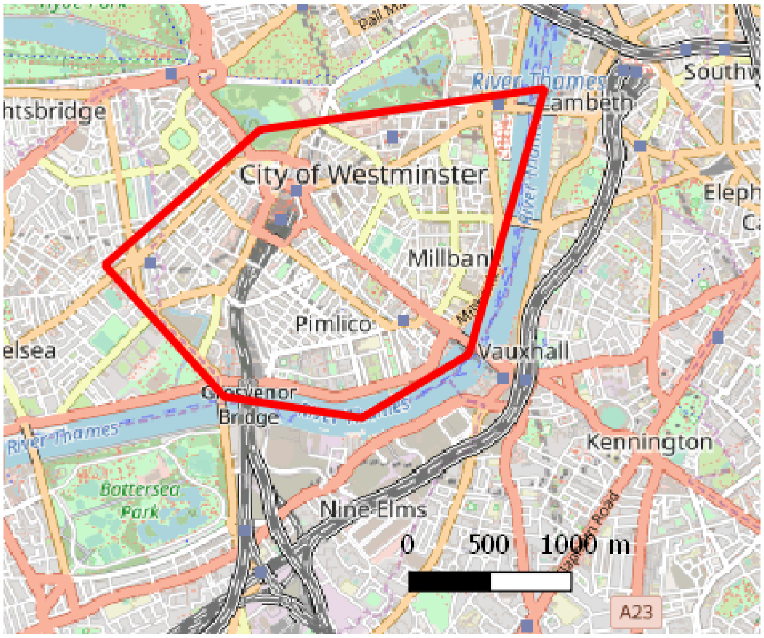

(a) Network exhibit

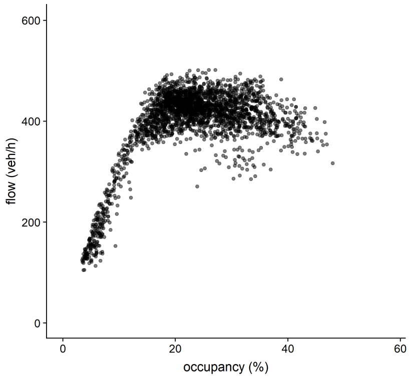

(b) Raw data

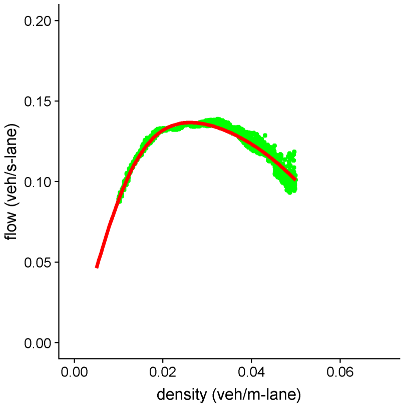

(c) Upper bound and fitted curve

Figure 30: London Region V. (**a**) shows the network exhibit used for the MFD estimation and calculation of network topology variables. The map is oriented to the north and is obtained from OpenStreetMap. (**b**) presents the scatter plot of the raw, i.e., unfiltered, data of the network shown in **a**. This includes all malfunctioning detectors, oversampling of lanes, detectors on residential roads etc. (**c**) shows the upper-bound of the estimated MFD and the fitted curve based on the log-exp function by Ambühl et al. (2018). Flow and density are per lane. Occupancy is in the interval 0 to 1 and means the fraction of time in the observation interval a detector is covered by vehicles, i.e., it approximates density.

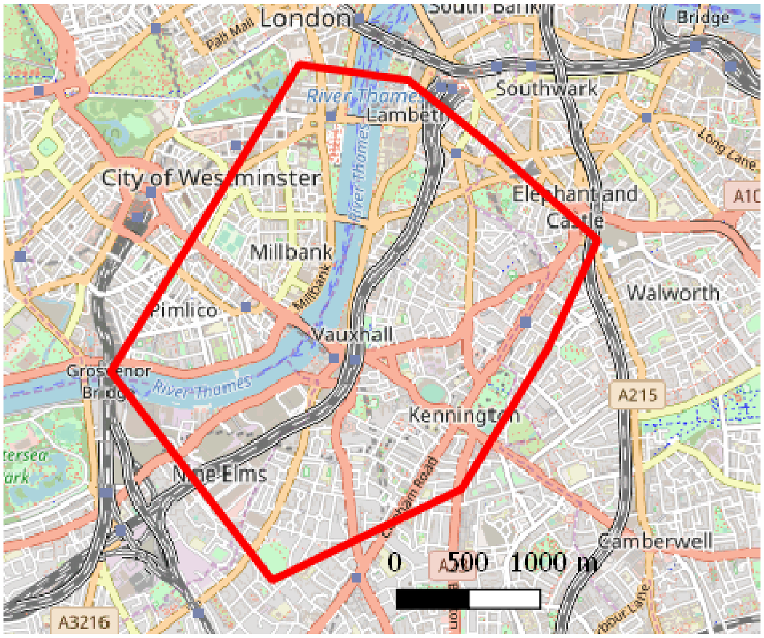

(a) Network exhibit

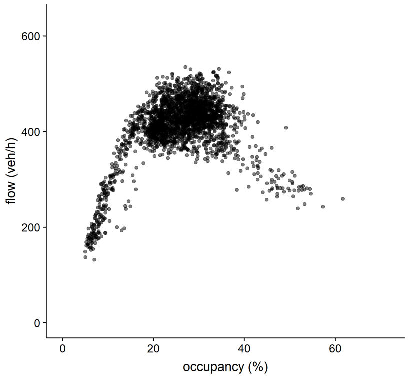

(b) Raw data

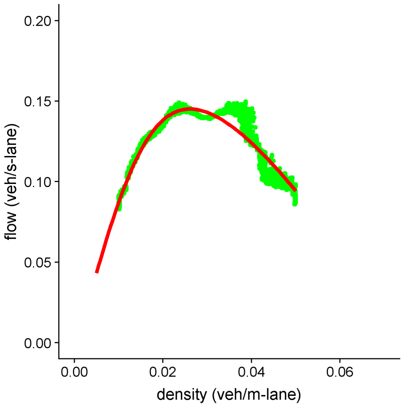

(c) Upper bound and fitted curve

Figure 31: London Region VI. (**a**) shows the network exhibit used for the MFD estimation and calculation of network topology variables. The map is oriented to the north and is obtained from OpenStreetMap. (**b**) presents the scatter plot of the raw, i.e., unfiltered, data of the network shown in **a**. This includes all malfunctioning detectors, oversampling of lanes, detectors on residential roads etc. (**c**) shows the upper-bound of the estimated MFD and the fitted curve based on the log-exp function by Ambühl et al. (2018). Flow and density are per lane. Occupancy is in the interval 0 to 1 and means the fraction of time in the observation interval a detector is covered by vehicles, i.e., it approximates density.

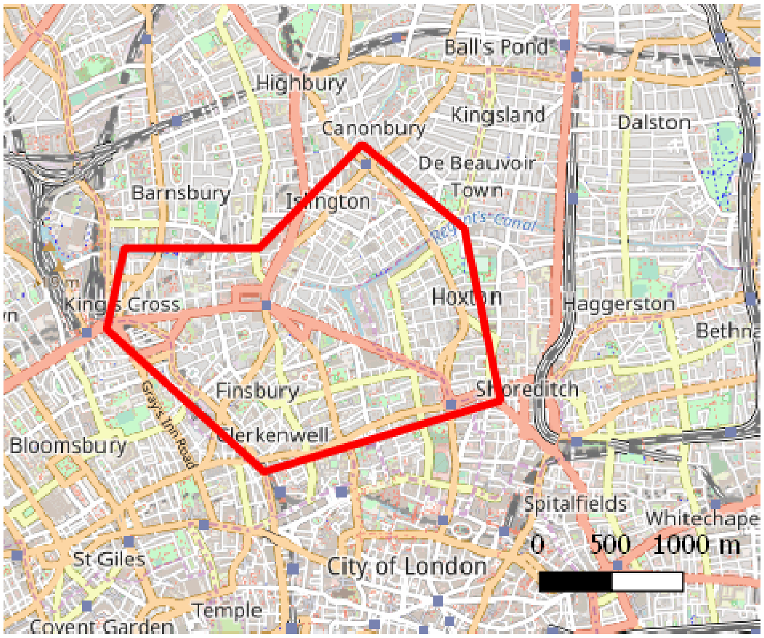

(a) Network exhibit

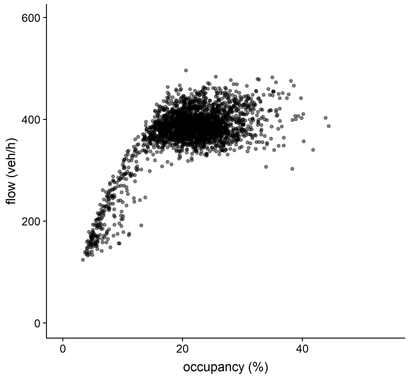

(b) Raw data

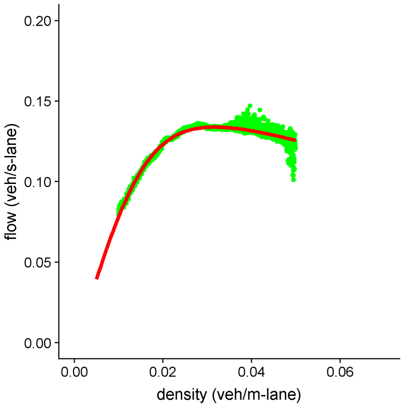

(c) Upper bound and fitted curve

Figure 32: London Region VII. (**a**) shows the network exhibit used for the MFD estimation and calculation of network topology variables. The map is oriented to the north and is obtained from OpenStreetMap. (**b**) presents the scatter plot of the raw, i.e., unfiltered, data of the network shown in **a**. This includes all malfunctioning detectors, oversampling of lanes, detectors on residential roads etc. (**c**) shows the upper-bound of the estimated MFD and the fitted curve based on the log-exp function by Ambühl et al. (2018). Flow and density are per lane. Occupancy is in the interval 0 to 1 and means the fraction of time in the observation interval a detector is covered by vehicles, i.e., it approximates density.

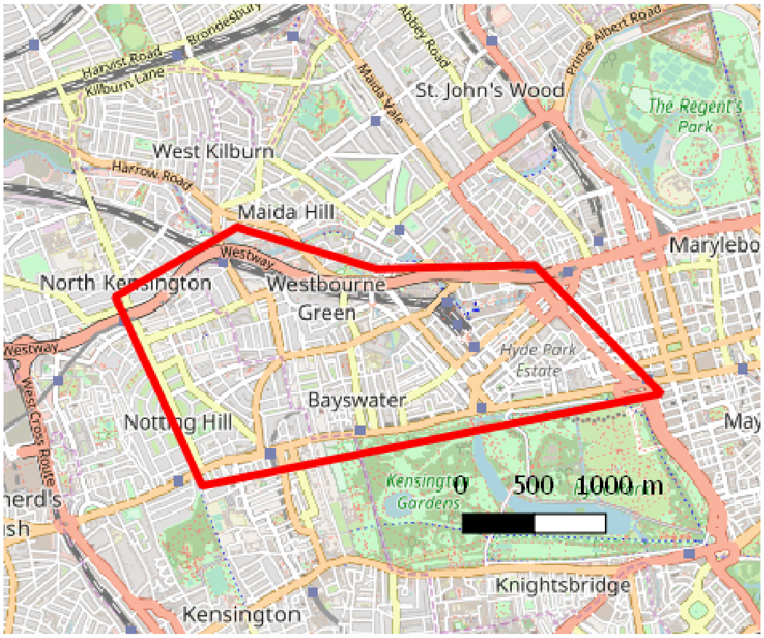

(a) Network exhibit

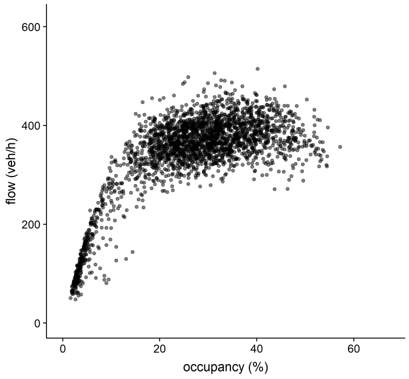

(b) Raw data

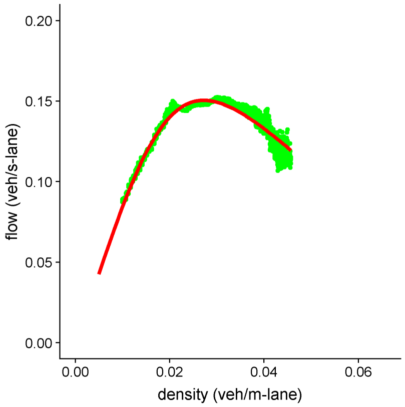

(c) Upper bound and fitted curve

Figure 33: London Region VIII. (**a**) shows the network exhibit used for the MFD estimation and calculation of network topology variables. The map is oriented to the north and is obtained from OpenStreetMap. (**b**) presents the scatter plot of the raw, i.e., unfiltered, data of the network shown in **a**. This includes all malfunctioning detectors, oversampling of lanes, detectors on residential roads etc. (**c**) shows the upper-bound of the estimated MFD and the fitted curve based on the log-exp function by Ambühl et al. (2018). Flow and density are per lane. Occupancy is in the interval 0 to 1 and means the fraction of time in the observation interval a detector is covered by vehicles, i.e., it approximates density.

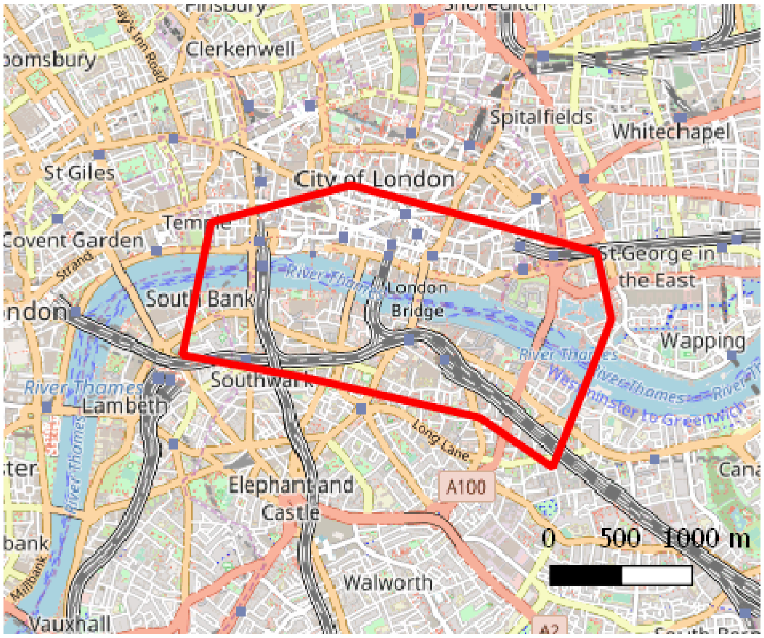

(a) Network exhibit

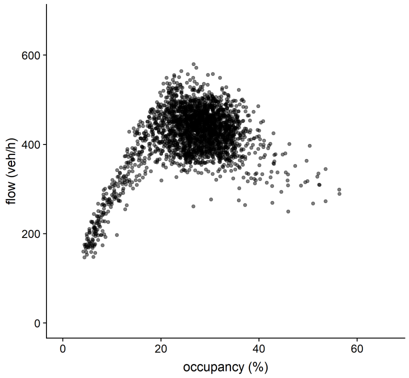

(b) Raw data

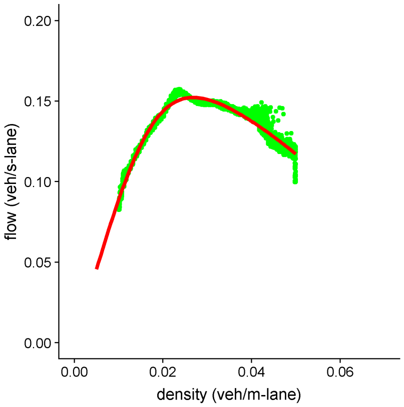

(c) Upper bound and fitted curve

Figure 34: London Region IX. (**a**) shows the network exhibit used for the MFD estimation and calculation of network topology variables. The map is oriented to the north and is obtained from OpenStreetMap. (**b**) presents the scatter plot of the raw, i.e., unfiltered, data of the network shown in **a**. This includes all malfunctioning detectors, oversampling of lanes, detectors on residential roads etc. (**c**) shows the upper-bound of the estimated MFD and the fitted curve based on the log-exp function by Ambühl et al. (2018). Flow and density are per lane. Occupancy is in the interval 0 to 1 and means the fraction of time in the observation interval a detector is covered by vehicles, i.e., it approximates density.

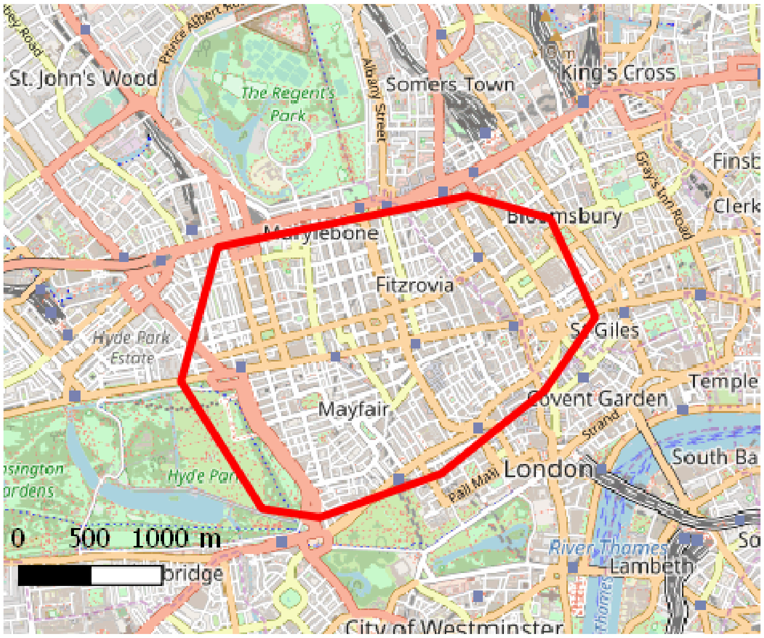

(a) Network exhibit

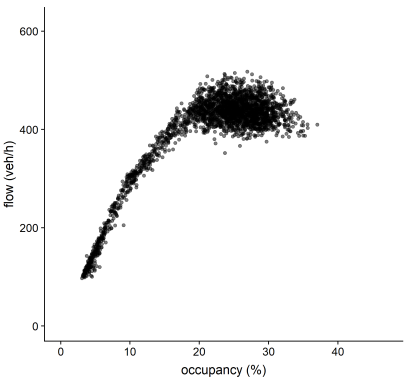

(b) Raw data

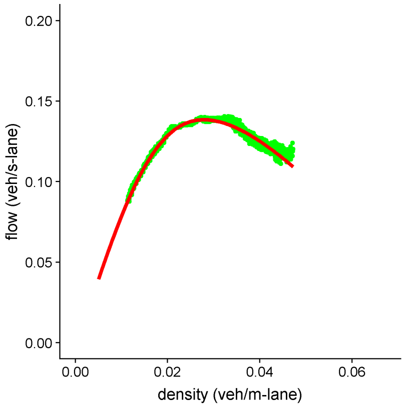

(c) Upper bound and fitted curve

Figure 35: London Region X. (**a**) shows the network exhibit used for the MFD estimation and calculation of network topology variables. The map is oriented to the north and is obtained from OpenStreetMap. (**b**) presents the scatter plot of the raw, i.e., unfiltered, data of the network shown in **a**. This includes all malfunctioning detectors, oversampling of lanes, detectors on residential roads etc. (**c**) shows the upper-bound of the estimated MFD and the fitted curve based on the log-exp function by Ambühl et al. (2018). Flow and density are per lane. Occupancy is in the interval 0 to 1 and means the fraction of time in the observation interval a detector is covered by vehicles, i.e., it approximates density.

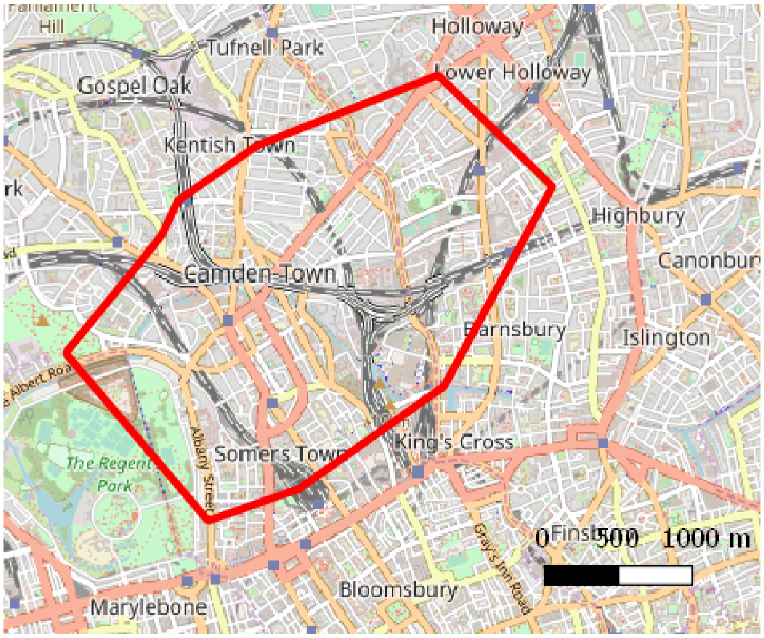

(a) Network exhibit

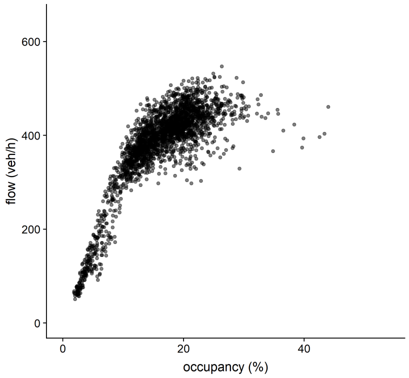

(b) Raw data

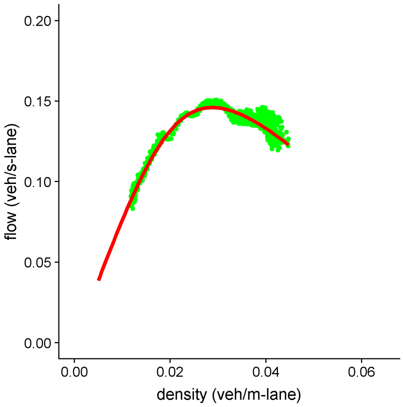

(c) Upper bound and fitted curve

Figure 36: London Region XI. (**a**) shows the network exhibit used for the MFD estimation and calculation of network topology variables. The map is oriented to the north and is obtained from OpenStreetMap. (**b**) presents the scatter plot of the raw, i.e., unfiltered, data of the network shown in **a**. This includes all malfunctioning detectors, oversampling of lanes, detectors on residential roads etc. (**c**) shows the upper-bound of the estimated MFD and the fitted curve based on the log-exp function by Ambühl et al. (2018). Flow and density are per lane. Occupancy is in the interval 0 to 1 and means the fraction of time in the observation interval a detector is covered by vehicles, i.e., it approximates density.

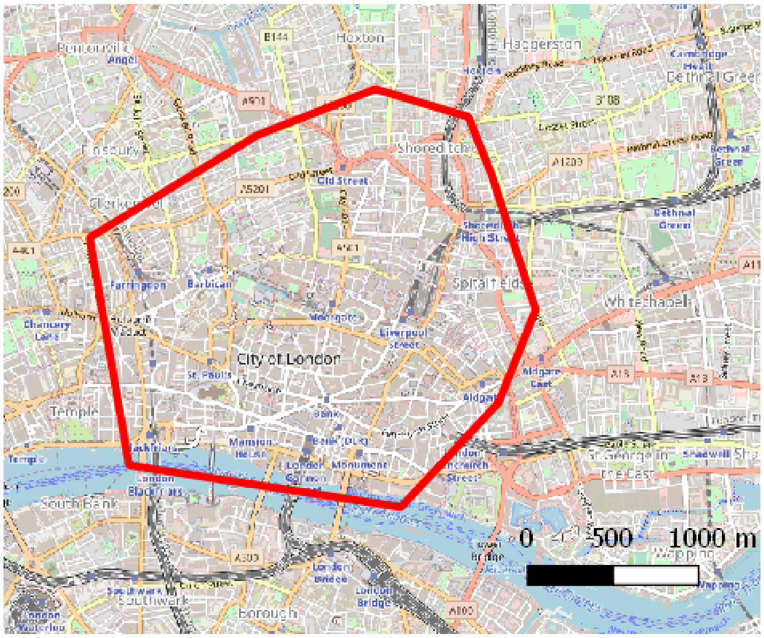

(a) Network exhibit

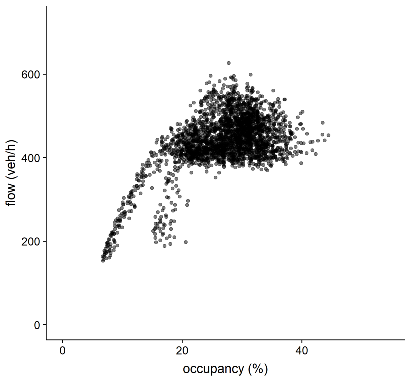

(b) Raw data

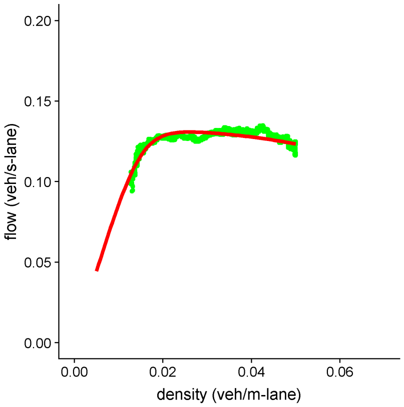

(c) Upper bound and fitted curve

Figure 37: London Region XII. (**a**) shows the network exhibit used for the MFD estimation and calculation of network topology variables. The map is oriented to the north and is obtained from OpenStreetMap. (**b**) presents the scatter plot of the raw, i.e., unfiltered, data of the network shown in **a**. This includes all malfunctioning detectors, oversampling of lanes, detectors on residential roads etc. (**c**) shows the upper-bound of the estimated MFD and the fitted curve based on the log-exp function by Ambühl et al. (2018). Flow and density are per lane. Occupancy is in the interval 0 to 1 and means the fraction of time in the observation interval a detector is covered by vehicles, i.e., it approximates density.

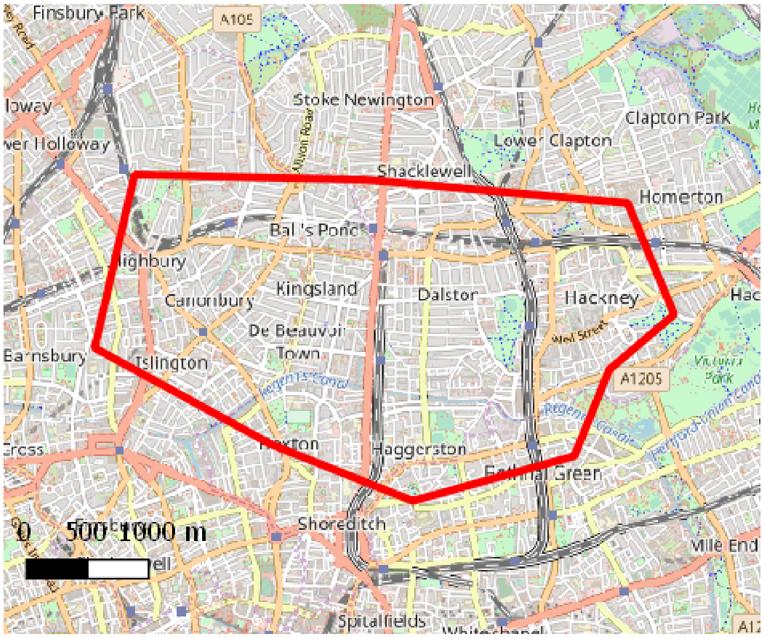

(a) Network exhibit

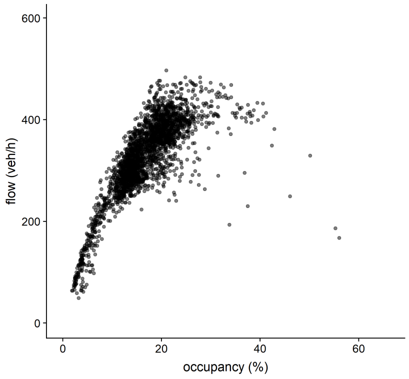

(b) Raw data

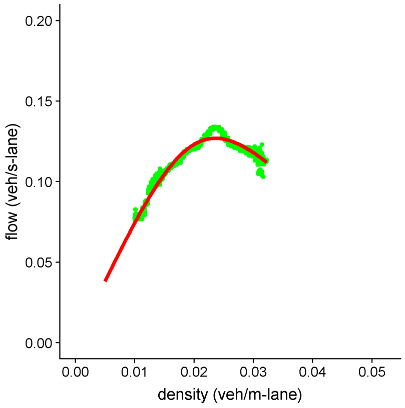

(c) Upper bound and fitted curve

Figure 38: London Region XIII. (**a**) shows the network exhibit used for the MFD estimation and calculation of network topology variables. The map is oriented to the north and is obtained from OpenStreetMap. (**b**) presents the scatter plot of the raw, i.e., unfiltered, data of the network shown in **a**. This includes all malfunctioning detectors, oversampling of lanes, detectors on residential roads etc. (**c**) shows the upper-bound of the estimated MFD and the fitted curve based on the log-exp function by Ambühl et al. (2018). Flow and density are per lane. Occupancy is in the interval 0 to 1 and means the fraction of time in the observation interval a detector is covered by vehicles, i.e., it approximates density.

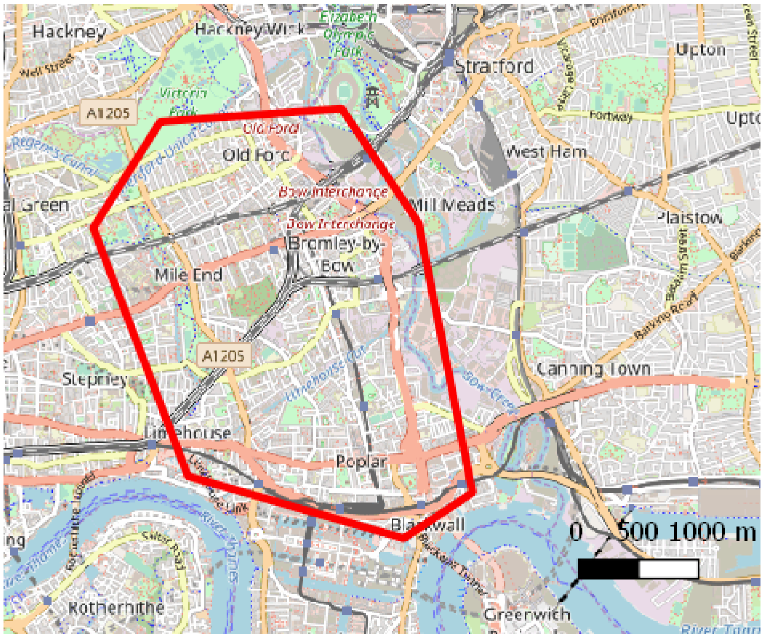

(a) Network exhibit

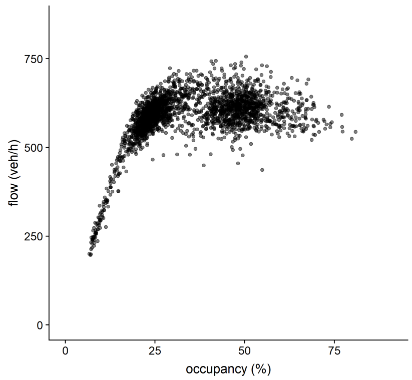

(b) Raw data

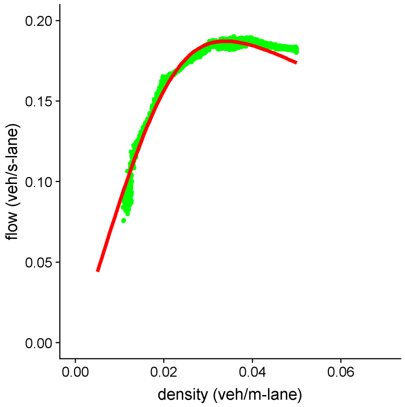

(c) Upper bound and fitted curve

Figure 39: London Region XIV. (**a**) shows the network exhibit used for the MFD estimation and calculation of network topology variables. The map is oriented to the north and is obtained from OpenStreetMap. (**b**) presents the scatter plot of the raw, i.e., unfiltered, data of the network shown in **a**. This includes all malfunctioning detectors, oversampling of lanes, detectors on residential roads etc. (**c**) shows the upper-bound of the estimated MFD and the fitted curve based on the log-exp function by Ambühl et al. (2018). Flow and density are per lane. Occupancy is in the interval 0 to 1 and means the fraction of time in the observation interval a detector is covered by vehicles, i.e., it approximates density.

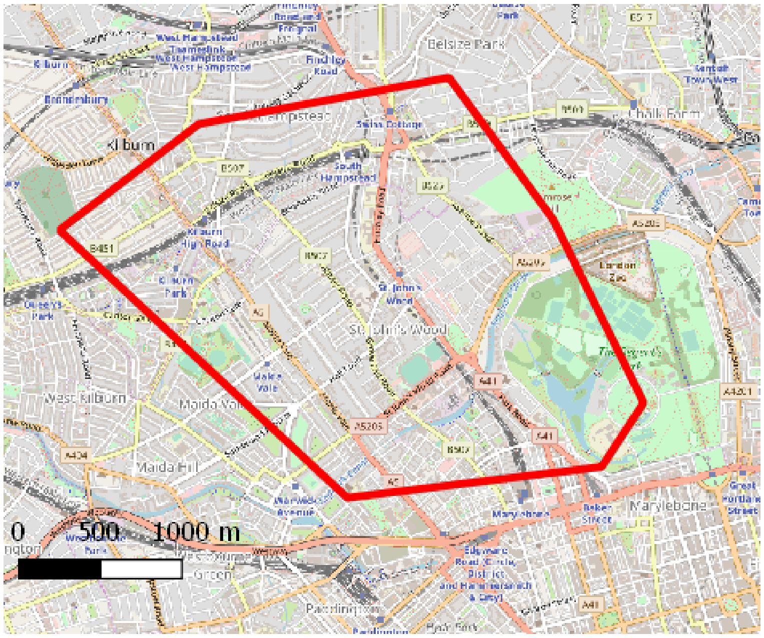

(a) Network exhibit

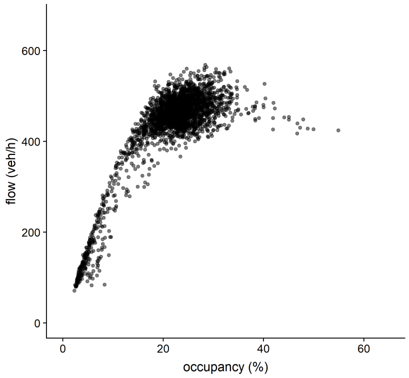

(b) Raw data

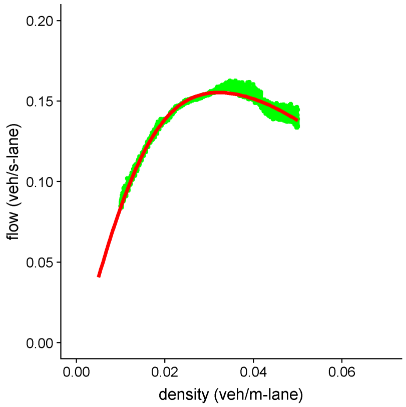

(c) Upper bound and fitted curve

Figure 40: London Region XV. (**a**) shows the network exhibit used for the MFD estimation and calculation of network topology variables. The map is oriented to the north and is obtained from OpenStreetMap. (**b**) presents the scatter plot of the raw, i.e., unfiltered, data of the network shown in **a**. This includes all malfunctioning detectors, oversampling of lanes, detectors on residential roads etc. (**c**) shows the upper-bound of the estimated MFD and the fitted curve based on the log-exp function by Ambühl et al. (2018). Flow and density are per lane. Occupancy is in the interval 0 to 1 and means the fraction of time in the observation interval a detector is covered by vehicles, i.e., it approximates density.

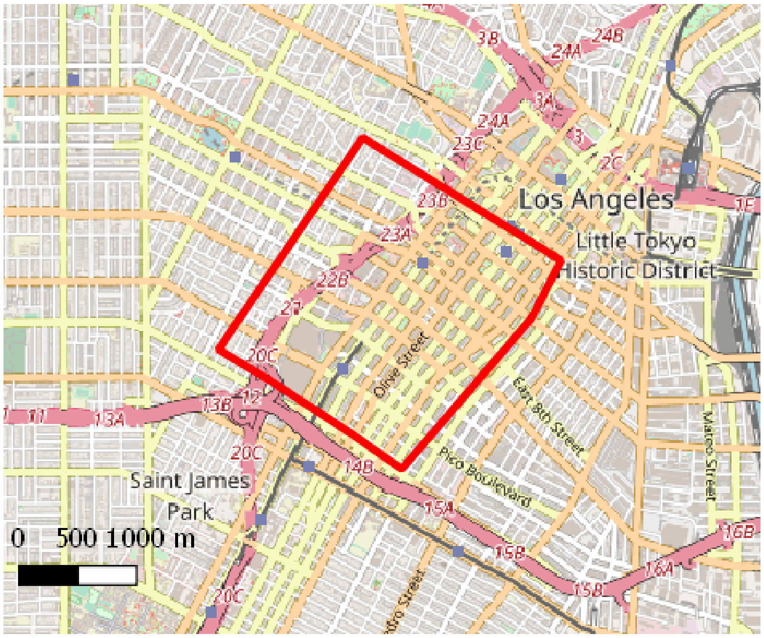

(a) Network exhibit

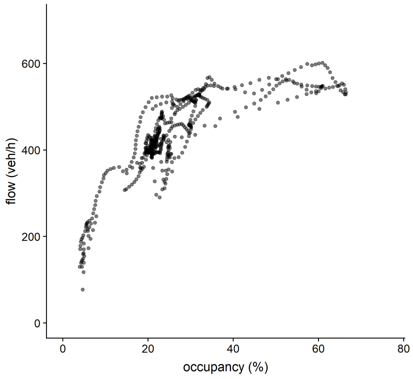

(b) Raw data

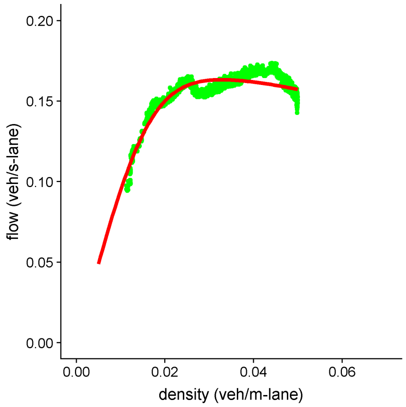

(c) Upper bound and fitted curve

Figure 41: Los Angeles Region I. (**a**) shows the network exhibit used for the MFD estimation and calculation of network topology variables. The map is oriented to the north and is obtained from OpenStreetMap. (**b**) presents the scatter plot of the raw, i.e., unfiltered, data of the network shown in **a**. This includes all malfunctioning detectors, oversampling of lanes, detectors on residential roads etc. (**c**) shows the upper-bound of the estimated MFD and the fitted curve based on the log-exp function by Ambühl et al. (2018). Flow and density are per lane. Occupancy is in the interval 0 to 1 and means the fraction of time in the observation interval a detector is covered by vehicles, i.e., it approximates density.

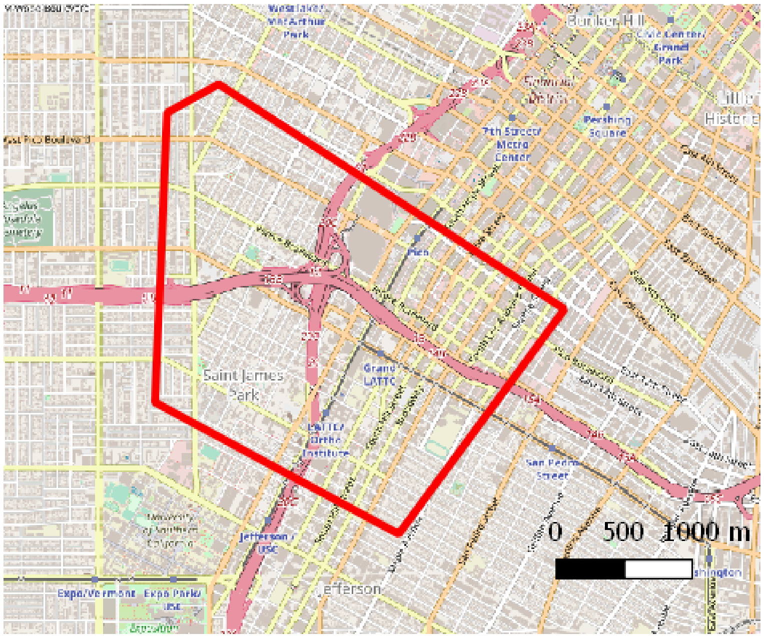

(a) Network exhibit

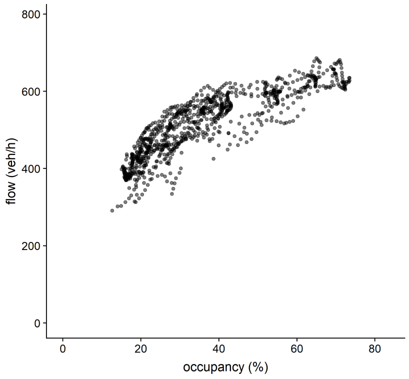

(b) Raw data

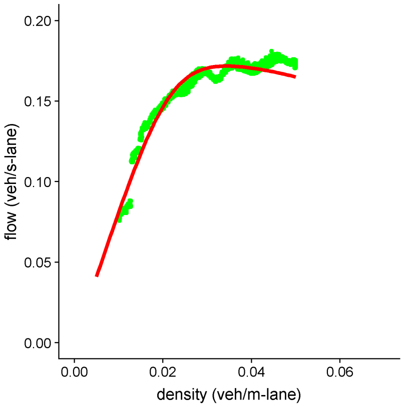

(c) Upper bound and fitted curve

Figure 42: Los Angeles Region II. (**a**) shows the network exhibit used for the MFD estimation and calculation of network topology variables. The map is oriented to the north and is obtained from OpenStreetMap. (**b**) presents the scatter plot of the raw, i.e., unfiltered, data of the network shown in **a**. This includes all malfunctioning detectors, oversampling of lanes, detectors on residential roads etc. (**c**) shows the upper-bound of the estimated MFD and the fitted curve based on the log-exp function by Ambühl et al. (2018). Flow and density are per lane. Occupancy is in the interval 0 to 1 and means the fraction of time in the observation interval a detector is covered by vehicles, i.e., it approximates density.

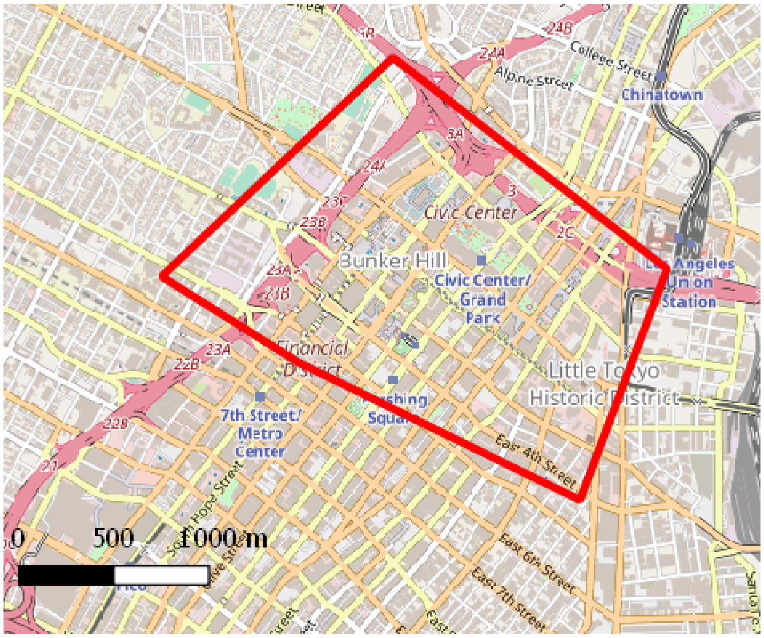

(a) Network exhibit

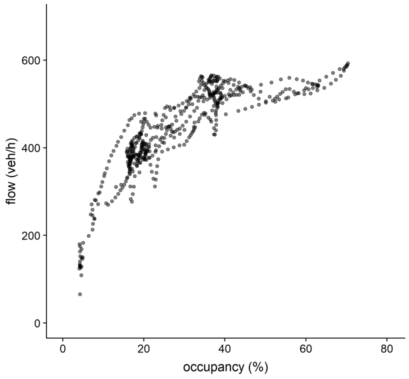

(b) Raw data

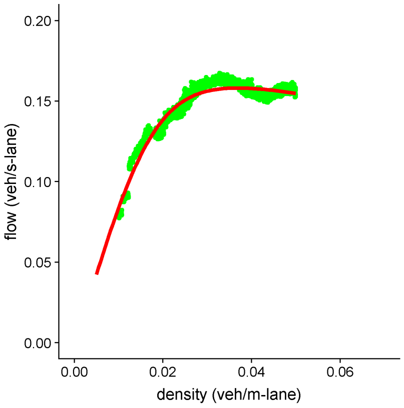

(c) Upper bound and fitted curve

Figure 43: Los Angeles Region III. (**a**) shows the network exhibit used for the MFD estimation and calculation of network topology variables. The map is oriented to the north and is obtained from OpenStreetMap. (**b**) presents the scatter plot of the raw, i.e., unfiltered, data of the network shown in **a**. This includes all malfunctioning detectors, oversampling of lanes, detectors on residential roads etc. (**c**) shows the upper-bound of the estimated MFD and the fitted curve based on the log-exp function by Ambühl et al. (2018). Flow and density are per lane. Occupancy is in the interval 0 to 1 and means the fraction of time in the observation interval a detector is covered by vehicles, i.e., it approximates density.

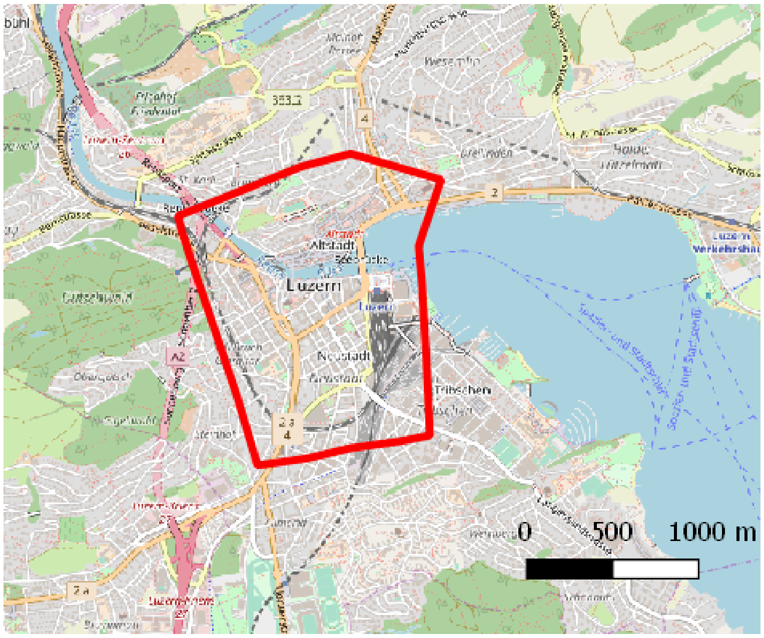

(a) Network exhibit

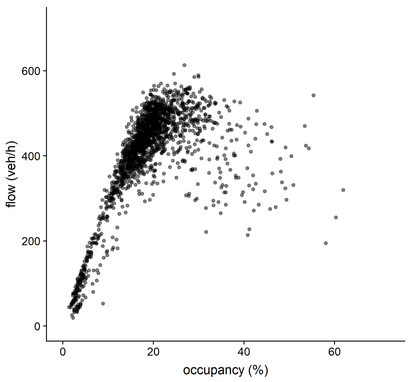

(b) Raw data

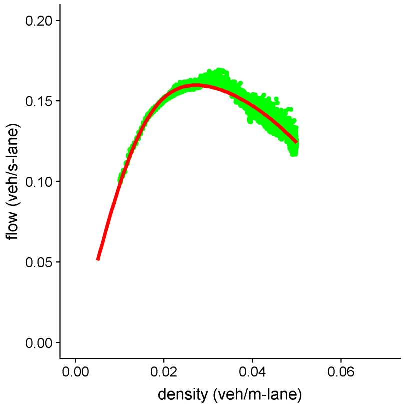

(c) Upper bound and fitted curve

Figure 44: Lucerne Region I. (**a**) shows the network exhibit used for the MFD estimation and calculation of network topology variables. The map is oriented to the north and is obtained from OpenStreetMap. (**b**) presents the scatter plot of the raw, i.e., unfiltered, data of the network shown in **a**. This includes all malfunctioning detectors, oversampling of lanes, detectors on residential roads etc. (**c**) shows the upper-bound of the estimated MFD and the fitted curve based on the log-exp function by Ambühl et al. (2018). Flow and density are per lane. Occupancy is in the interval 0 to 1 and means the fraction of time in the observation interval a detector is covered by vehicles, i.e., it approximates density.

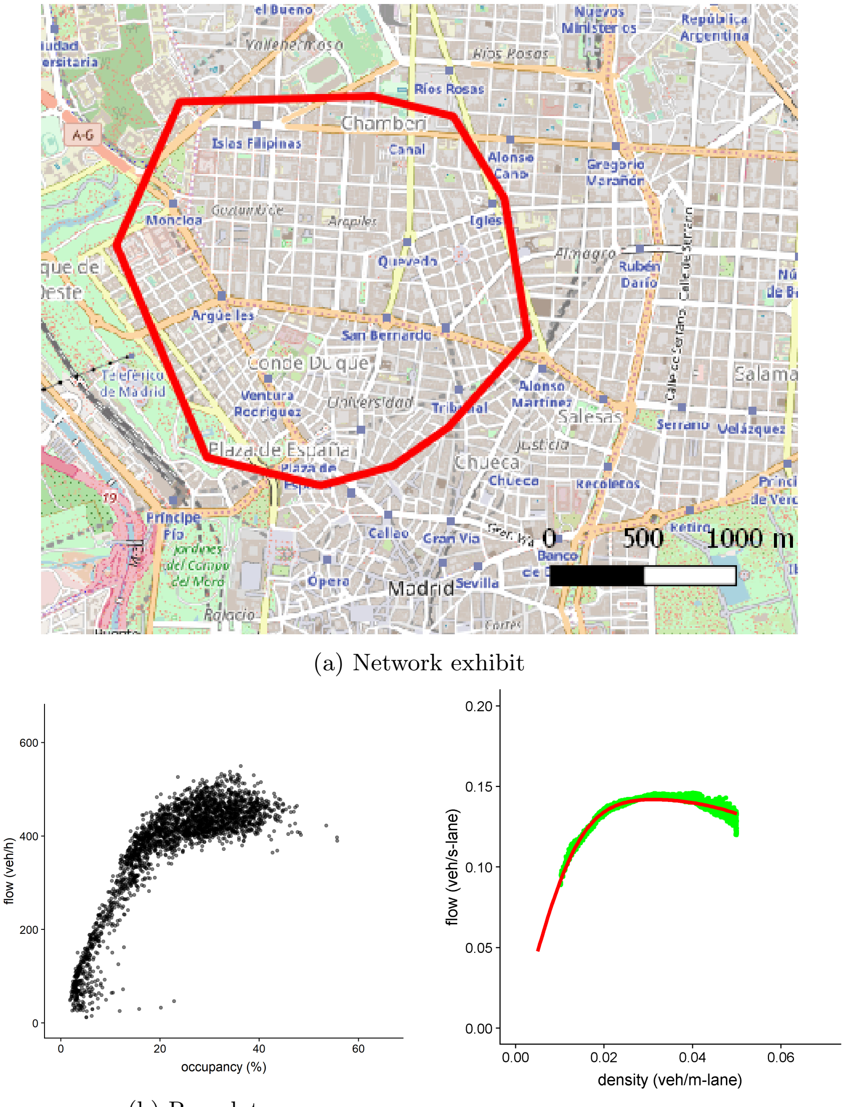

(a) Network exhibit

(b) Raw data

(c) Upper bound and fitted curve

Figure 45: Madrid Region I. (**a**) shows the network exhibit used for the MFD estimation and calculation of network topology variables. The map is oriented to the north and is obtained from OpenStreetMap. (**b**) presents the scatter plot of the raw, i.e., unfiltered, data of the network shown in **a**. This includes all malfunctioning detectors, oversampling of lanes, detectors on residential roads etc. (**c**) shows the upper-bound of the estimated MFD and the fitted curve based on the log-exp function by Ambühl et al. (2018). Flow and density are per lane. Occupancy is in the interval 0 to 1 and means the fraction of time in the observation interval a detector is covered by vehicles, i.e., it approximates density.

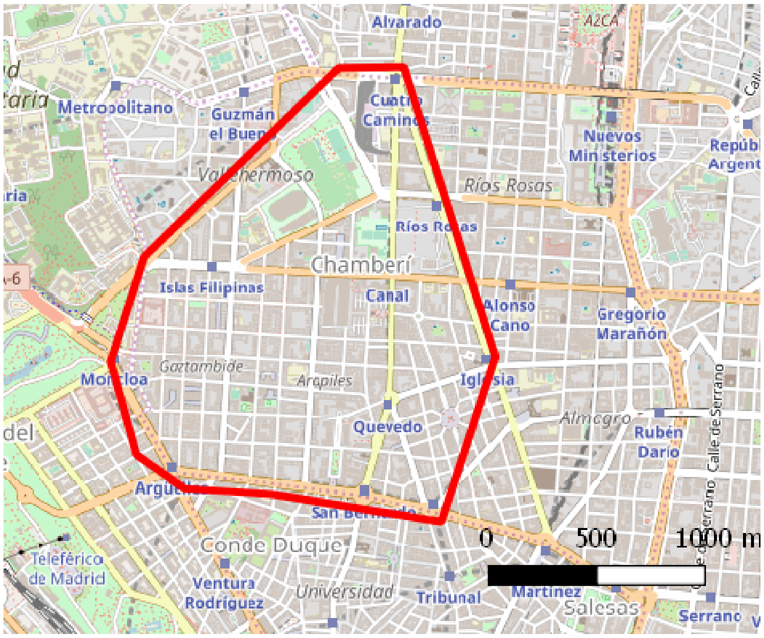

(a) Network exhibit

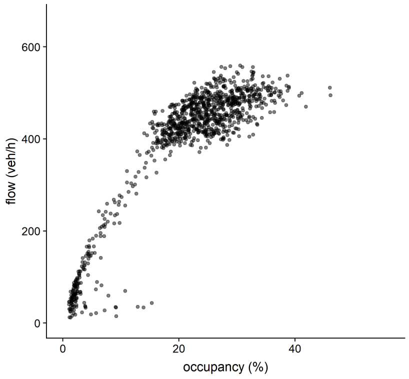

(b) Raw data

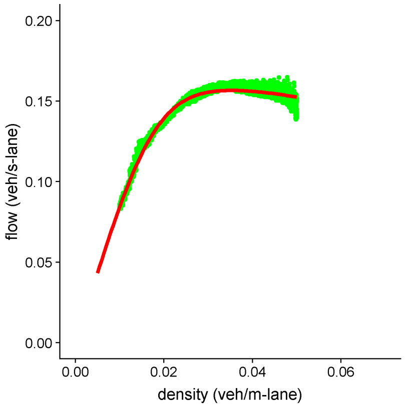

(c) Upper bound and fitted curve

Figure 46: Madrid Region II. (**a**) shows the network exhibit used for the MFD estimation and calculation of network topology variables. The map is oriented to the north and is obtained from OpenStreetMap. (**b**) presents the scatter plot of the raw, i.e., unfiltered, data of the network shown in **a**. This includes all malfunctioning detectors, oversampling of lanes, detectors on residential roads etc. (**c**) shows the upper-bound of the estimated MFD and the fitted curve based on the log-exp function by Ambühl et al. (2018). Flow and density are per lane. Occupancy is in the interval 0 to 1 and means the fraction of time in the observation interval a detector is covered by vehicles, i.e., it approximates density.

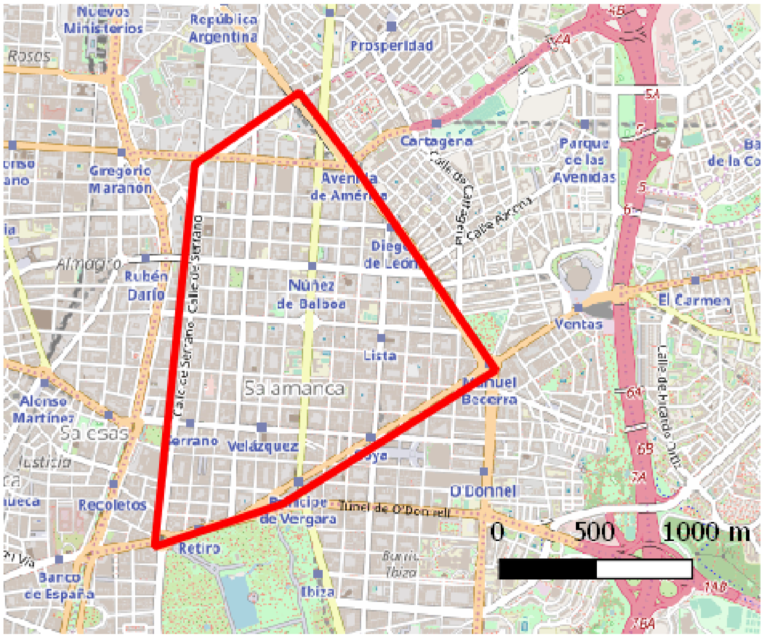

(a) Network exhibit

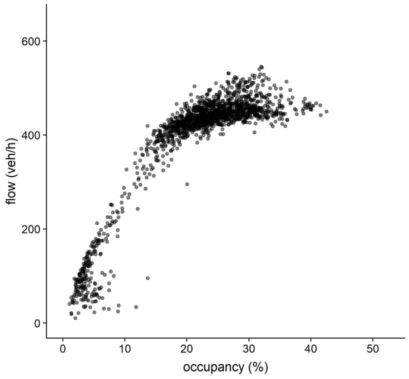

(b) Raw data

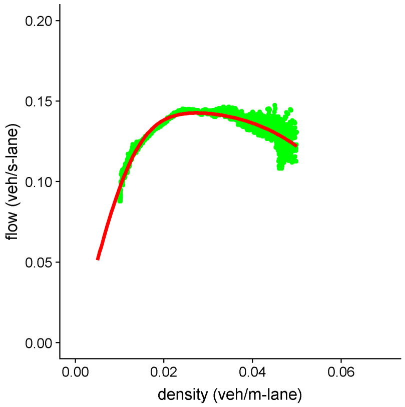

(c) Upper bound and fitted curve

Figure 47: Madrid Region III. (**a**) shows the network exhibit used for the MFD estimation and calculation of network topology variables. The map is oriented to the north and is obtained from OpenStreetMap. (**b**) presents the scatter plot of the raw, i.e., unfiltered, data of the network shown in **a**. This includes all malfunctioning detectors, oversampling of lanes, detectors on residential roads etc. (**c**) shows the upper-bound of the estimated MFD and the fitted curve based on the log-exp function by Ambühl et al. (2018). Flow and density are per lane. Occupancy is in the interval 0 to 1 and means the fraction of time in the observation interval a detector is covered by vehicles, i.e., it approximates density.

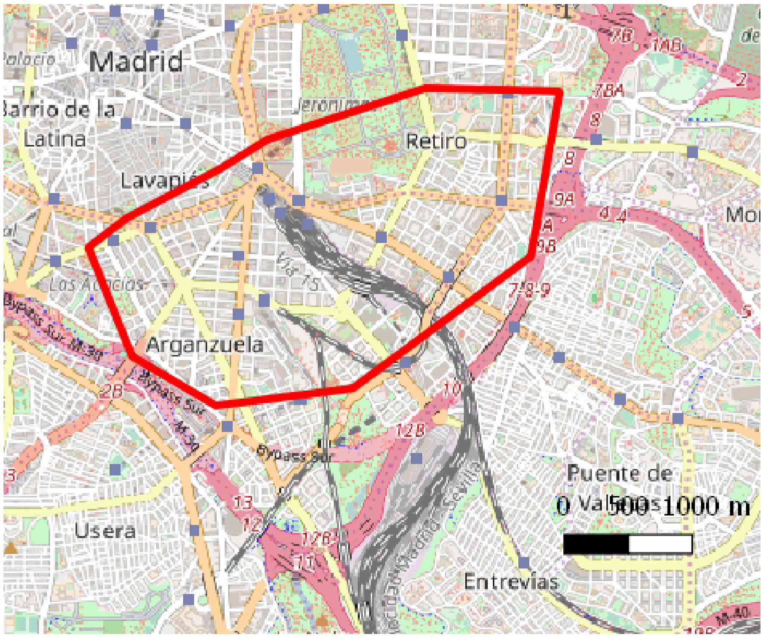

(a) Network exhibit

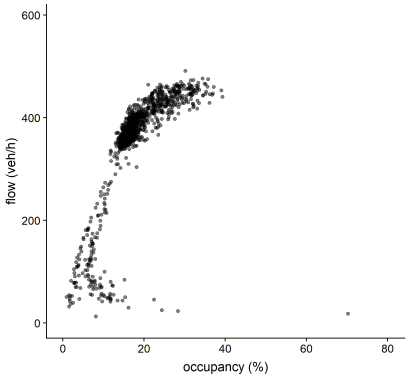

(b) Raw data

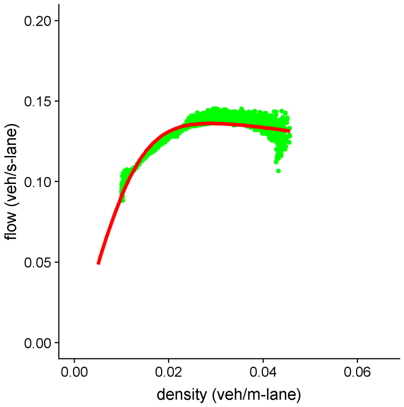

(c) Upper bound and fitted curve

Figure 48: Madrid Region IV. (**a**) shows the network exhibit used for the MFD estimation and calculation of network topology variables. The map is oriented to the north and is obtained from OpenStreetMap. (**b**) presents the scatter plot of the raw, i.e., unfiltered, data of the network shown in **a**. This includes all malfunctioning detectors, oversampling of lanes, detectors on residential roads etc. (**c**) shows the upper-bound of the estimated MFD and the fitted curve based on the log-exp function by Ambühl et al. (2018). Flow and density are per lane. Occupancy is in the interval 0 to 1 and means the fraction of time in the observation interval a detector is covered by vehicles, i.e., it approximates density.

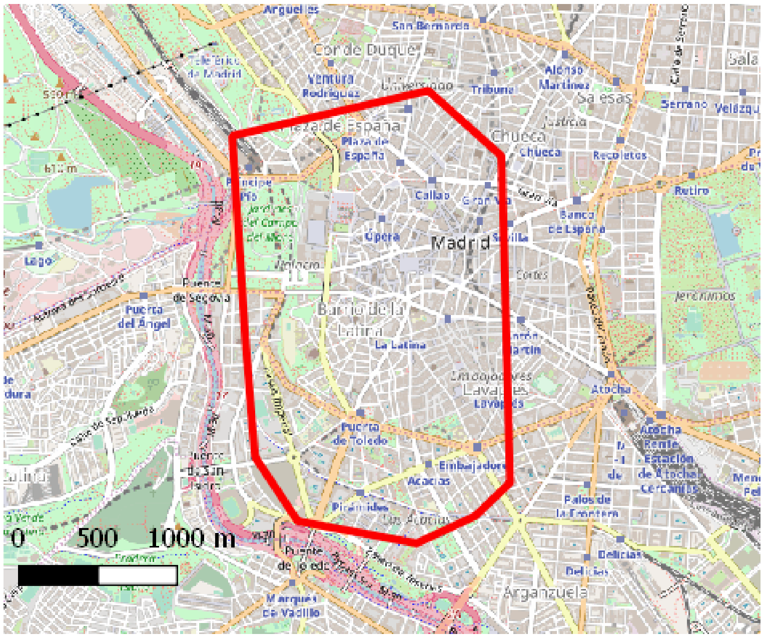

(a) Network exhibit

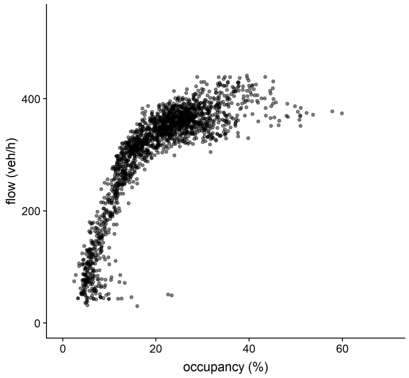

(b) Raw data

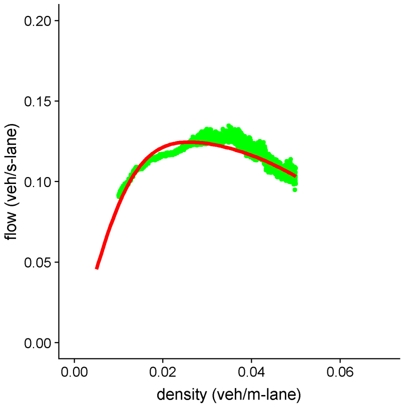

(c) Upper bound and fitted curve

Figure 49: Madrid Region V. (**a**) shows the network exhibit used for the MFD estimation and calculation of network topology variables. The map is oriented to the north and is obtained from OpenStreetMap. (**b**) presents the scatter plot of the raw, i.e., unfiltered, data of the network shown in **a**. This includes all malfunctioning detectors, oversampling of lanes, detectors on residential roads etc. (**c**) shows the upper-bound of the estimated MFD and the fitted curve based on the log-exp function by Ambühl et al. (2018). Flow and density are per lane. Occupancy is in the interval 0 to 1 and means the fraction of time in the observation interval a detector is covered by vehicles, i.e., it approximates density.

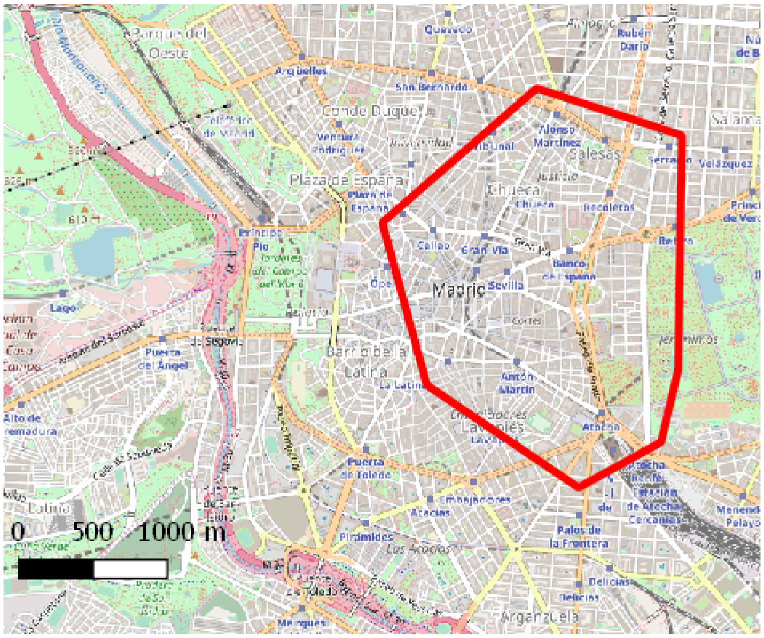

(a) Network exhibit

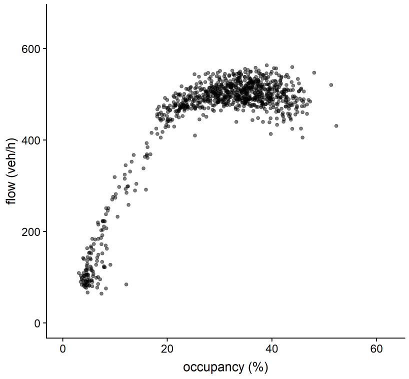

(b) Raw data

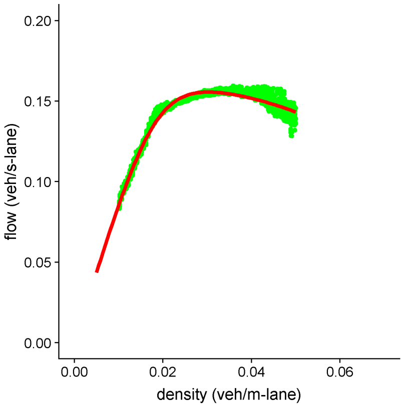

(c) Upper bound and fitted curve

Figure 50: Madrid Region VI. (**a**) shows the network exhibit used for the MFD estimation and calculation of network topology variables. The map is oriented to the north and is obtained from OpenStreetMap. (**b**) presents the scatter plot of the raw, i.e., unfiltered, data of the network shown in **a**. This includes all malfunctioning detectors, oversampling of lanes, detectors on residential roads etc. (**c**) shows the upper-bound of the estimated MFD and the fitted curve based on the log-exp function by Ambühl et al. (2018). Flow and density are per lane. Occupancy is in the interval 0 to 1 and means the fraction of time in the observation interval a detector is covered by vehicles, i.e., it approximates density.

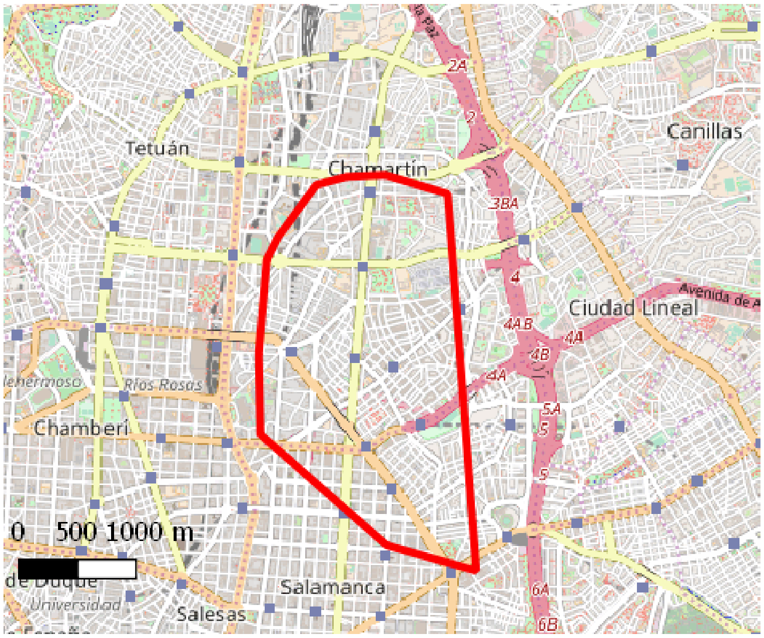

(a) Network exhibit

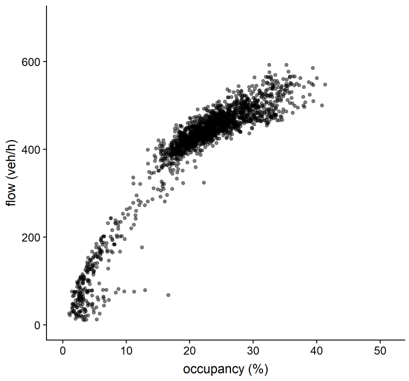

(b) Raw data

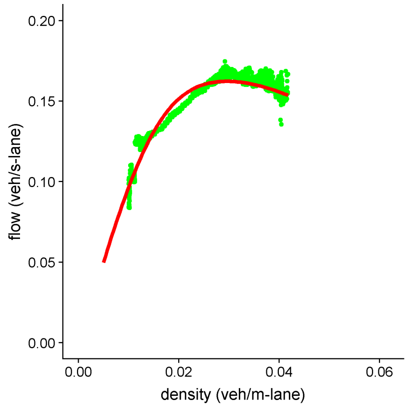

(c) Upper bound and fitted curve

Figure 51: Madrid Region VII. (**a**) shows the network exhibit used for the MFD estimation and calculation of network topology variables. The map is oriented to the north and is obtained from OpenStreetMap. (**b**) presents the scatter plot of the raw, i.e., unfiltered, data of the network shown in **a**. This includes all malfunctioning detectors, oversampling of lanes, detectors on residential roads etc. (**c**) shows the upper-bound of the estimated MFD and the fitted curve based on the log-exp function by Ambühl et al. (2018). Flow and density are per lane. Occupancy is in the interval 0 to 1 and means the fraction of time in the observation interval a detector is covered by vehicles, i.e., it approximates density.

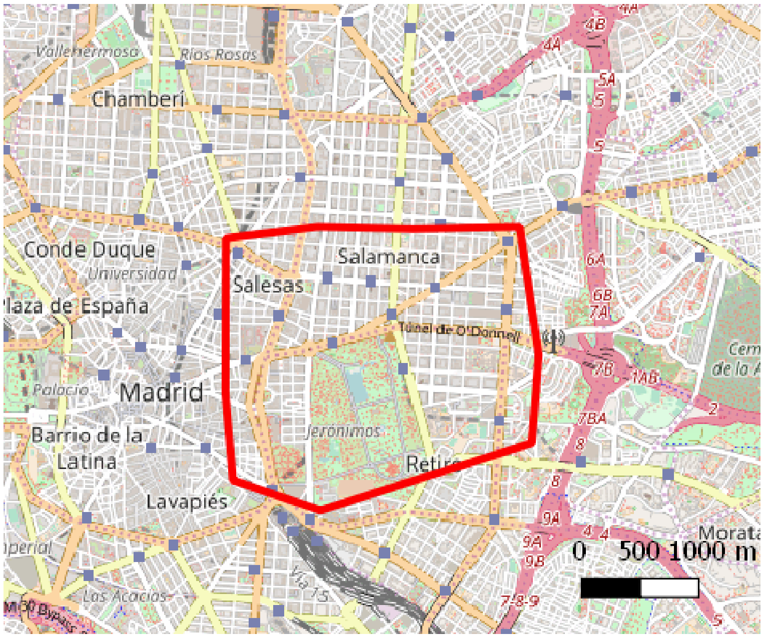

(a) Network exhibit

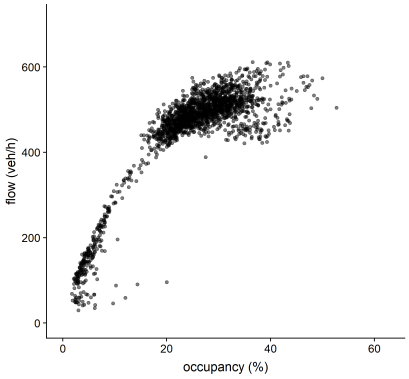

(b) Raw data

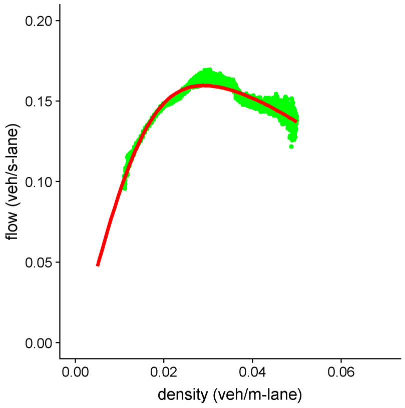

(c) Upper bound and fitted curve

Figure 52: Madrid Region VIII. (**a**) shows the network exhibit used for the MFD estimation and calculation of network topology variables. The map is oriented to the north and is obtained from OpenStreetMap. (**b**) presents the scatter plot of the raw, i.e., unfiltered, data of the network shown in **a**. This includes all malfunctioning detectors, oversampling of lanes, detectors on residential roads etc. (**c**) shows the upper-bound of the estimated MFD and the fitted curve based on the log-exp function by Ambühl et al. (2018). Flow and density are per lane. Occupancy is in the interval 0 to 1 and means the fraction of time in the observation interval a detector is covered by vehicles, i.e., it approximates density.

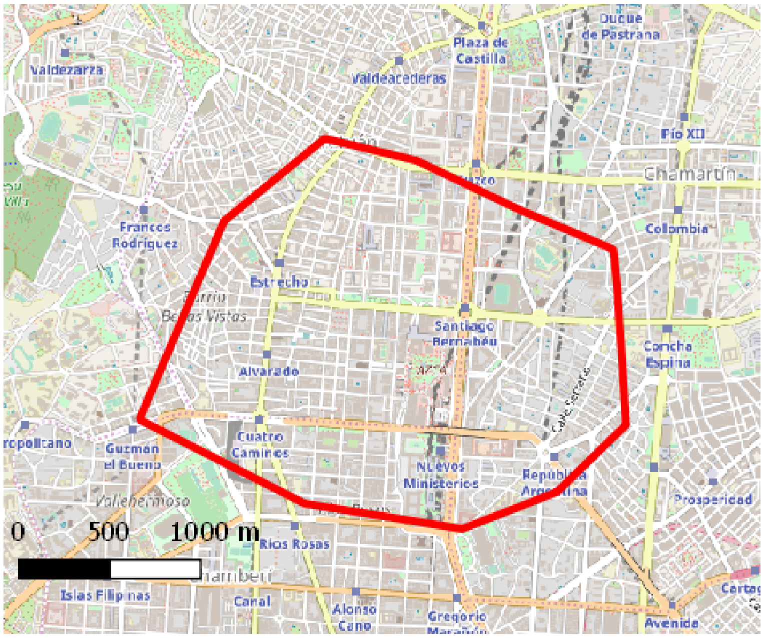

(a) Network exhibit

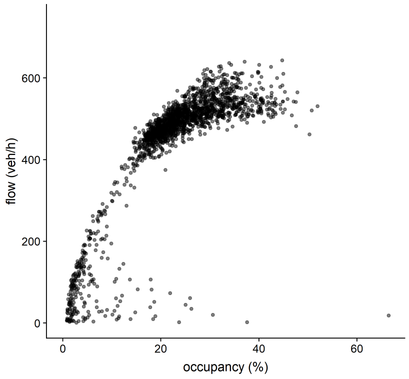

(b) Raw data

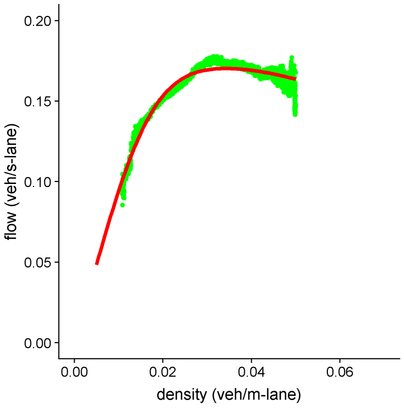

(c) Upper bound and fitted curve

Figure 53: Madrid Region IX. (**a**) shows the network exhibit used for the MFD estimation and calculation of network topology variables. The map is oriented to the north and is obtained from OpenStreetMap. (**b**) presents the scatter plot of the raw, i.e., unfiltered, data of the network shown in **a**. This includes all malfunctioning detectors, oversampling of lanes, detectors on residential roads etc. (**c**) shows the upper-bound of the estimated MFD and the fitted curve based on the log-exp function by Ambühl et al. (2018). Flow and density are per lane. Occupancy is in the interval 0 to 1 and means the fraction of time in the observation interval a detector is covered by vehicles, i.e., it approximates density.

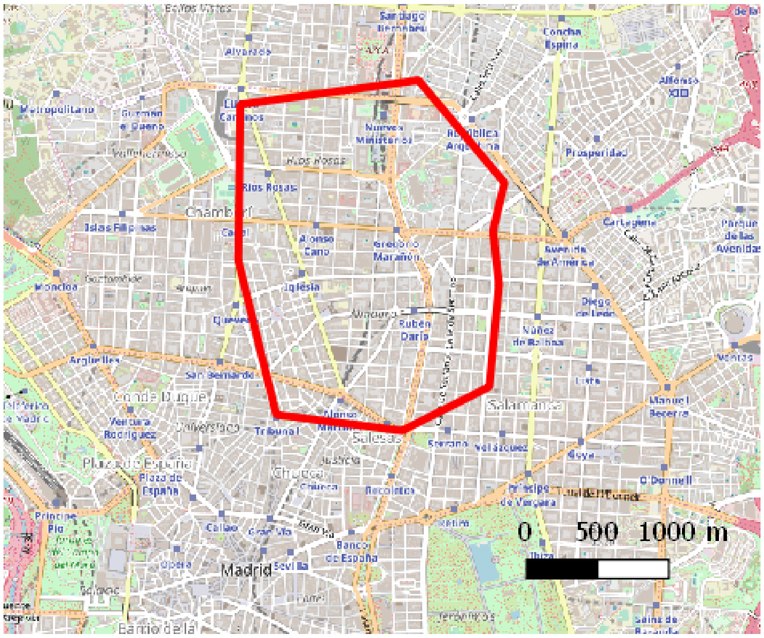

(a) Network exhibit

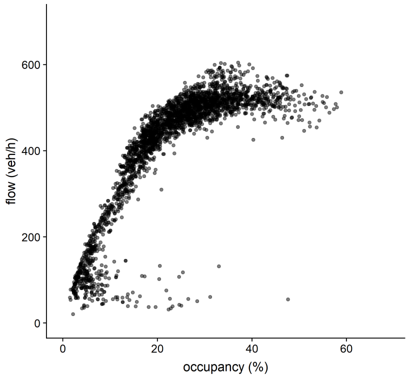

(b) Raw data

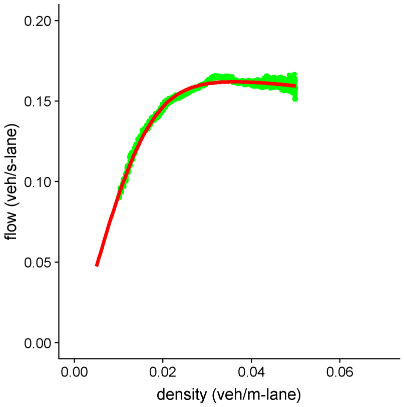

(c) Upper bound and fitted curve

Figure 54: Madrid Region X. (**a**) shows the network exhibit used for the MFD estimation and calculation of network topology variables. The map is oriented to the north and is obtained from OpenStreetMap. (**b**) presents the scatter plot of the raw, i.e., unfiltered, data of the network shown in **a**. This includes all malfunctioning detectors, oversampling of lanes, detectors on residential roads etc. (**c**) shows the upper-bound of the estimated MFD and the fitted curve based on the log-exp function by Ambühl et al. (2018). Flow and density are per lane. Occupancy is in the interval 0 to 1 and means the fraction of time in the observation interval a detector is covered by vehicles, i.e., it approximates density.

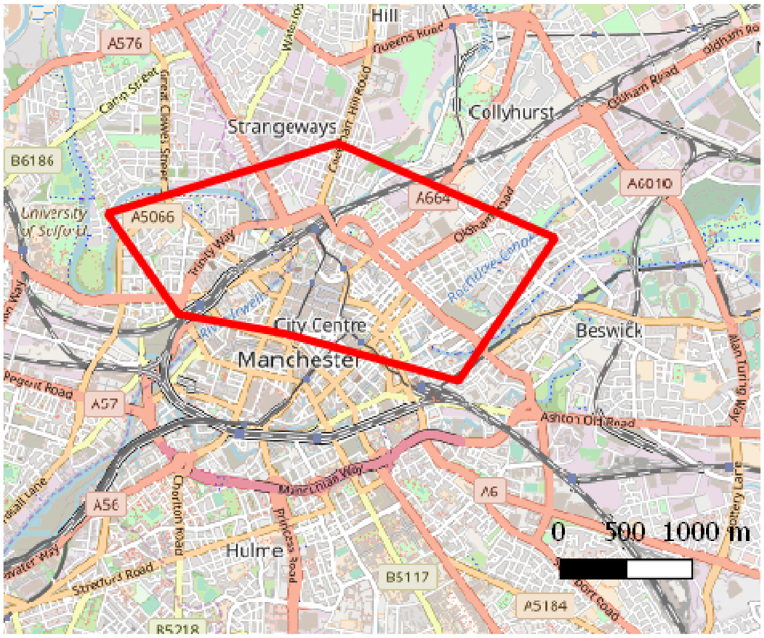

(a) Network exhibit

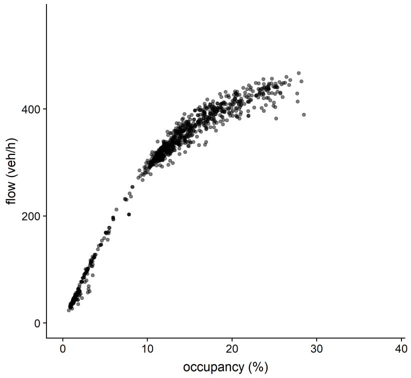

(b) Raw data

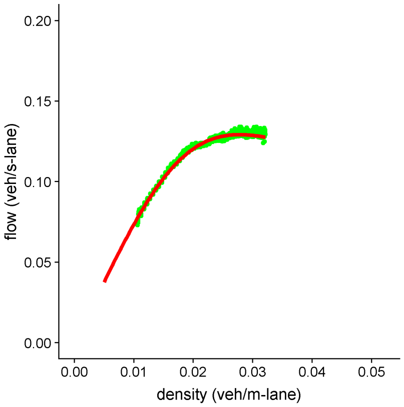

(c) Upper bound and fitted curve

Figure 55: Manchester Region I. (**a**) shows the network exhibit used for the MFD estimation and calculation of network topology variables. The map is oriented to the north and is obtained from OpenStreetMap. (**b**) presents the scatter plot of the raw, i.e., unfiltered, data of the network shown in **a**. This includes all malfunctioning detectors, oversampling of lanes, detectors on residential roads etc. (**c**) shows the upper-bound of the estimated MFD and the fitted curve based on the log-exp function by Ambühl et al. (2018). Flow and density are per lane. Occupancy is in the interval 0 to 1 and means the fraction of time in the observation interval a detector is covered by vehicles, i.e., it approximates density.

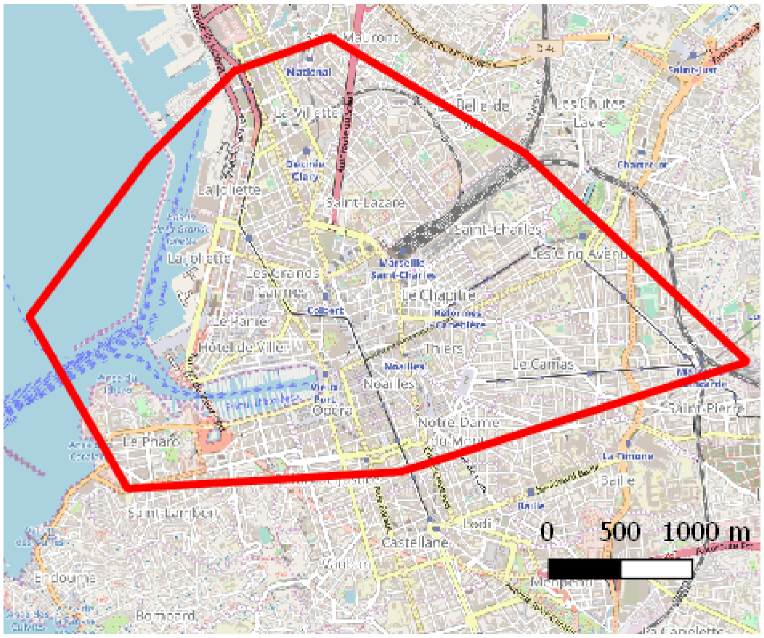

(a) Network exhibit

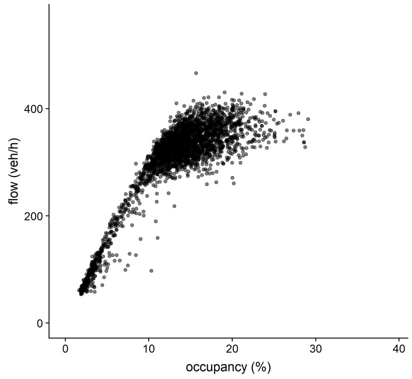

(b) Raw data

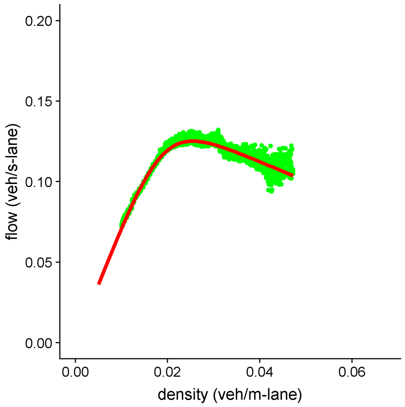

(c) Upper bound and fitted curve

Figure 56: Marseille Region I. (**a**) shows the network exhibit used for the MFD estimation and calculation of network topology variables. The map is oriented to the north and is obtained from OpenStreetMap. (**b**) presents the scatter plot of the raw, i.e., unfiltered, data of the network shown in **a**. This includes all malfunctioning detectors, oversampling of lanes, detectors on residential roads etc. (**c**) shows the upper-bound of the estimated MFD and the fitted curve based on the log-exp function by Ambühl et al. (2018). Flow and density are per lane. Occupancy is in the interval 0 to 1 and means the fraction of time in the observation interval a detector is covered by vehicles, i.e., it approximates density.

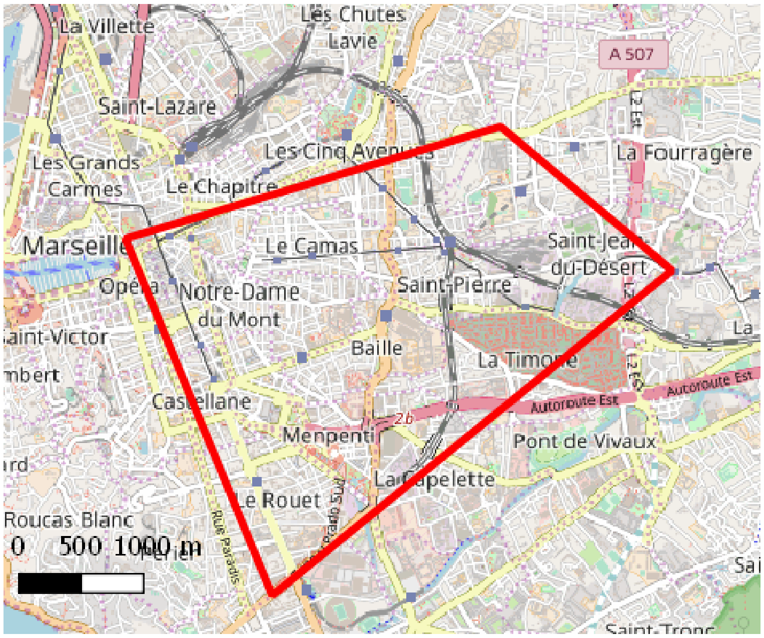

(a) Network exhibit

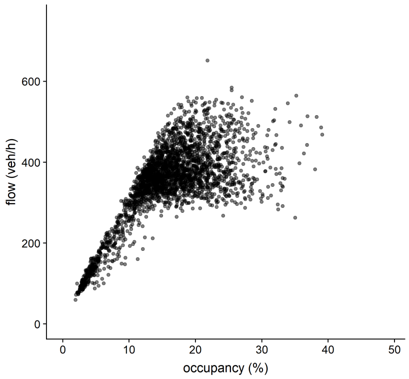

(b) Raw data

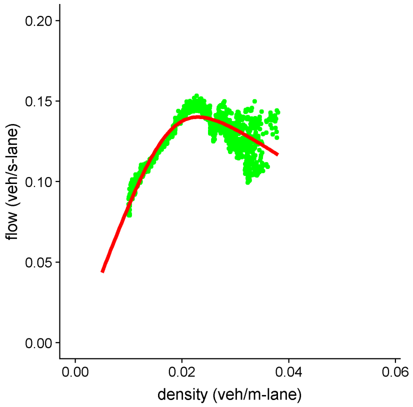

(c) Upper bound and fitted curve

Figure 57: Marseille Region II. (**a**) shows the network exhibit used for the MFD estimation and calculation of network topology variables. The map is oriented to the north and is obtained from OpenStreetMap. (**b**) presents the scatter plot of the raw, i.e., unfiltered, data of the network shown in **a**. This includes all malfunctioning detectors, oversampling of lanes, detectors on residential roads etc. (**c**) shows the upper-bound of the estimated MFD and the fitted curve based on the log-exp function by Ambühl et al. (2018). Flow and density are per lane. Occupancy is in the interval 0 to 1 and means the fraction of time in the observation interval a detector is covered by vehicles, i.e., it approximates density.

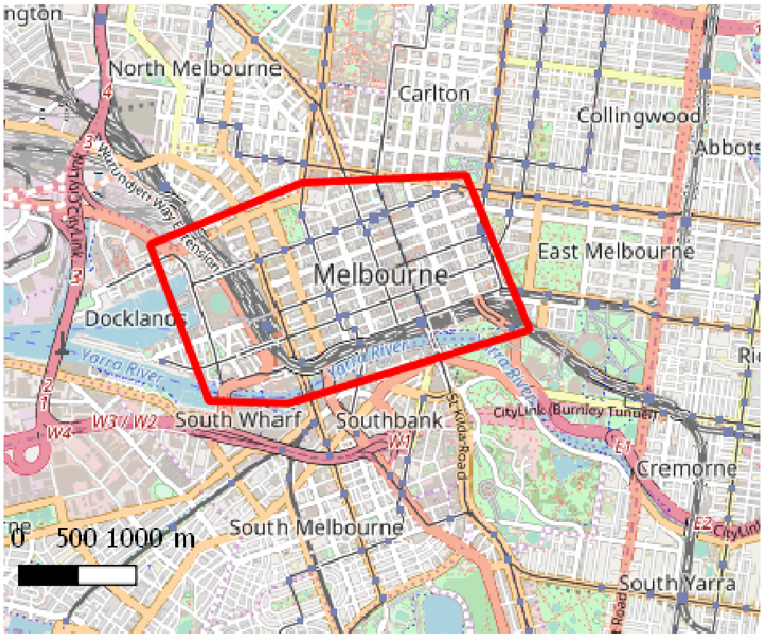

(a) Network exhibit

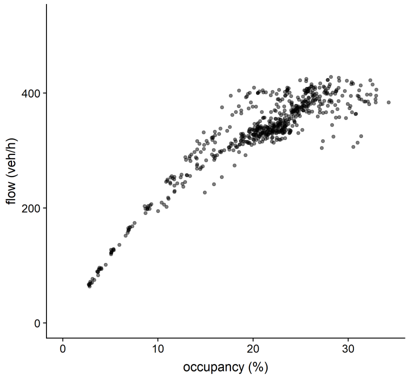

(b) Raw data

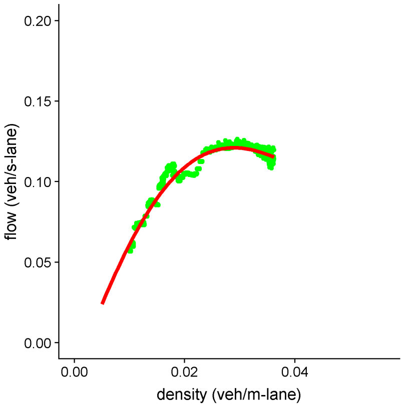

(c) Upper bound and fitted curve

Figure 58: Melbourne Region I. (**a**) shows the network exhibit used for the MFD estimation and calculation of network topology variables. The map is oriented to the north and is obtained from OpenStreetMap. (**b**) presents the scatter plot of the raw, i.e., unfiltered, data of the network shown in **a**. This includes all malfunctioning detectors, oversampling of lanes, detectors on residential roads etc. (**c**) shows the upper-bound of the estimated MFD and the fitted curve based on the log-exp function by Ambühl et al. (2018). Flow and density are per lane. Occupancy is in the interval 0 to 1 and means the fraction of time in the observation interval a detector is covered by vehicles, i.e., it approximates density.

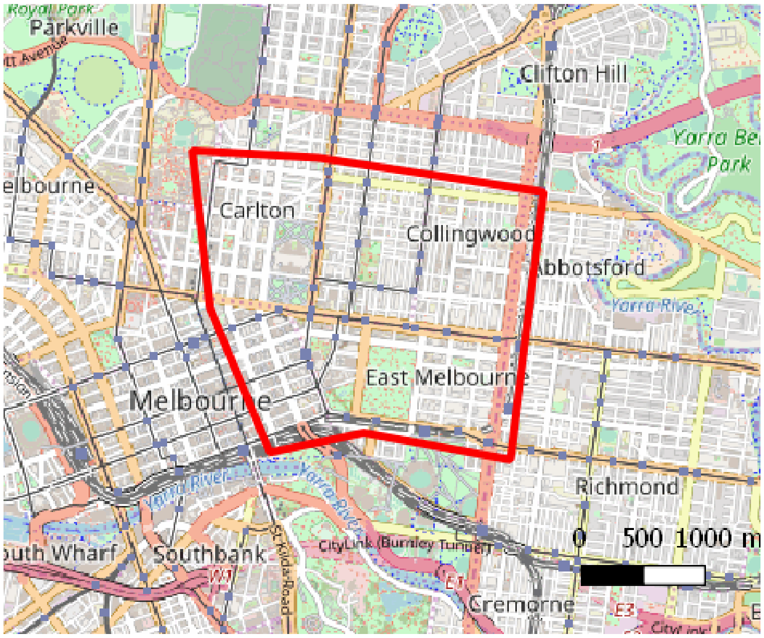

(a) Network exhibit

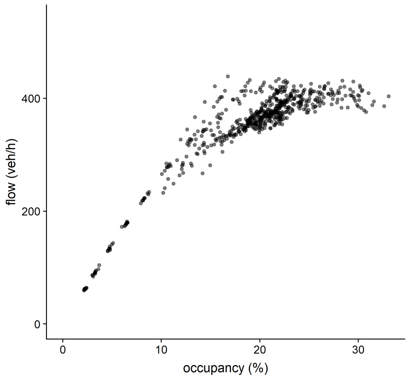

(b) Raw data

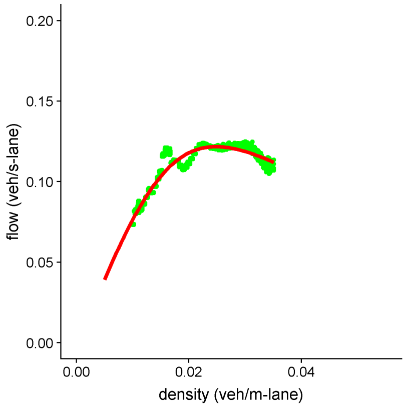

(c) Upper bound and fitted curve

Figure 59: Melbourne Region II. (**a**) shows the network exhibit used for the MFD estimation and calculation of network topology variables. The map is oriented to the north and is obtained from OpenStreetMap. (**b**) presents the scatter plot of the raw, i.e., unfiltered, data of the network shown in **a**. This includes all malfunctioning detectors, oversampling of lanes, detectors on residential roads etc. (**c**) shows the upper-bound of the estimated MFD and the fitted curve based on the log-exp function by Ambühl et al. (2018). Flow and density are per lane. Occupancy is in the interval 0 to 1 and means the fraction of time in the observation interval a detector is covered by vehicles, i.e., it approximates density.

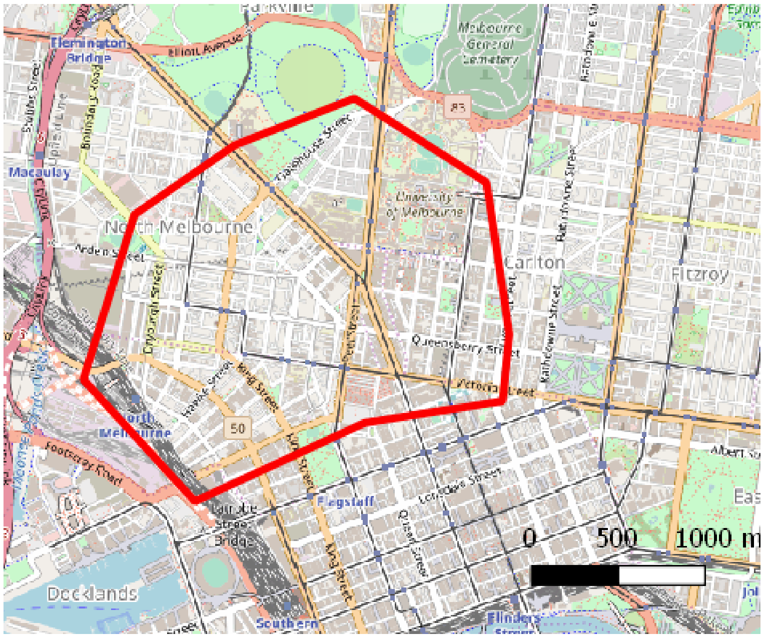

(a) Network exhibit

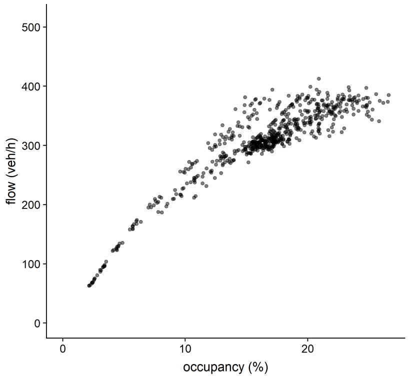

(b) Raw data

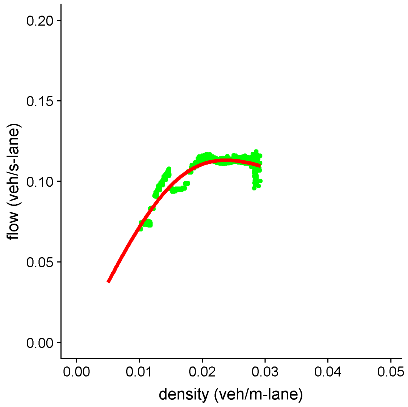

(c) Upper bound and fitted curve

Figure 60: Melbourne Region III. (**a**) shows the network exhibit used for the MFD estimation and calculation of network topology variables. The map is oriented to the north and is obtained from OpenStreetMap. (**b**) presents the scatter plot of the raw, i.e., unfiltered, data of the network shown in **a**. This includes all malfunctioning detectors, oversampling of lanes, detectors on residential roads etc. (**c**) shows the upper-bound of the estimated MFD and the fitted curve based on the log-exp function by Ambühl et al. (2018). Flow and density are per lane. Occupancy is in the interval 0 to 1 and means the fraction of time in the observation interval a detector is covered by vehicles, i.e., it approximates density.

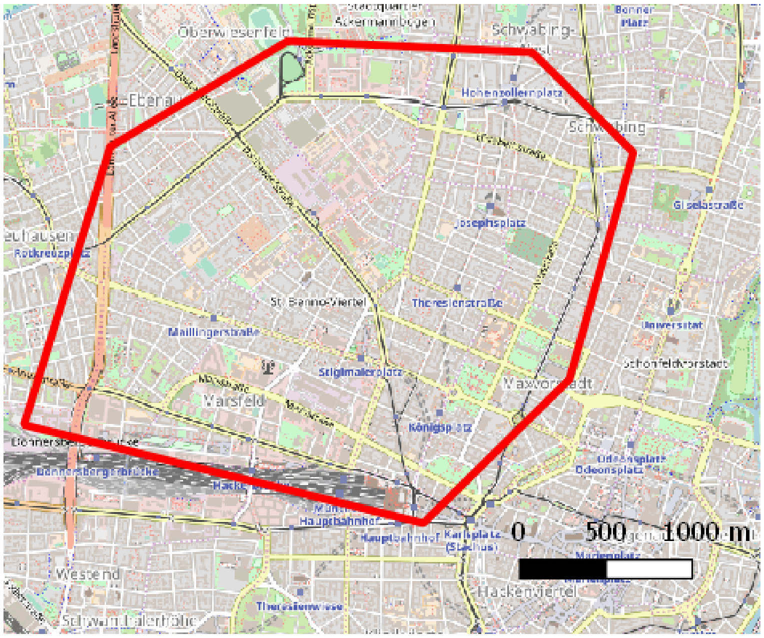

(a) Network exhibit

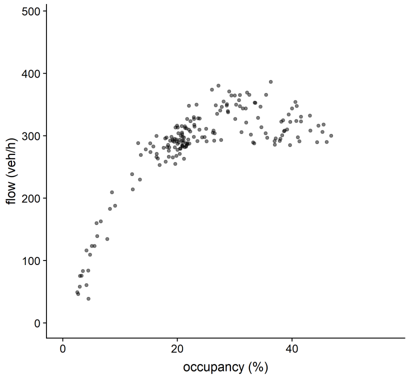

(b) Raw data

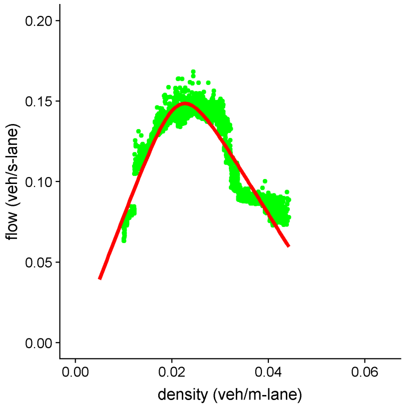

(c) Upper bound and fitted curve

Figure 61: Munich Region I. (**a**) shows the network exhibit used for the MFD estimation and calculation of network topology variables. The map is oriented to the north and is obtained from OpenStreetMap. (**b**) presents the scatter plot of the raw, i.e., unfiltered, data of the network shown in **a**. This includes all malfunctioning detectors, oversampling of lanes, detectors on residential roads etc. (**c**) shows the upper-bound of the estimated MFD and the fitted curve based on the log-exp function by Ambühl et al. (2018). Flow and density are per lane. Occupancy is in the interval 0 to 1 and means the fraction of time in the observation interval a detector is covered by vehicles, i.e., it approximates density.

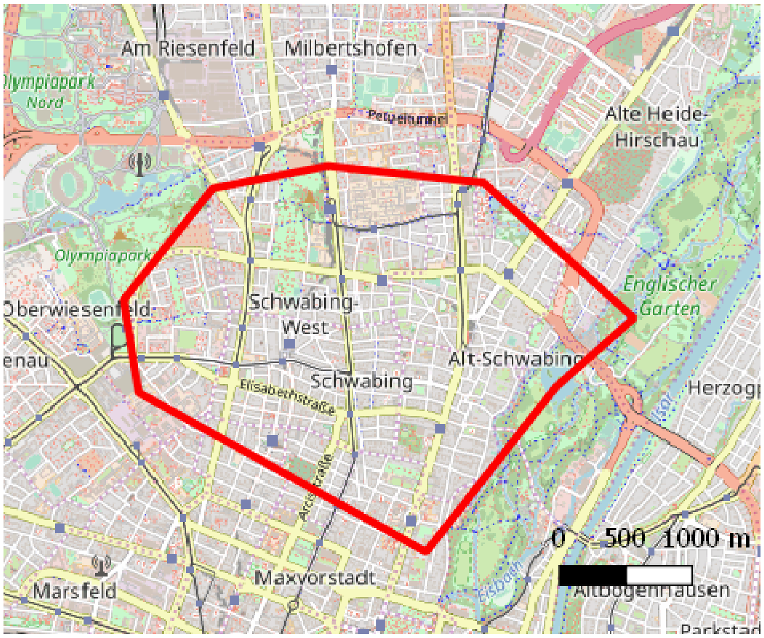

(a) Network exhibit

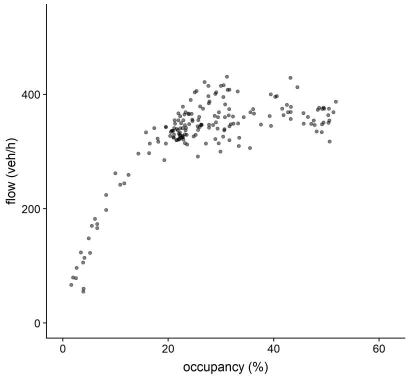

(b) Raw data

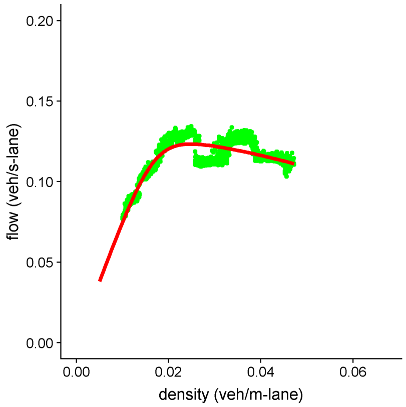

(c) Upper bound and fitted curve

Figure 62: Munich Region II. (**a**) shows the network exhibit used for the MFD estimation and calculation of network topology variables. The map is oriented to the north and is obtained from OpenStreetMap. (**b**) presents the scatter plot of the raw, i.e., unfiltered, data of the network shown in **a**. This includes all malfunctioning detectors, oversampling of lanes, detectors on residential roads etc. (**c**) shows the upper-bound of the estimated MFD and the fitted curve based on the log-exp function by Ambühl et al. (2018). Flow and density are per lane. Occupancy is in the interval 0 to 1 and means the fraction of time in the observation interval a detector is covered by vehicles, i.e., it approximates density.

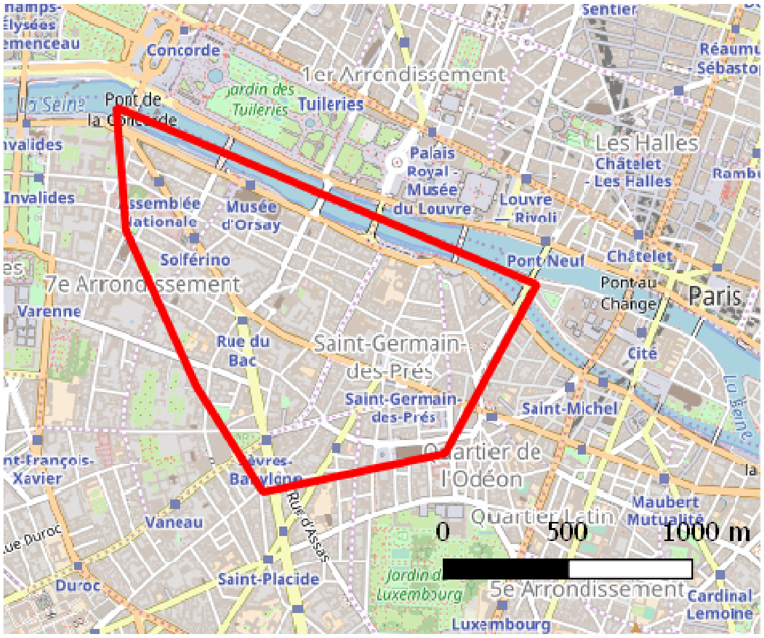

(a) Network exhibit

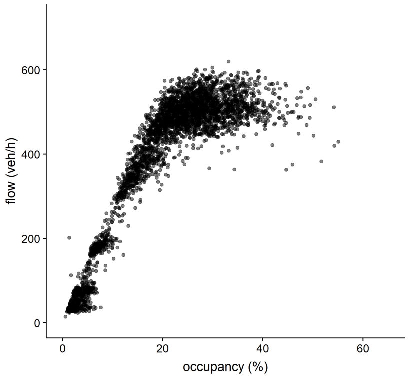

(b) Raw data

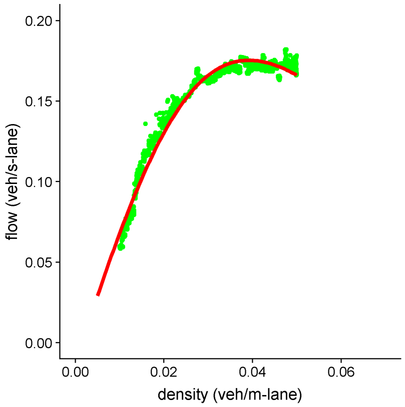

(c) Upper bound and fitted curve

Figure 63: Paris Region I. (**a**) shows the network exhibit used for the MFD estimation and calculation of network topology variables. The map is oriented to the north and is obtained from OpenStreetMap. (**b**) presents the scatter plot of the raw, i.e., unfiltered, data of the network shown in **a**. This includes all malfunctioning detectors, oversampling of lanes, detectors on residential roads etc. (**c**) shows the upper-bound of the estimated MFD and the fitted curve based on the log-exp function by Ambühl et al. (2018). Flow and density are per lane. Occupancy is in the interval 0 to 1 and means the fraction of time in the observation interval a detector is covered by vehicles, i.e., it approximates density.

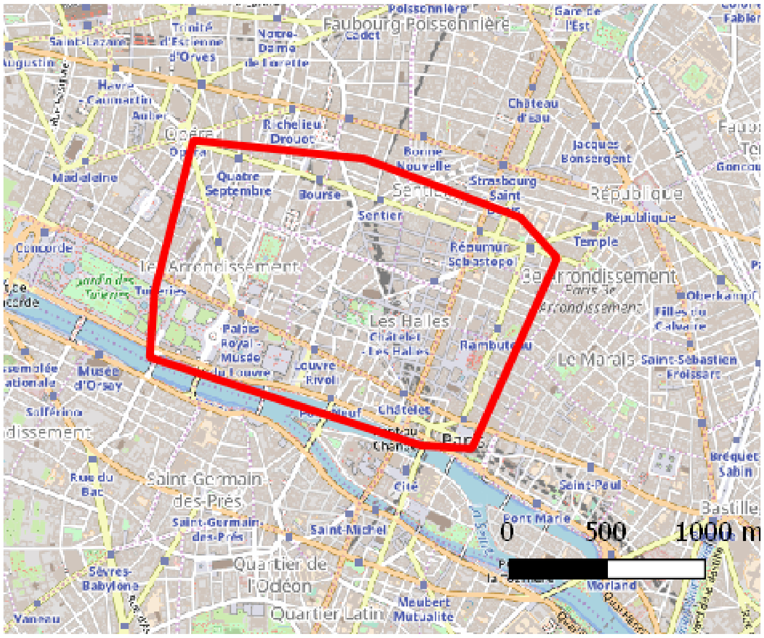

(a) Network exhibit

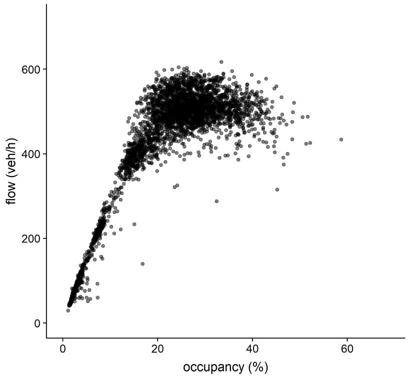

(b) Raw data

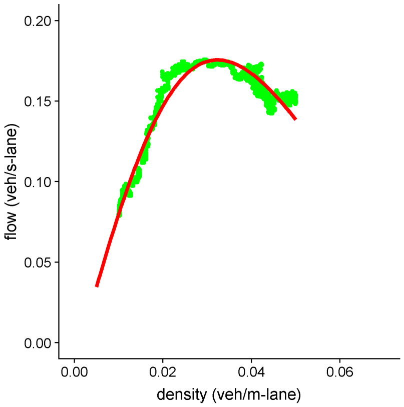

(c) Upper bound and fitted curve

Figure 64: Paris Region II. (**a**) shows the network exhibit used for the MFD estimation and calculation of network topology variables. The map is oriented to the north and is obtained from OpenStreetMap. (**b**) presents the scatter plot of the raw, i.e., unfiltered, data of the network shown in **a**. This includes all malfunctioning detectors, oversampling of lanes, detectors on residential roads etc. (**c**) shows the upper-bound of the estimated MFD and the fitted curve based on the log-exp function by Ambühl et al. (2018). Flow and density are per lane. Occupancy is in the interval 0 to 1 and means the fraction of time in the observation interval a detector is covered by vehicles, i.e., it approximates density.

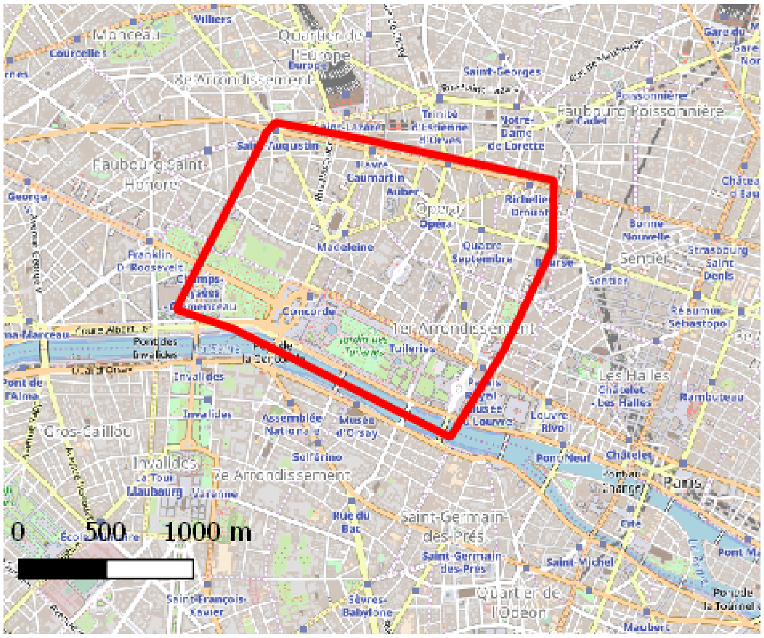

(a) Network exhibit

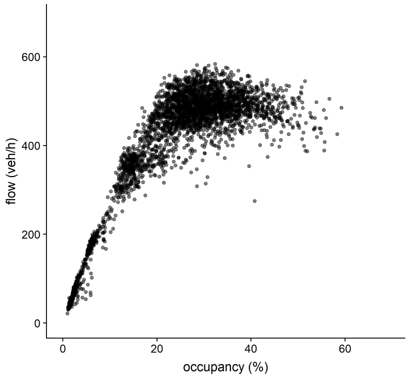

(b) Raw data

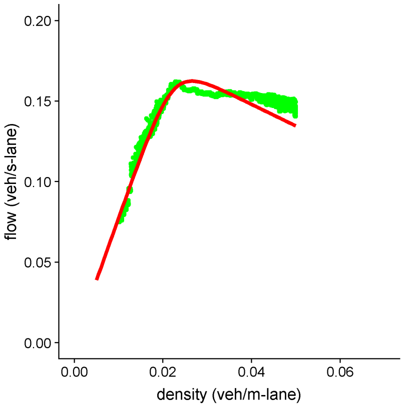

(c) Upper bound and fitted curve

Figure 65: Paris Region III. (**a**) shows the network exhibit used for the MFD estimation and calculation of network topology variables. The map is oriented to the north and is obtained from OpenStreetMap. (**b**) presents the scatter plot of the raw, i.e., unfiltered, data of the network shown in **a**. This includes all malfunctioning detectors, oversampling of lanes, detectors on residential roads etc. (**c**) shows the upper-bound of the estimated MFD and the fitted curve based on the log-exp function by Ambühl et al. (2018). Flow and density are per lane. Occupancy is in the interval 0 to 1 and means the fraction of time in the observation interval a detector is covered by vehicles, i.e., it approximates density.

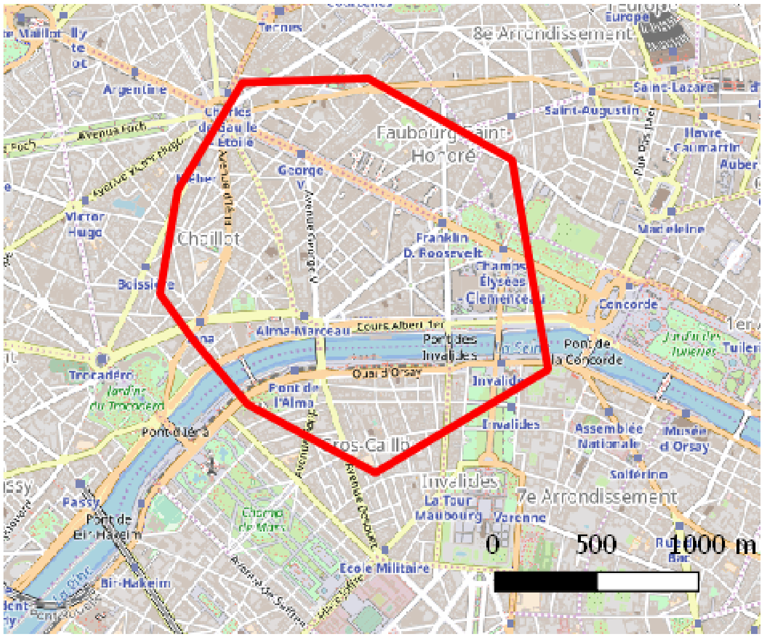

(a) Network exhibit

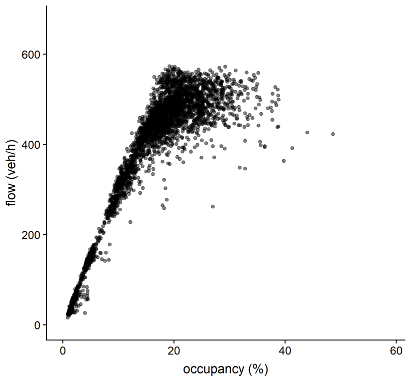

(b) Raw data

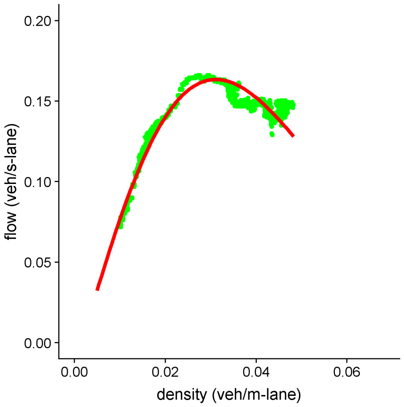

(c) Upper bound and fitted curve

Figure 66: Paris Region IV. (**a**) shows the network exhibit used for the MFD estimation and calculation of network topology variables. The map is oriented to the north and is obtained from OpenStreetMap. (**b**) presents the scatter plot of the raw, i.e., unfiltered, data of the network shown in **a**. This includes all malfunctioning detectors, oversampling of lanes, detectors on residential roads etc. (**c**) shows the upper-bound of the estimated MFD and the fitted curve based on the log-exp function by Ambühl et al. (2018). Flow and density are per lane. Occupancy is in the interval 0 to 1 and means the fraction of time in the observation interval a detector is covered by vehicles, i.e., it approximates density.

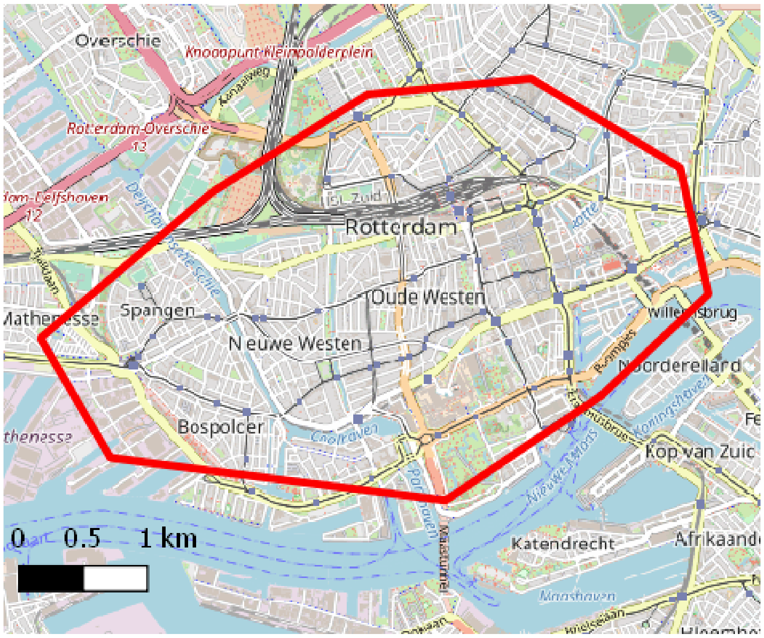

(a) Network exhibit

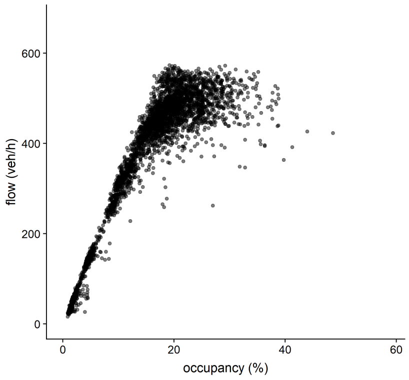

(b) Raw data

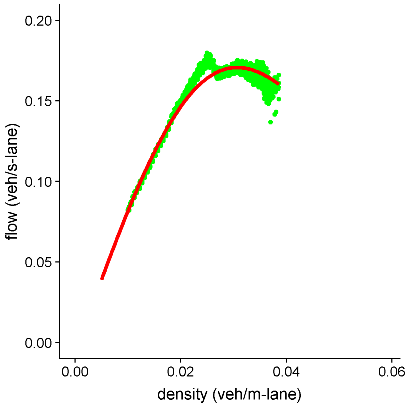

(c) Upper bound and fitted curve

Figure 67: Rotterdam Region I. (**a**) shows the network exhibit used for the MFD estimation and calculation of network topology variables. The map is oriented to the north and is obtained from OpenStreetMap. (**b**) presents the scatter plot of the raw, i.e., unfiltered, data of the network shown in **a**. This includes all malfunctioning detectors, oversampling of lanes, detectors on residential roads etc. (**c**) shows the upper-bound of the estimated MFD and the fitted curve based on the log-exp function by Ambühl et al. (2018). Flow and density are per lane. Occupancy is in the interval 0 to 1 and means the fraction of time in the observation interval a detector is covered by vehicles, i.e., it approximates density.

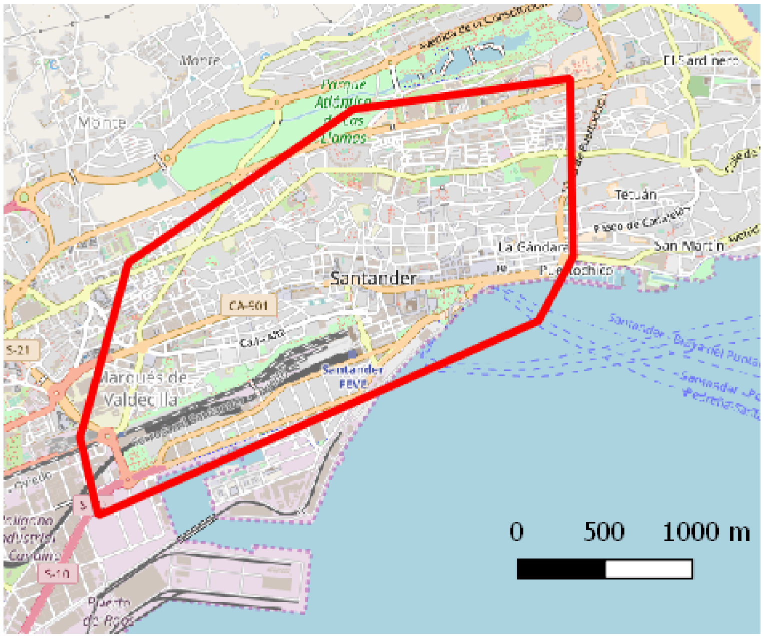

(a) Network exhibit

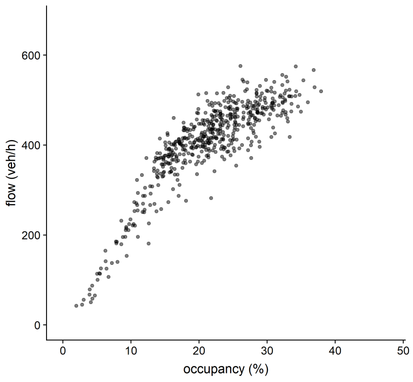

(b) Raw data

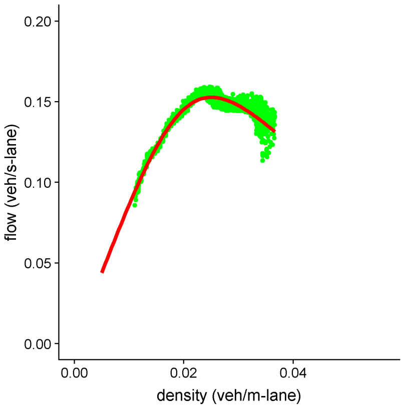

(c) Upper bound and fitted curve

Figure 68: Santander Region I. (**a**) shows the network exhibit used for the MFD estimation and calculation of network topology variables. The map is oriented to the north and is obtained from OpenStreetMap. (**b**) presents the scatter plot of the raw, i.e., unfiltered, data of the network shown in **a**. This includes all malfunctioning detectors, oversampling of lanes, detectors on residential roads etc. (**c**) shows the upper-bound of the estimated MFD and the fitted curve based on the log-exp function by Ambühl et al. (2018). Flow and density are per lane. Occupancy is in the interval 0 to 1 and means the fraction of time in the observation interval a detector is covered by vehicles, i.e., it approximates density.

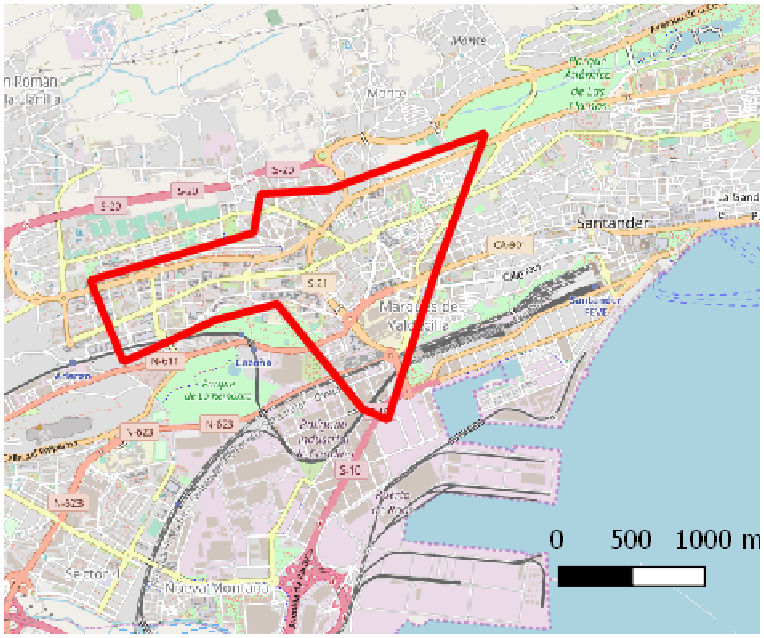

(a) Network exhibit

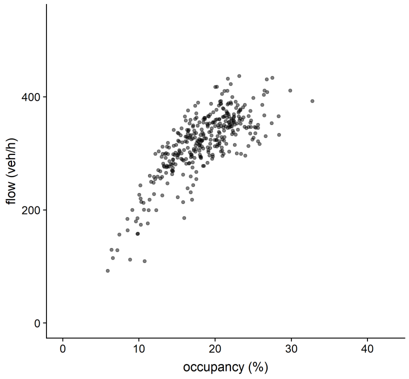

(b) Raw data

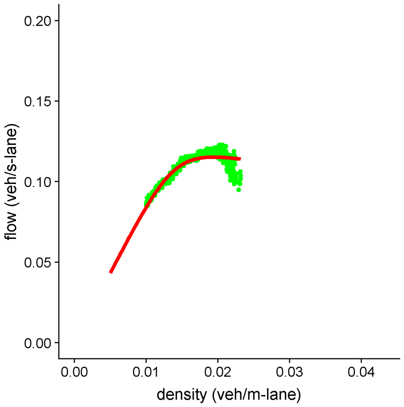

(c) Upper bound and fitted curve

Figure 69: Santander Region II. (**a**) shows the network exhibit used for the MFD estimation and calculation of network topology variables. The map is oriented to the north and is obtained from OpenStreetMap. (**b**) presents the scatter plot of the raw, i.e., unfiltered, data of the network shown in **a**. This includes all malfunctioning detectors, oversampling of lanes, detectors on residential roads etc. (**c**) shows the upper-bound of the estimated MFD and the fitted curve based on the log-exp function by Ambühl et al. (2018). Flow and density are per lane. Occupancy is in the interval 0 to 1 and means the fraction of time in the observation interval a detector is covered by vehicles, i.e., it approximates density.

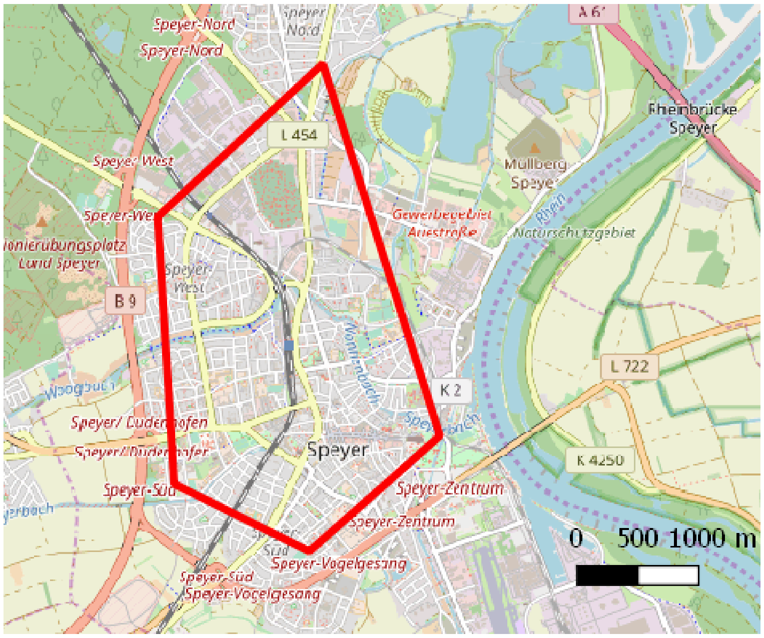

(a) Network exhibit

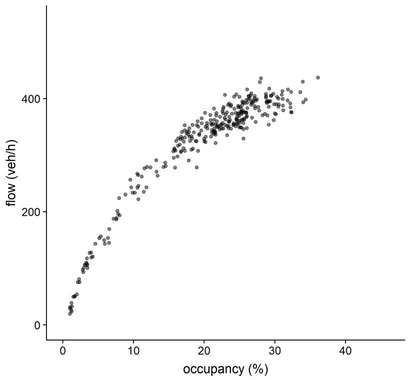

(b) Raw data

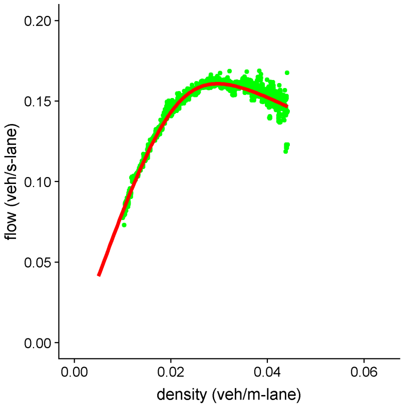

(c) Upper bound and fitted curve

Figure 70: Speyer Region I. (**a**) shows the network exhibit used for the MFD estimation and calculation of network topology variables. The map is oriented to the north and is obtained from OpenStreetMap. (**b**) presents the scatter plot of the raw, i.e., unfiltered, data of the network shown in **a**. This includes all malfunctioning detectors, oversampling of lanes, detectors on residential roads etc. (**c**) shows the upper-bound of the estimated MFD and the fitted curve based on the log-exp function by Ambühl et al. (2018). Flow and density are per lane. Occupancy is in the interval 0 to 1 and means the fraction of time in the observation interval a detector is covered by vehicles, i.e., it approximates density.

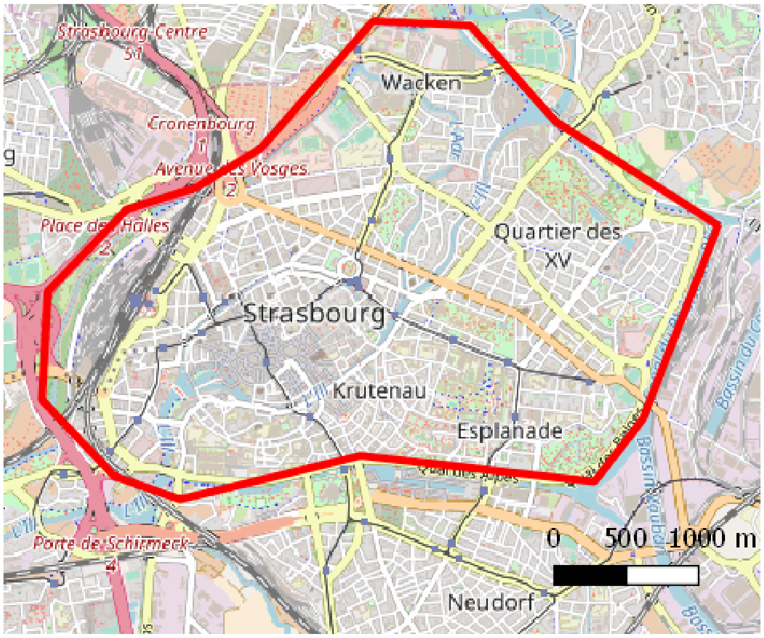

(a) Network exhibit

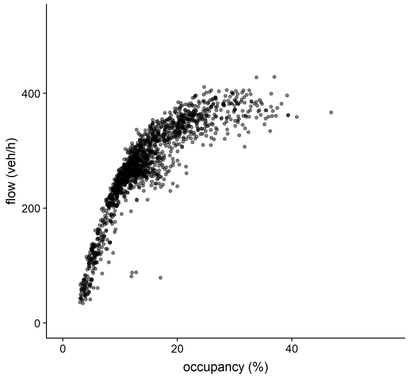

(b) Raw data

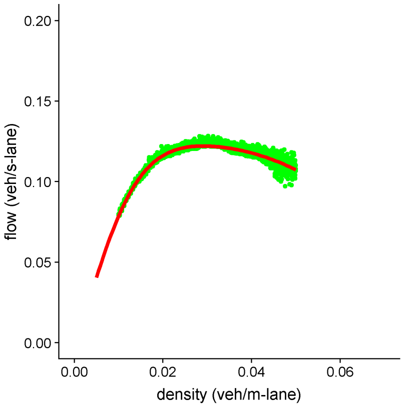

(c) Upper bound and fitted curve

Figure 71: Strasbourg Region I. (**a**) shows the network exhibit used for the MFD estimation and calculation of network topology variables. The map is oriented to the north and is obtained from OpenStreetMap. (**b**) presents the scatter plot of the raw, i.e., unfiltered, data of the network shown in **a**. This includes all malfunctioning detectors, oversampling of lanes, detectors on residential roads etc. (**c**) shows the upper-bound of the estimated MFD and the fitted curve based on the log-exp function by Ambühl et al. (2018). Flow and density are per lane. Occupancy is in the interval 0 to 1 and means the fraction of time in the observation interval a detector is covered by vehicles, i.e., it approximates density.

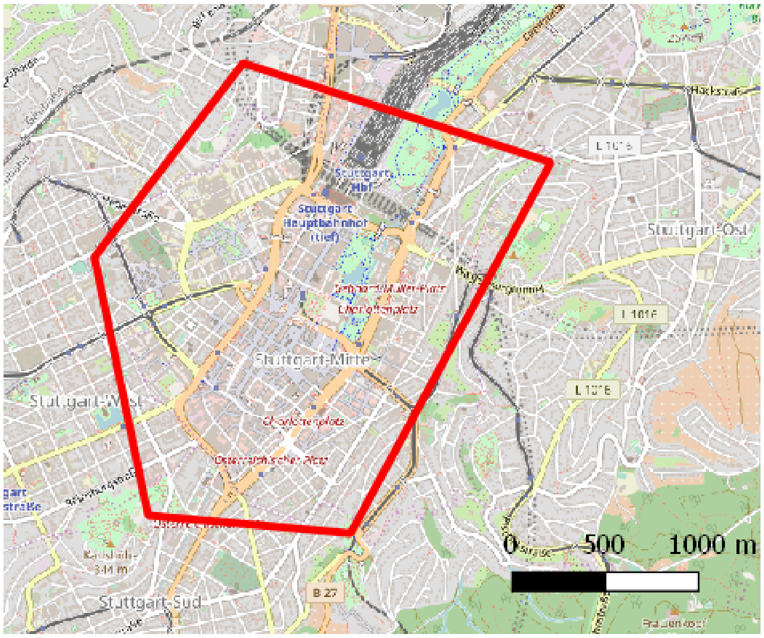

(a) Network exhibit

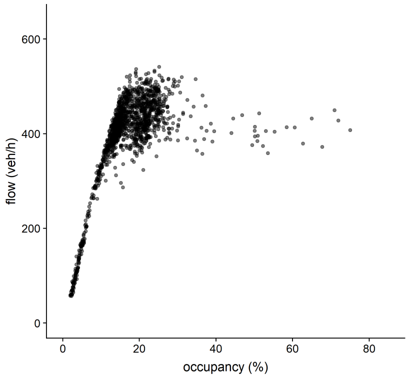

(b) Raw data

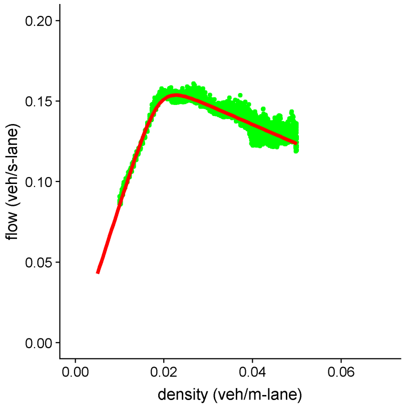

(c) Upper bound and fitted curve

Figure 72: Stuttgart Region I. (**a**) shows the network exhibit used for the MFD estimation and calculation of network topology variables. The map is oriented to the north and is obtained from OpenStreetMap. (**b**) presents the scatter plot of the raw, i.e., unfiltered, data of the network shown in **a**. This includes all malfunctioning detectors, oversampling of lanes, detectors on residential roads etc. (**c**) shows the upper-bound of the estimated MFD and the fitted curve based on the log-exp function by Ambühl et al. (2018). Flow and density are per lane. Occupancy is in the interval 0 to 1 and means the fraction of time in the observation interval a detector is covered by vehicles, i.e., it approximates density.

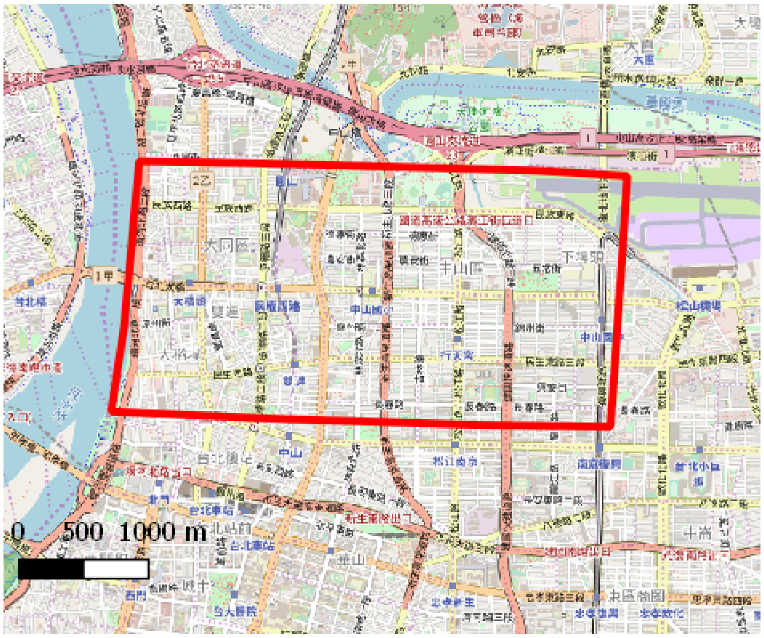

(a) Network exhibit

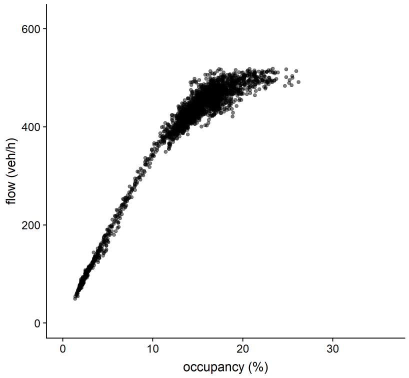

(b) Raw data

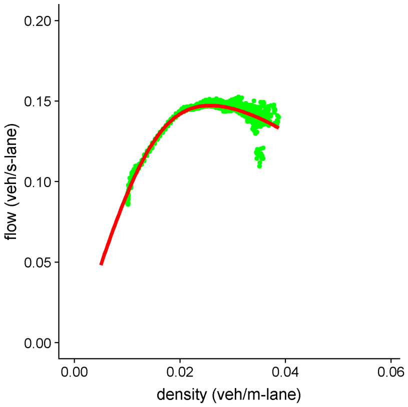

(c) Upper bound and fitted curve

Figure 73: Taipei Region I. (**a**) shows the network exhibit used for the MFD estimation and calculation of network topology variables. The map is oriented to the north and is obtained from OpenStreetMap. (**b**) presents the scatter plot of the raw, i.e., unfiltered, data of the network shown in **a**. This includes all malfunctioning detectors, oversampling of lanes, detectors on residential roads etc. (**c**) shows the upper-bound of the estimated MFD and the fitted curve based on the log-exp function by Ambühl et al. (2018). Flow and density are per lane. Occupancy is in the interval 0 to 1 and means the fraction of time in the observation interval a detector is covered by vehicles, i.e., it approximates density.

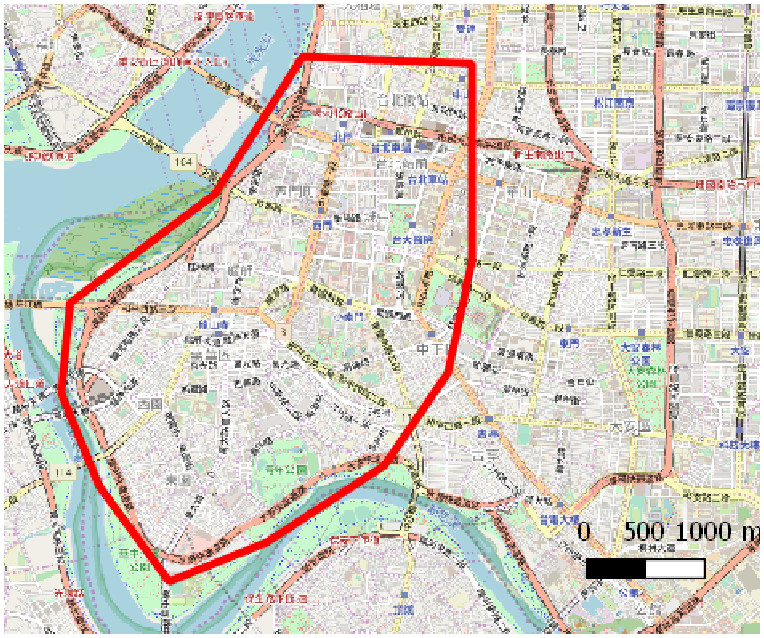

(a) Network exhibit

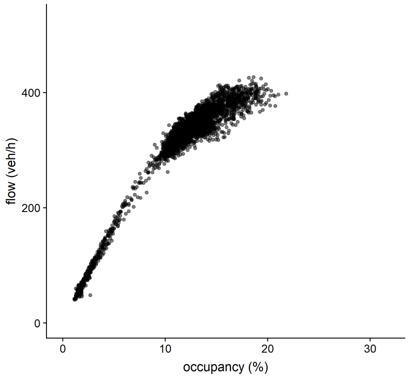

(b) Raw data

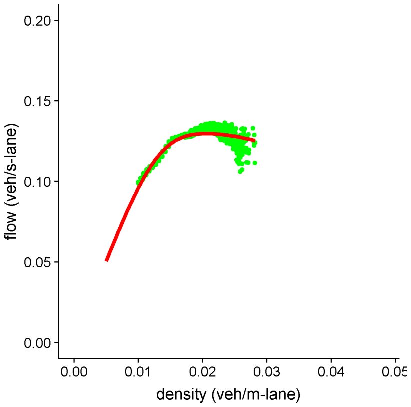

(c) Upper bound and fitted curve

Figure 74: Taipei Region II. (**a**) shows the network exhibit used for the MFD estimation and calculation of network topology variables. The map is oriented to the north and is obtained from OpenStreetMap. (**b**) presents the scatter plot of the raw, i.e., unfiltered, data of the network shown in **a**. This includes all malfunctioning detectors, oversampling of lanes, detectors on residential roads etc. (**c**) shows the upper-bound of the estimated MFD and the fitted curve based on the log-exp function by Ambühl et al. (2018). Flow and density are per lane. Occupancy is in the interval 0 to 1 and means the fraction of time in the observation interval a detector is covered by vehicles, i.e., it approximates density.

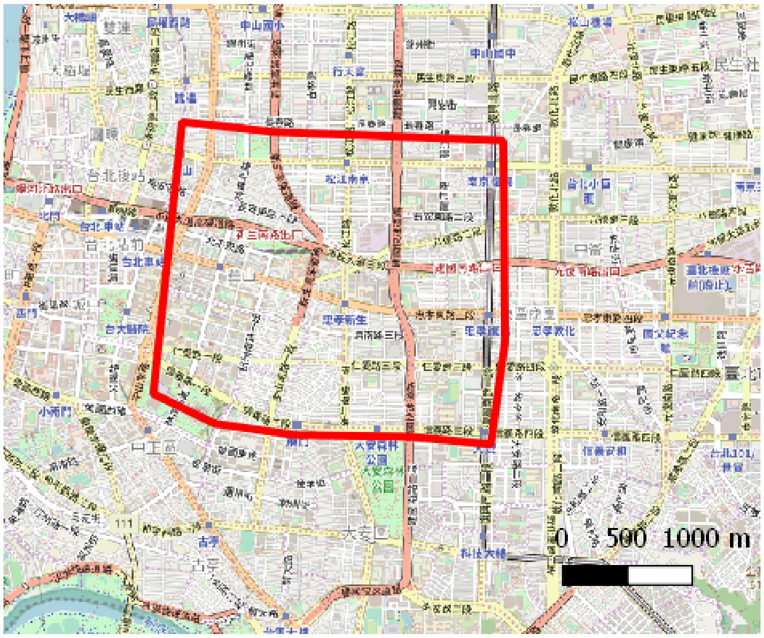

(a) Network exhibit

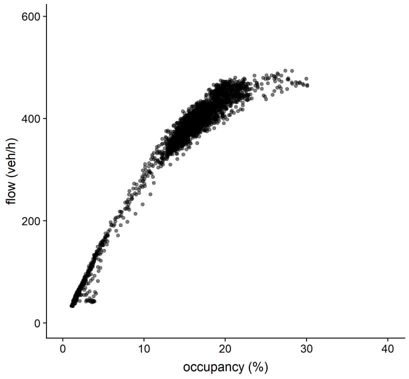

(b) Raw data

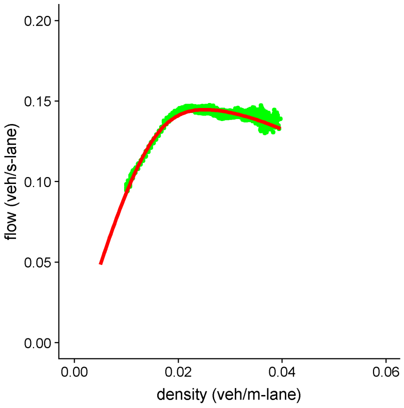

(c) Upper bound and fitted curve

Figure 75: Taipei Region III. (**a**) shows the network exhibit used for the MFD estimation and calculation of network topology variables. The map is oriented to the north and is obtained from OpenStreetMap. (**b**) presents the scatter plot of the raw, i.e., unfiltered, data of the network shown in **a**. This includes all malfunctioning detectors, oversampling of lanes, detectors on residential roads etc. (**c**) shows the upper-bound of the estimated MFD and the fitted curve based on the log-exp function by Ambühl et al. (2018). Flow and density are per lane. Occupancy is in the interval 0 to 1 and means the fraction of time in the observation interval a detector is covered by vehicles, i.e., it approximates density.

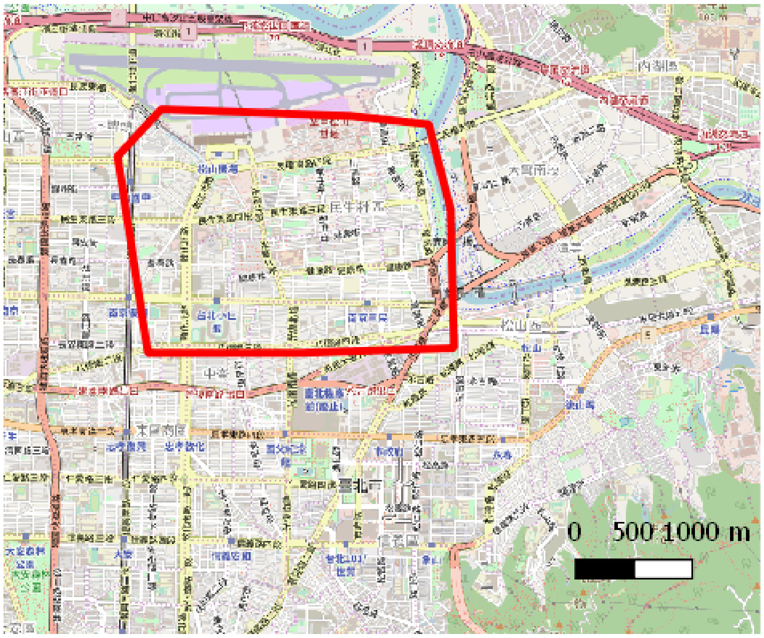

(a) Network exhibit

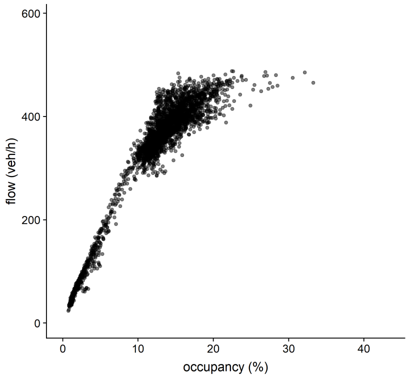

(b) Raw data

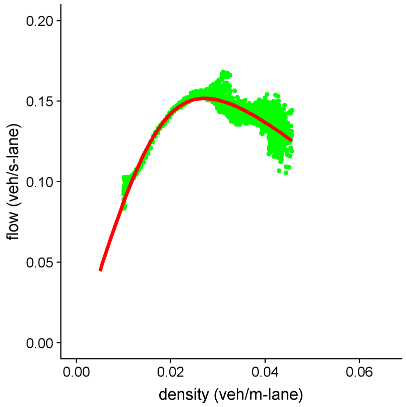

(c) Upper bound and fitted curve

Figure 76: Taipei Region IV. (**a**) shows the network exhibit used for the MFD estimation and calculation of network topology variables. The map is oriented to the north and is obtained from OpenStreetMap. (**b**) presents the scatter plot of the raw, i.e., unfiltered, data of the network shown in **a**. This includes all malfunctioning detectors, oversampling of lanes, detectors on residential roads etc. (**c**) shows the upper-bound of the estimated MFD and the fitted curve based on the log-exp function by Ambühl et al. (2018). Flow and density are per lane. Occupancy is in the interval 0 to 1 and means the fraction of time in the observation interval a detector is covered by vehicles, i.e., it approximates density.

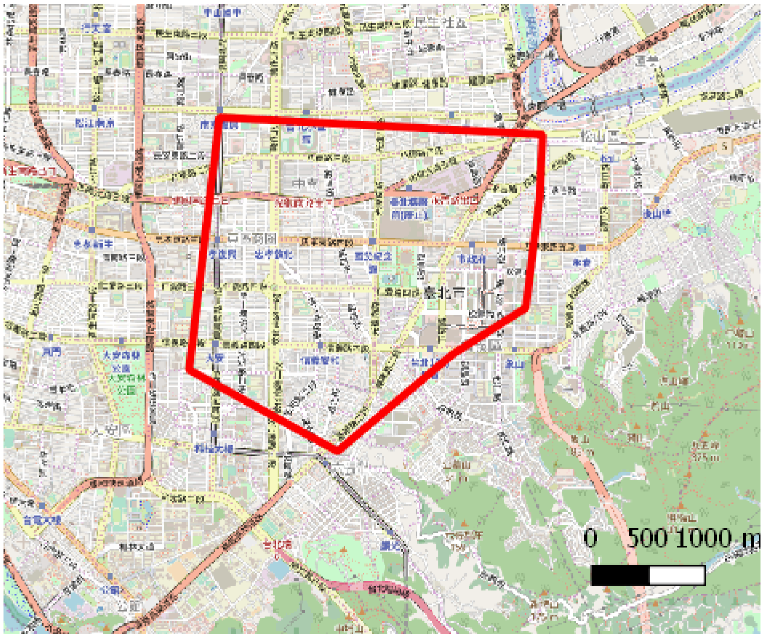

(a) Network exhibit

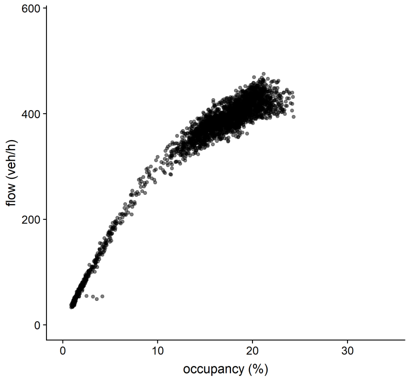

(b) Raw data

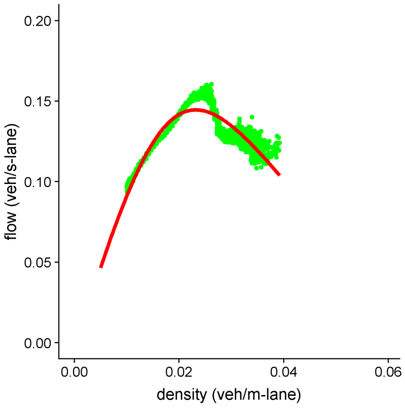

(c) Upper bound and fitted curve

Figure 77: Taipei Region V. (**a**) shows the network exhibit used for the MFD estimation and calculation of network topology variables. The map is oriented to the north and is obtained from OpenStreetMap. (**b**) presents the scatter plot of the raw, i.e., unfiltered, data of the network shown in **a**. This includes all malfunctioning detectors, oversampling of lanes, detectors on residential roads etc. (**c**) shows the upper-bound of the estimated MFD and the fitted curve based on the log-exp function by Ambühl et al. (2018). Flow and density are per lane. Occupancy is in the interval 0 to 1 and means the fraction of time in the observation interval a detector is covered by vehicles, i.e., it approximates density.

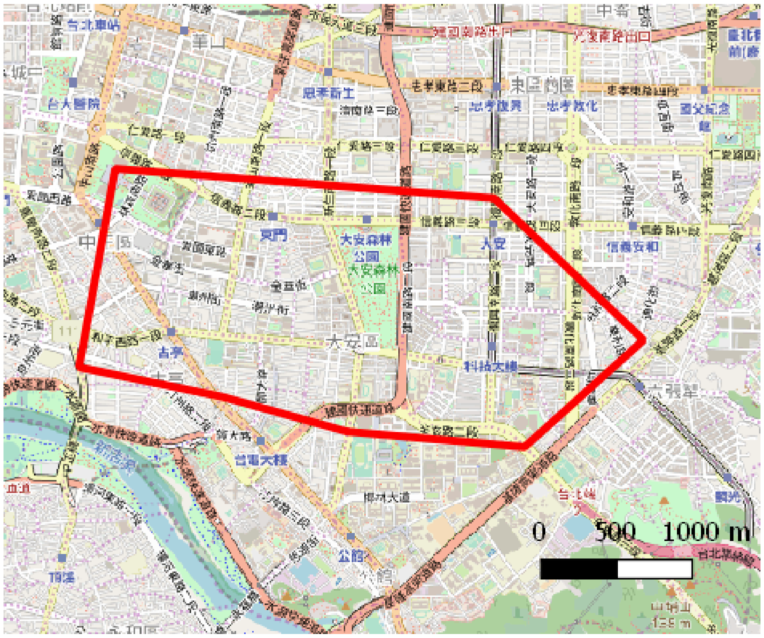

(a) Network exhibit

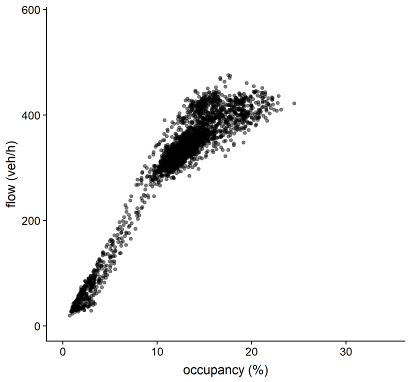

(b) Raw data

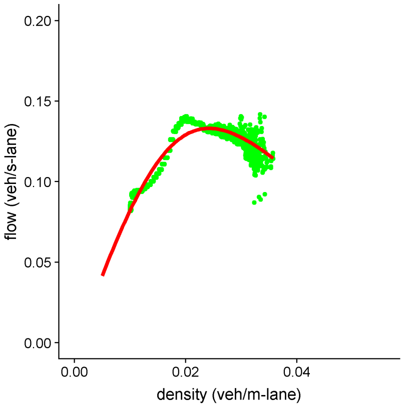

(c) Upper bound and fitted curve

Figure 78: Taipei Region VI. (**a**) shows the network exhibit used for the MFD estimation and calculation of network topology variables. The map is oriented to the north and is obtained from OpenStreetMap. (**b**) presents the scatter plot of the raw, i.e., unfiltered, data of the network shown in **a**. This includes all malfunctioning detectors, oversampling of lanes, detectors on residential roads etc. (**c**) shows the upper-bound of the estimated MFD and the fitted curve based on the log-exp function by Ambühl et al. (2018). Flow and density are per lane. Occupancy is in the interval 0 to 1 and means the fraction of time in the observation interval a detector is covered by vehicles, i.e., it approximates density.

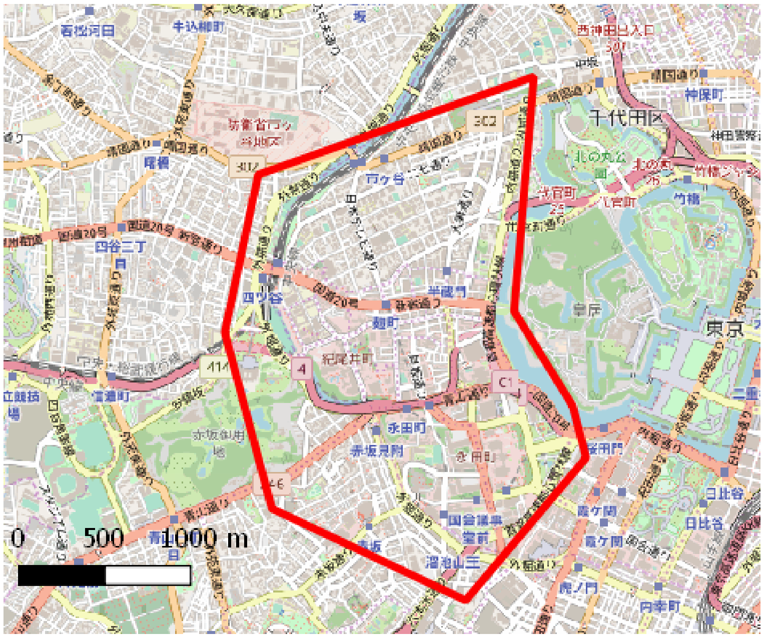

(a) Network exhibit

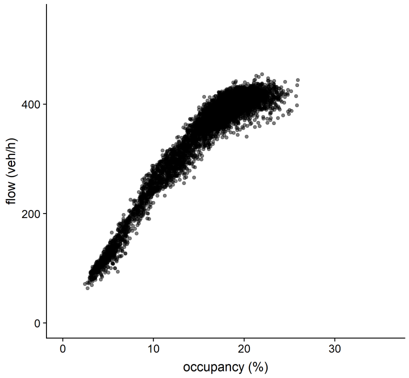

(b) Raw data

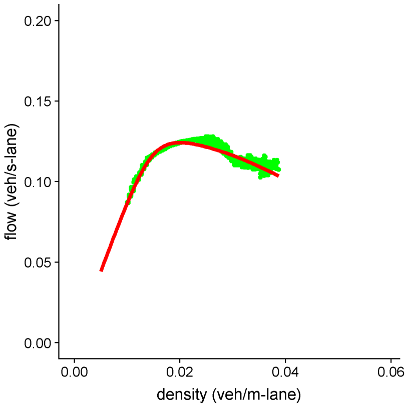

(c) Upper bound and fitted curve

Figure 79: Tokyo Region I. (**a**) shows the network exhibit used for the MFD estimation and calculation of network topology variables. The map is oriented to the north and is obtained from OpenStreetMap. (**b**) presents the scatter plot of the raw, i.e., unfiltered, data of the network shown in **a**. This includes all malfunctioning detectors, oversampling of lanes, detectors on residential roads etc. (**c**) shows the upper-bound of the estimated MFD and the fitted curve based on the log-exp function by Ambühl et al. (2018). Flow and density are per lane. Occupancy is in the interval 0 to 1 and means the fraction of time in the observation interval a detector is covered by vehicles, i.e., it approximates density.

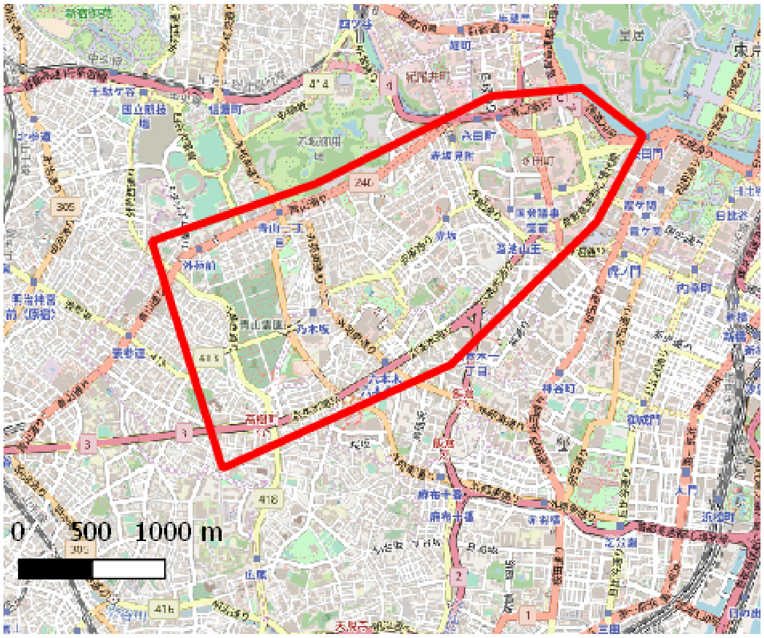

(a) Network exhibit

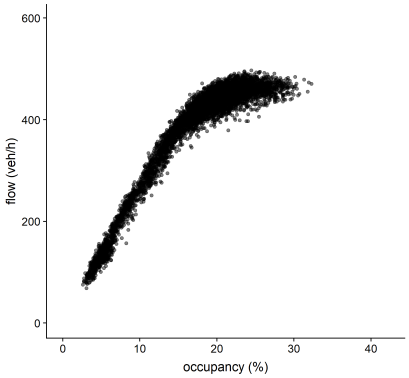

(b) Raw data

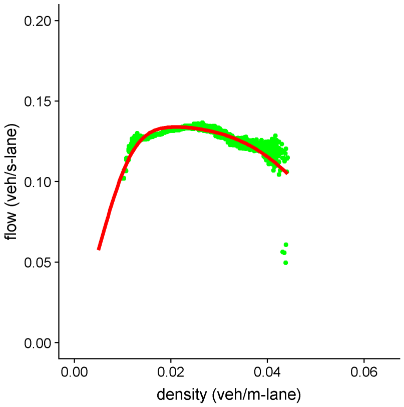

(c) Upper bound and fitted curve

Figure 80: Tokyo Region II. (**a**) shows the network exhibit used for the MFD estimation and calculation of network topology variables. The map is oriented to the north and is obtained from OpenStreetMap. (**b**) presents the scatter plot of the raw, i.e., unfiltered, data of the network shown in **a**. This includes all malfunctioning detectors, oversampling of lanes, detectors on residential roads etc. (**c**) shows the upper-bound of the estimated MFD and the fitted curve based on the log-exp function by Ambühl et al. (2018). Flow and density are per lane. Occupancy is in the interval 0 to 1 and means the fraction of time in the observation interval a detector is covered by vehicles, i.e., it approximates density.

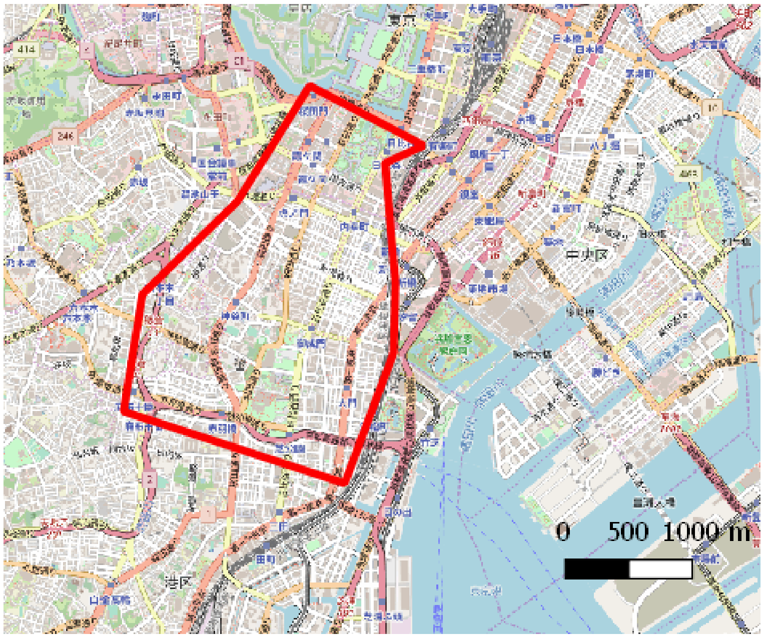

(a) Network exhibit

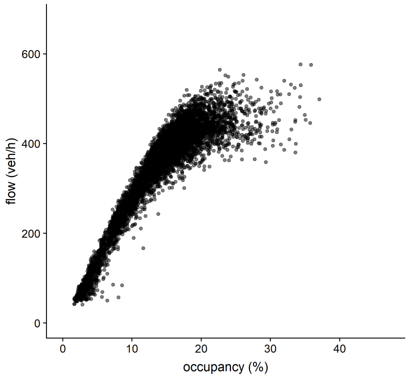

(b) Raw data

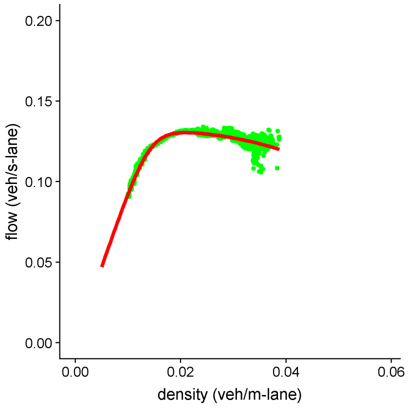

(c) Upper bound and fitted curve

Figure 81: Tokyo Region III. (**a**) shows the network exhibit used for the MFD estimation and calculation of network topology variables. The map is oriented to the north and is obtained from OpenStreetMap. (**b**) presents the scatter plot of the raw, i.e., unfiltered, data of the network shown in **a**. This includes all malfunctioning detectors, oversampling of lanes, detectors on residential roads etc. (**c**) shows the upper-bound of the estimated MFD and the fitted curve based on the log-exp function by Ambühl et al. (2018). Flow and density are per lane. Occupancy is in the interval 0 to 1 and means the fraction of time in the observation interval a detector is covered by vehicles, i.e., it approximates density.

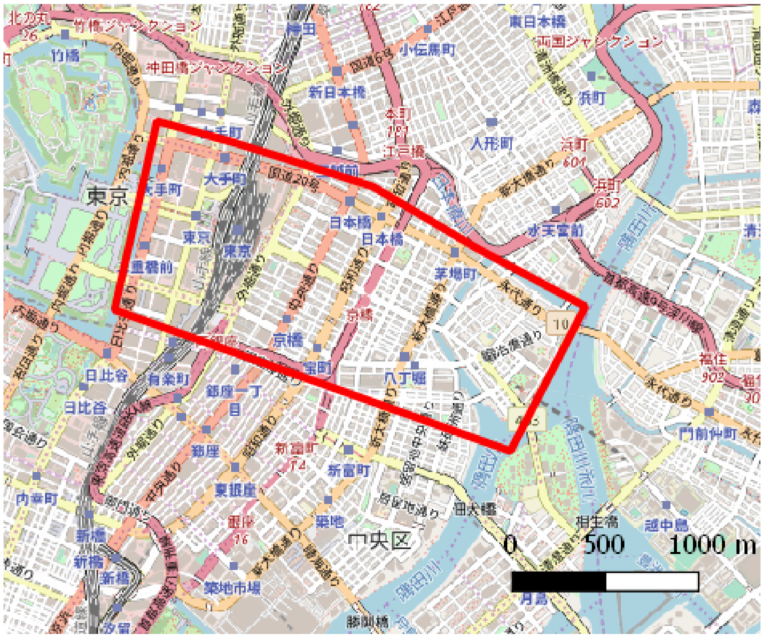

(a) Network exhibit

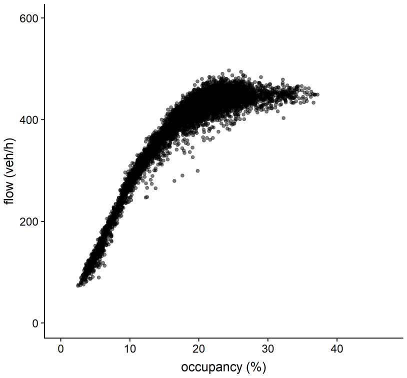

(b) Raw data

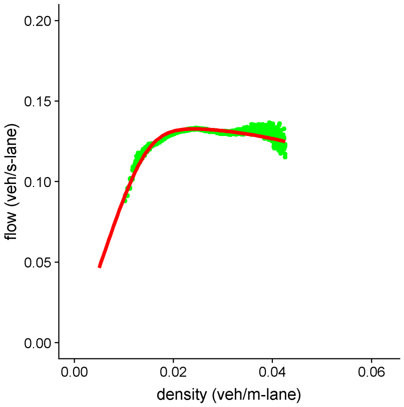

(c) Upper bound and fitted curve

Figure 82: Tokyo Region IV. (**a**) shows the network exhibit used for the MFD estimation and calculation of network topology variables. The map is oriented to the north and is obtained from OpenStreetMap. (**b**) presents the scatter plot of the raw, i.e., unfiltered, data of the network shown in **a**. This includes all malfunctioning detectors, oversampling of lanes, detectors on residential roads etc. (**c**) shows the upper-bound of the estimated MFD and the fitted curve based on the log-exp function by Ambühl et al. (2018). Flow and density are per lane. Occupancy is in the interval 0 to 1 and means the fraction of time in the observation interval a detector is covered by vehicles, i.e., it approximates density.

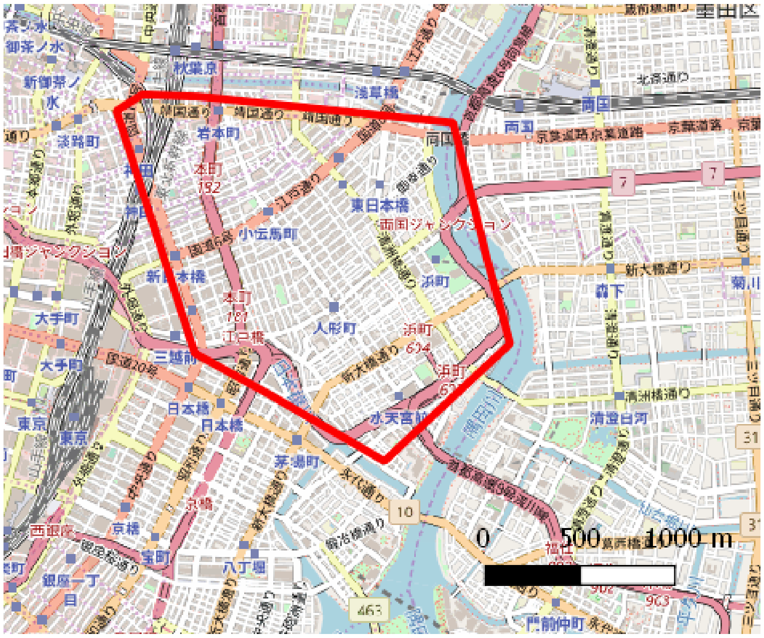

(a) Network exhibit

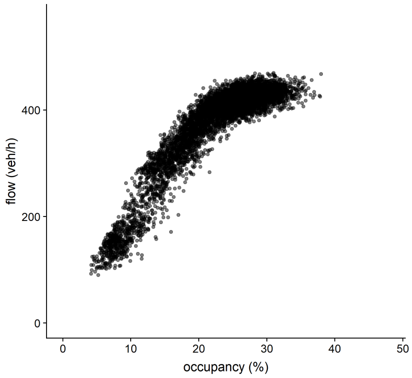

(b) Raw data

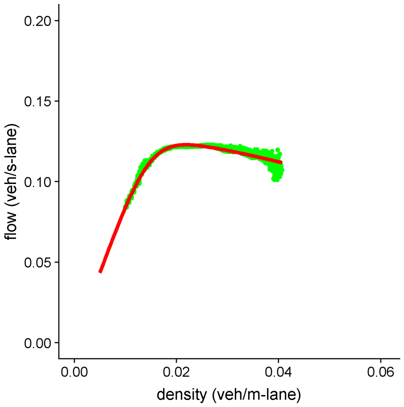

(c) Upper bound and fitted curve

Figure 83: Tokyo Region V. (**a**) shows the network exhibit used for the MFD estimation and calculation of network topology variables. The map is oriented to the north and is obtained from OpenStreetMap. (**b**) presents the scatter plot of the raw, i.e., unfiltered, data of the network shown in **a**. This includes all malfunctioning detectors, oversampling of lanes, detectors on residential roads etc. (**c**) shows the upper-bound of the estimated MFD and the fitted curve based on the log-exp function by Ambühl et al. (2018). Flow and density are per lane. Occupancy is in the interval 0 to 1 and means the fraction of time in the observation interval a detector is covered by vehicles, i.e., it approximates density.

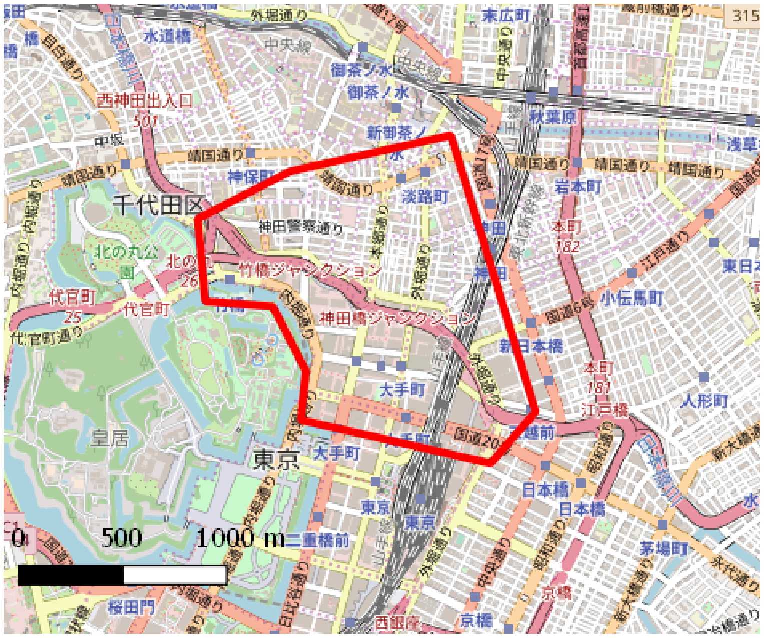

(a) Network exhibit

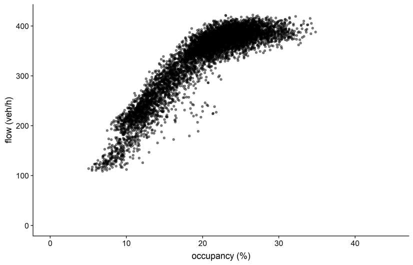

(b) Raw data

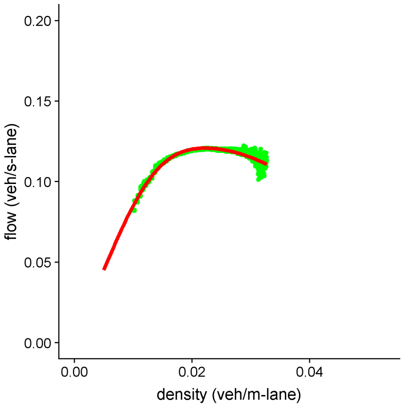

(c) Upper bound and fitted curve

Figure 84: Tokyo Region VI. (**a**) shows the network exhibit used for the MFD estimation and calculation of network topology variables. The map is oriented to the north and is obtained from OpenStreetMap. (**b**) presents the scatter plot of the raw, i.e., unfiltered, data of the network shown in **a**. This includes all malfunctioning detectors, oversampling of lanes, detectors on residential roads etc. (**c**) shows the upper-bound of the estimated MFD and the fitted curve based on the log-exp function by Ambühl et al. (2018). Flow and density are per lane. Occupancy is in the interval 0 to 1 and means the fraction of time in the observation interval a detector is covered by vehicles, i.e., it approximates density.

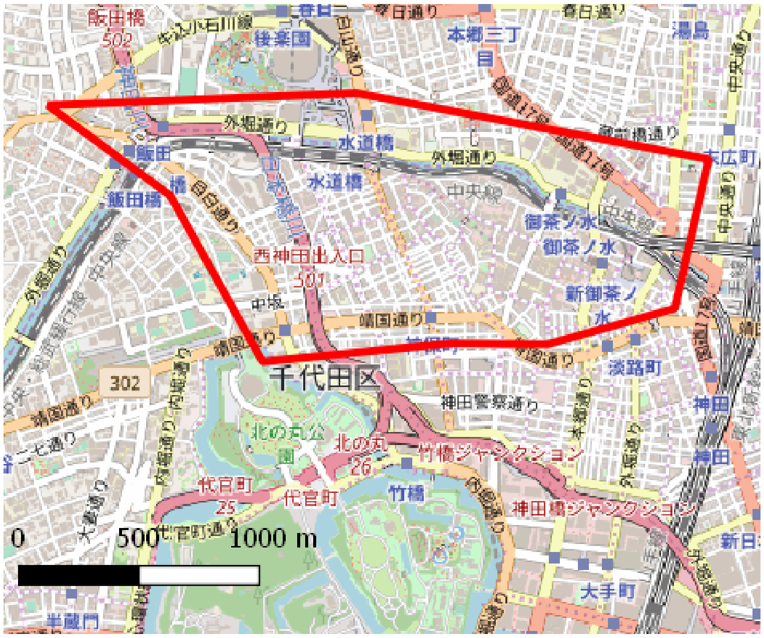

(a) Network exhibit

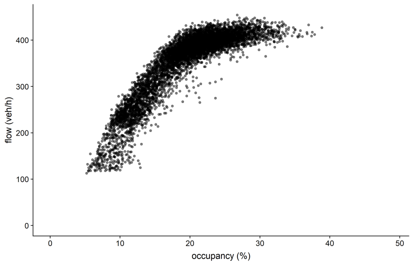

(b) Raw data

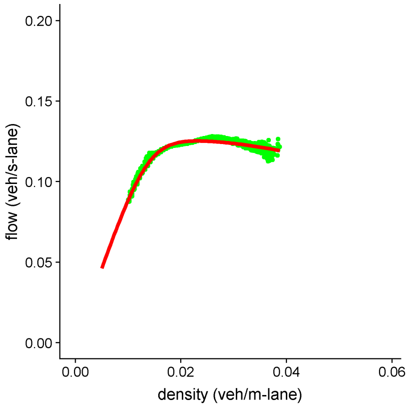

(c) Upper bound and fitted curve

Figure 85: Tokyo Region VII. (**a**) shows the network exhibit used for the MFD estimation and calculation of network topology variables. The map is oriented to the north and is obtained from OpenStreetMap. (**b**) presents the scatter plot of the raw, i.e., unfiltered, data of the network shown in **a**. This includes all malfunctioning detectors, oversampling of lanes, detectors on residential roads etc. (**c**) shows the upper-bound of the estimated MFD and the fitted curve based on the log-exp function by Ambühl et al. (2018). Flow and density are per lane. Occupancy is in the interval 0 to 1 and means the fraction of time in the observation interval a detector is covered by vehicles, i.e., it approximates density.

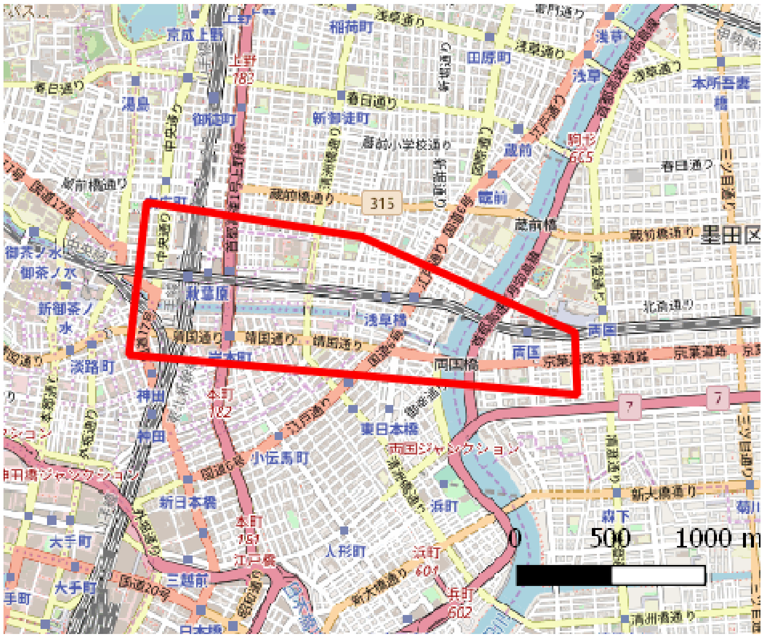

(a) Network exhibit

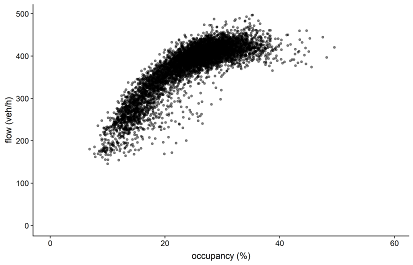

(b) Raw data

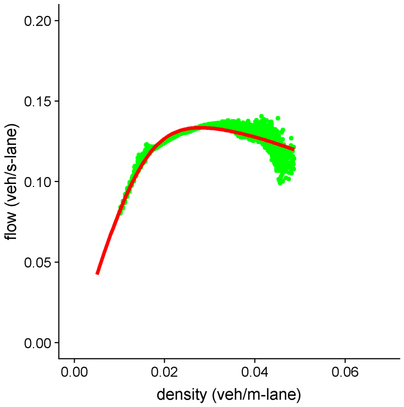

(c) Upper bound and fitted curve

Figure 86: Tokyo Region VIII. (**a**) shows the network exhibit used for the MFD estimation and calculation of network topology variables. The map is oriented to the north and is obtained from OpenStreetMap. (**b**) presents the scatter plot of the raw, i.e., unfiltered, data of the network shown in **a**. This includes all malfunctioning detectors, oversampling of lanes, detectors on residential roads etc. (**c**) shows the upper-bound of the estimated MFD and the fitted curve based on the log-exp function by Ambühl et al. (2018). Flow and density are per lane. Occupancy is in the interval 0 to 1 and means the fraction of time in the observation interval a detector is covered by vehicles, i.e., it approximates density.

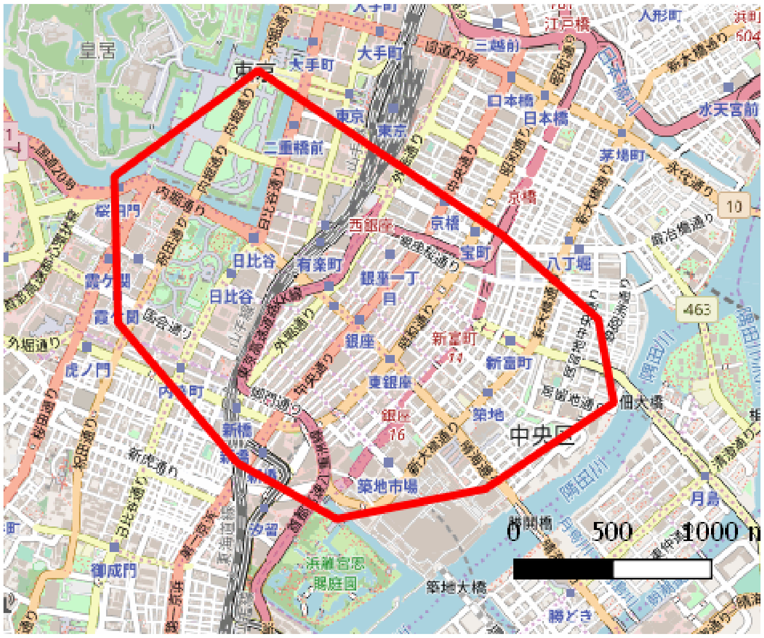

(a) Network exhibit

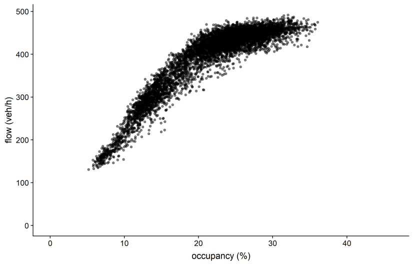

(b) Raw data

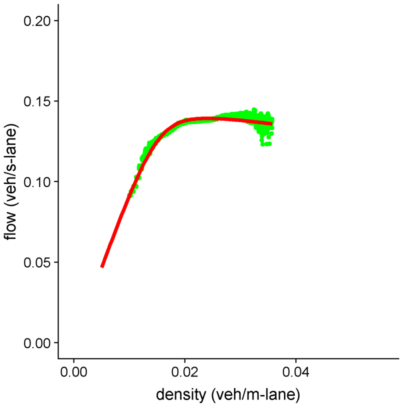

(c) Upper bound and fitted curve

Figure 87: Tokyo Region IX. (**a**) shows the network exhibit used for the MFD estimation and calculation of network topology variables. The map is oriented to the north and is obtained from OpenStreetMap. (**b**) presents the scatter plot of the raw, i.e., unfiltered, data of the network shown in **a**. This includes all malfunctioning detectors, oversampling of lanes, detectors on residential roads etc. (**c**) shows the upper-bound of the estimated MFD and the fitted curve based on the log-exp function by Ambühl et al. (2018). Flow and density are per lane. Occupancy is in the interval 0 to 1 and means the fraction of time in the observation interval a detector is covered by vehicles, i.e., it approximates density.

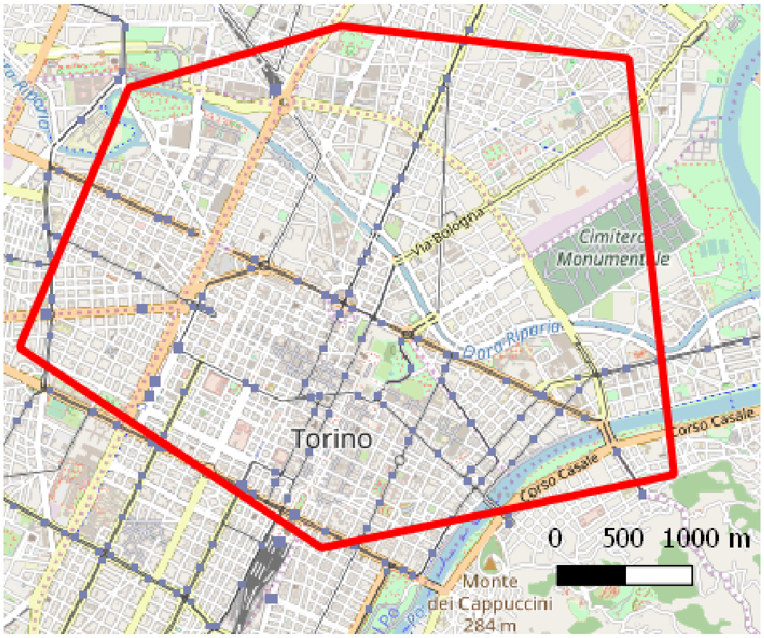

(a) Network exhibit

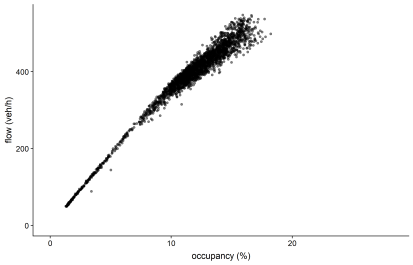

(b) Raw data

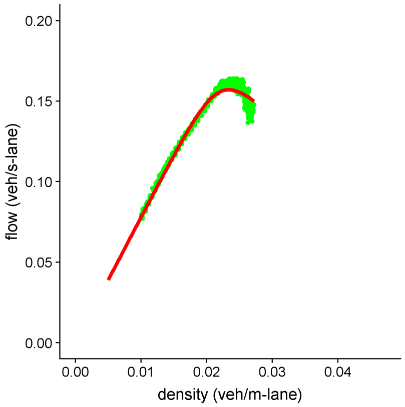

(c) Upper bound and fitted curve

Figure 88: Torino Region I. (**a**) shows the network exhibit used for the MFD estimation and calculation of network topology variables. The map is oriented to the north and is obtained from OpenStreetMap. (**b**) presents the scatter plot of the raw, i.e., unfiltered, data of the network shown in **a**. This includes all malfunctioning detectors, oversampling of lanes, detectors on residential roads etc. (**c**) shows the upper-bound of the estimated MFD and the fitted curve based on the log-exp function by Ambühl et al. (2018). Flow and density are per lane. Occupancy is in the interval 0 to 1 and means the fraction of time in the observation interval a detector is covered by vehicles, i.e., it approximates density.

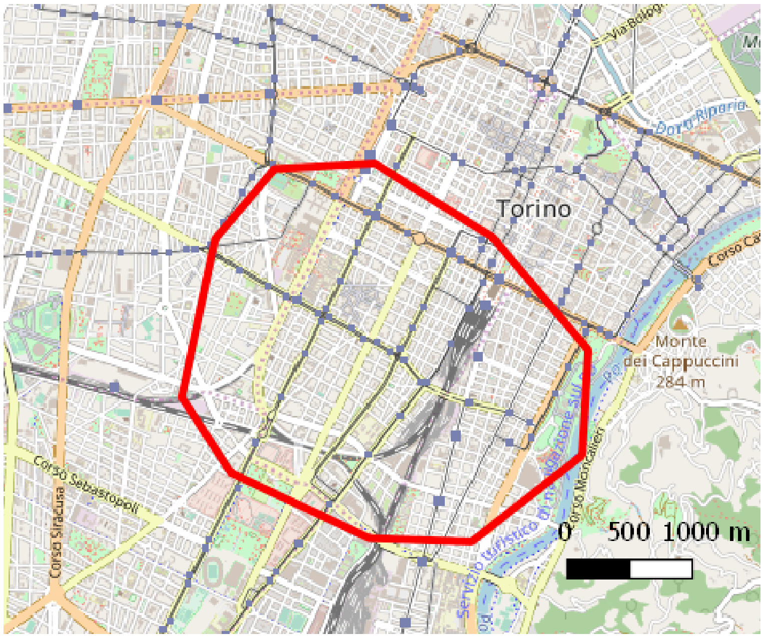

(a) Network exhibit

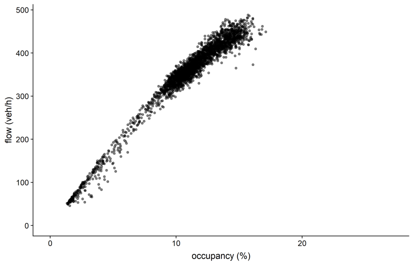

(b) Raw data

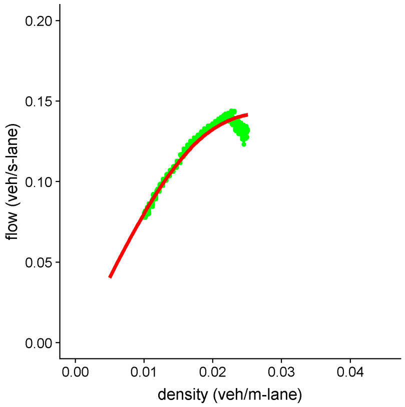

(c) Upper bound and fitted curve

Figure 89: Torino Region II. (**a**) shows the network exhibit used for the MFD estimation and calculation of network topology variables. The map is oriented to the north and is obtained from OpenStreetMap. (**b**) presents the scatter plot of the raw, i.e., unfiltered, data of the network shown in **a**. This includes all malfunctioning detectors, oversampling of lanes, detectors on residential roads etc. (**c**) shows the upper-bound of the estimated MFD and the fitted curve based on the log-exp function by Ambühl et al. (2018). Flow and density are per lane. Occupancy is in the interval 0 to 1 and means the fraction of time in the observation interval a detector is covered by vehicles, i.e., it approximates density.

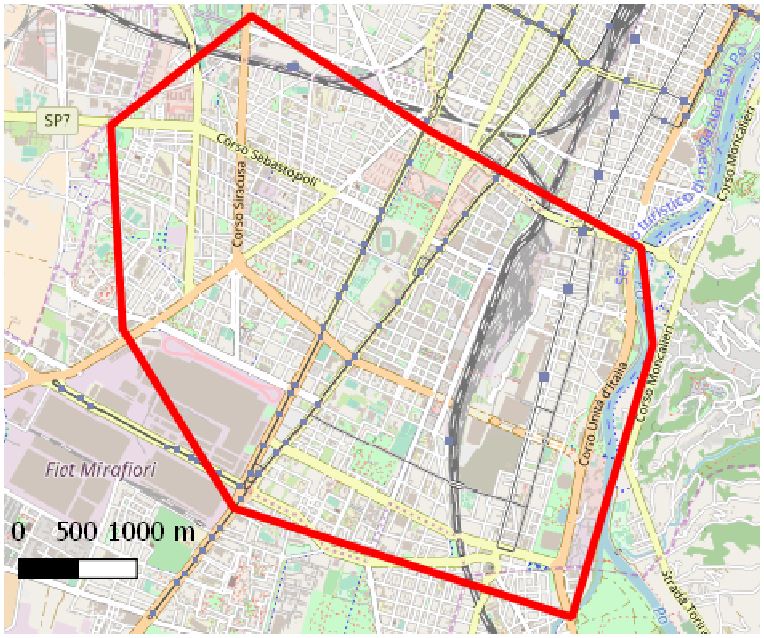

(a) Network exhibit

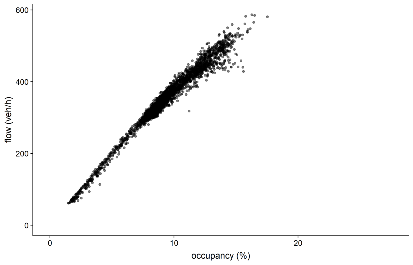

(b) Raw data

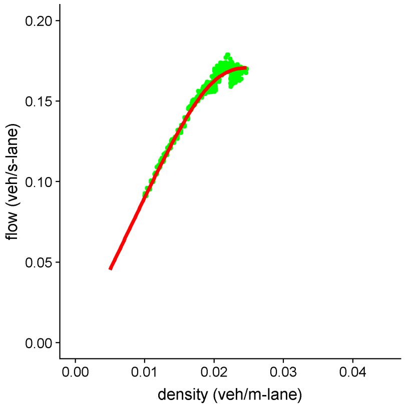

(c) Upper bound and fitted curve

Figure 90: Torino Region III. (**a**) shows the network exhibit used for the MFD estimation and calculation of network topology variables. The map is oriented to the north and is obtained from OpenStreetMap. (**b**) presents the scatter plot of the raw, i.e., unfiltered, data of the network shown in **a**. This includes all malfunctioning detectors, oversampling of lanes, detectors on residential roads etc. (**c**) shows the upper-bound of the estimated MFD and the fitted curve based on the log-exp function by Ambühl et al. (2018). Flow and density are per lane. Occupancy is in the interval 0 to 1 and means the fraction of time in the observation interval a detector is covered by vehicles, i.e., it approximates density.

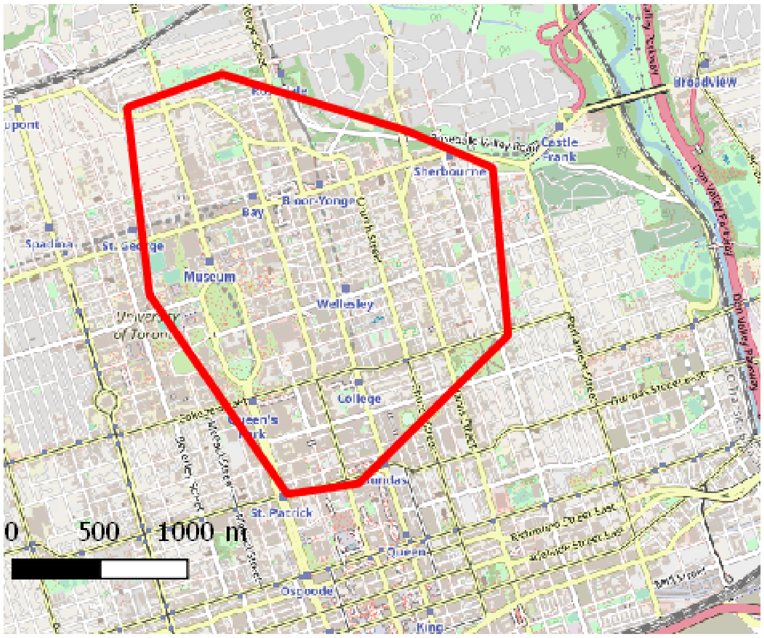

(a) Network exhibit

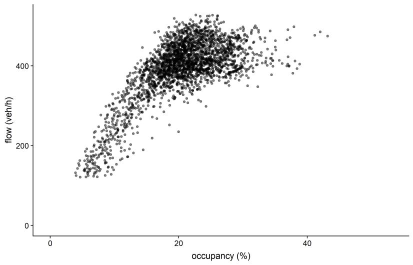

(b) Raw data

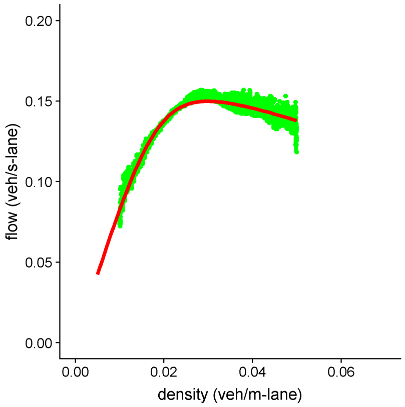

(c) Upper bound and fitted curve

Figure 91: Toronto Region I. (**a**) shows the network exhibit used for the MFD estimation and calculation of network topology variables. The map is oriented to the north and is obtained from OpenStreetMap. (**b**) presents the scatter plot of the raw, i.e., unfiltered, data of the network shown in **a**. This includes all malfunctioning detectors, oversampling of lanes, detectors on residential roads etc. (**c**) shows the upper-bound of the estimated MFD and the fitted curve based on the log-exp function by Ambühl et al. (2018). Flow and density are per lane. Occupancy is in the interval 0 to 1 and means the fraction of time in the observation interval a detector is covered by vehicles, i.e., it approximates density.

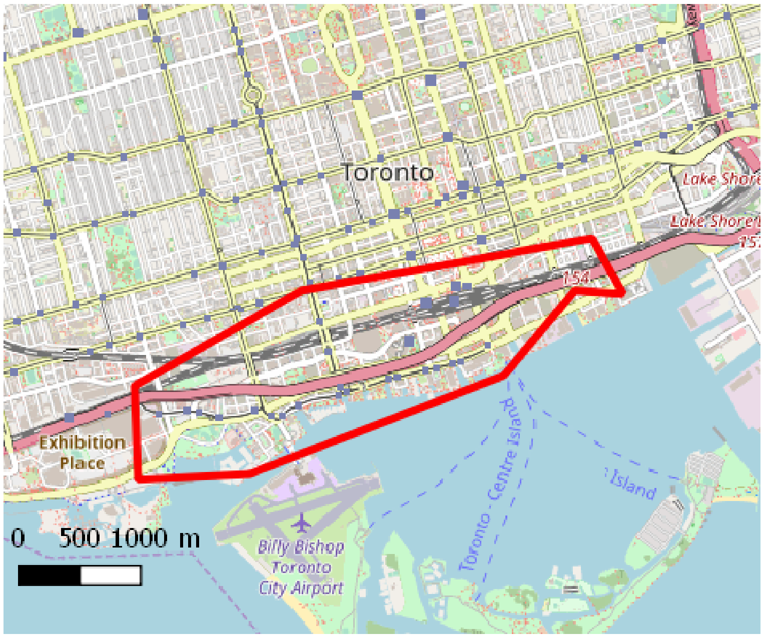

(a) Network exhibit

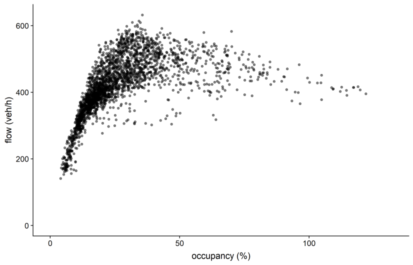

(b) Raw data

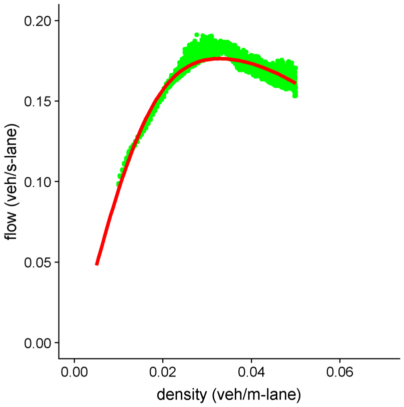

(c) Upper bound and fitted curve

Figure 92: Toronto Region II. (**a**) shows the network exhibit used for the MFD estimation and calculation of network topology variables. The map is oriented to the north and is obtained from OpenStreetMap. (**b**) presents the scatter plot of the raw, i.e., unfiltered, data of the network shown in **a**. This includes all malfunctioning detectors, oversampling of lanes, detectors on residential roads etc. (**c**) shows the upper-bound of the estimated MFD and the fitted curve based on the log-exp function by Ambühl et al. (2018). Flow and density are per lane. Occupancy is in the interval 0 to 1 and means the fraction of time in the observation interval a detector is covered by vehicles, i.e., it approximates density.

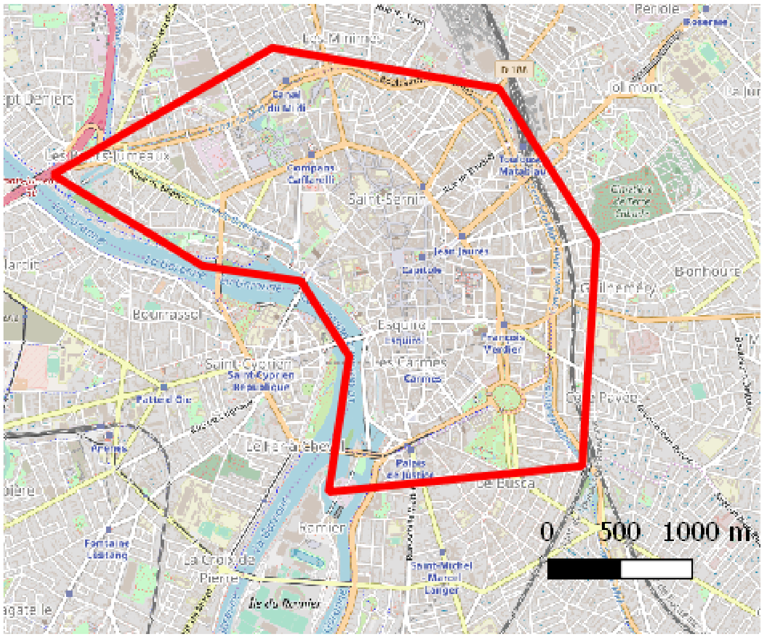

(a) Network exhibit

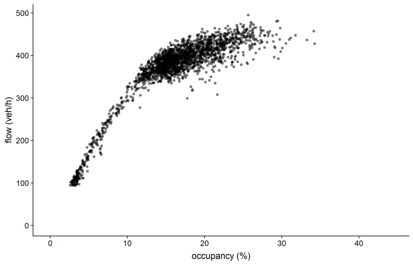

(b) Raw data

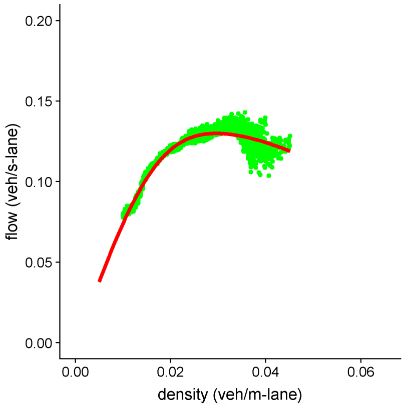

(c) Upper bound and fitted curve

Figure 93: Toulouse Region I. (**a**) shows the network exhibit used for the MFD estimation and calculation of network topology variables. The map is oriented to the north and is obtained from OpenStreetMap. (**b**) presents the scatter plot of the raw, i.e., unfiltered, data of the network shown in **a**. This includes all malfunctioning detectors, oversampling of lanes, detectors on residential roads etc. (**c**) shows the upper-bound of the estimated MFD and the fitted curve based on the log-exp function by Ambühl et al. (2018). Flow and density are per lane. Occupancy is in the interval 0 to 1 and means the fraction of time in the observation interval a detector is covered by vehicles, i.e., it approximates density.

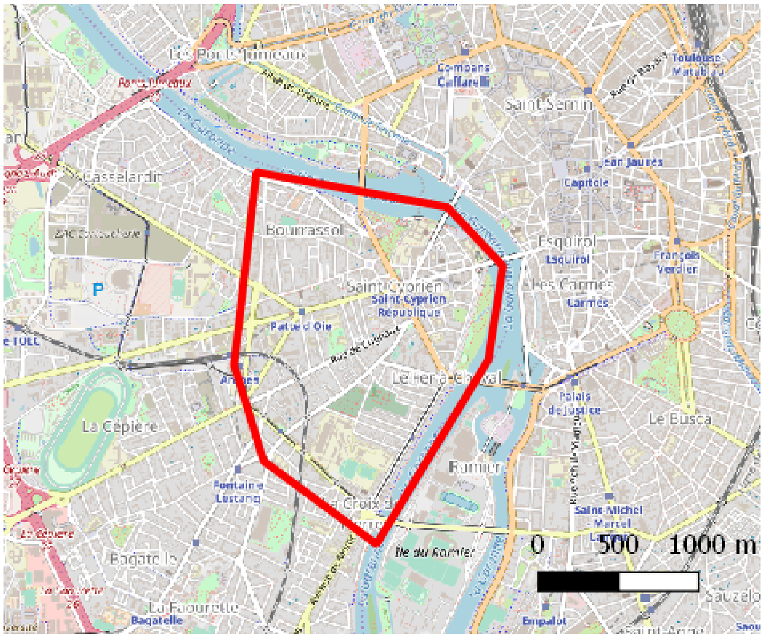

(a) Network exhibit

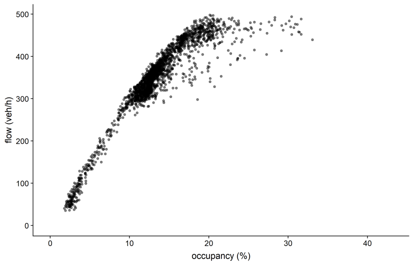

(b) Raw data

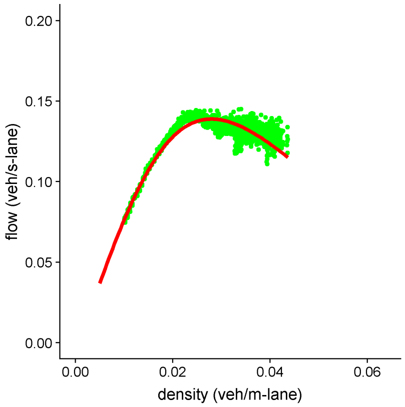

(c) Upper bound and fitted curve

Figure 94: Toulouse Region II. (**a**) shows the network exhibit used for the MFD estimation and calculation of network topology variables. The map is oriented to the north and is obtained from OpenStreetMap. (**b**) presents the scatter plot of the raw, i.e., unfiltered, data of the network shown in **a**. This includes all malfunctioning detectors, oversampling of lanes, detectors on residential roads etc. (**c**) shows the upper-bound of the estimated MFD and the fitted curve based on the log-exp function by Ambühl et al. (2018). Flow and density are per lane. Occupancy is in the interval 0 to 1 and means the fraction of time in the observation interval a detector is covered by vehicles, i.e., it approximates density.

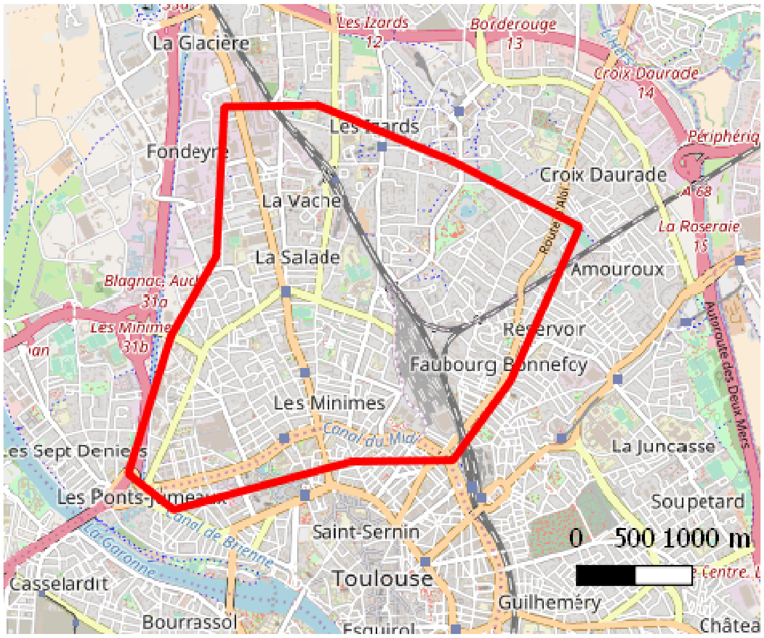

(a) Network exhibit

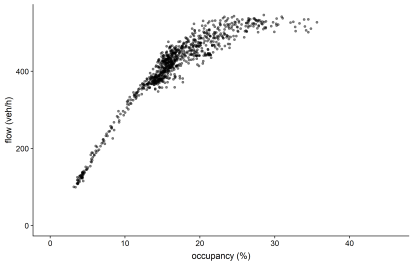

(b) Raw data

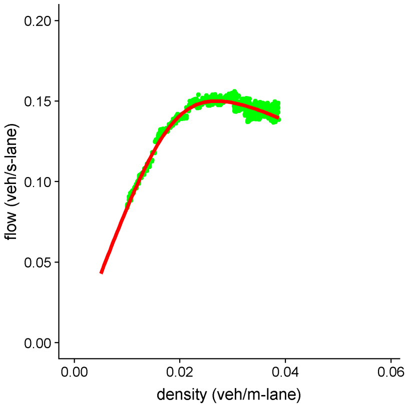

(c) Upper bound and fitted curve

Figure 95: Toulouse Region III. (**a**) shows the network exhibit used for the MFD estimation and calculation of network topology variables. The map is oriented to the north and is obtained from OpenStreetMap. (**b**) presents the scatter plot of the raw, i.e., unfiltered, data of the network shown in **a**. This includes all malfunctioning detectors, oversampling of lanes, detectors on residential roads etc. (**c**) shows the upper-bound of the estimated MFD and the fitted curve based on the log-exp function by Ambühl et al. (2018). Flow and density are per lane. Occupancy is in the interval 0 to 1 and means the fraction of time in the observation interval a detector is covered by vehicles, i.e., it approximates density.

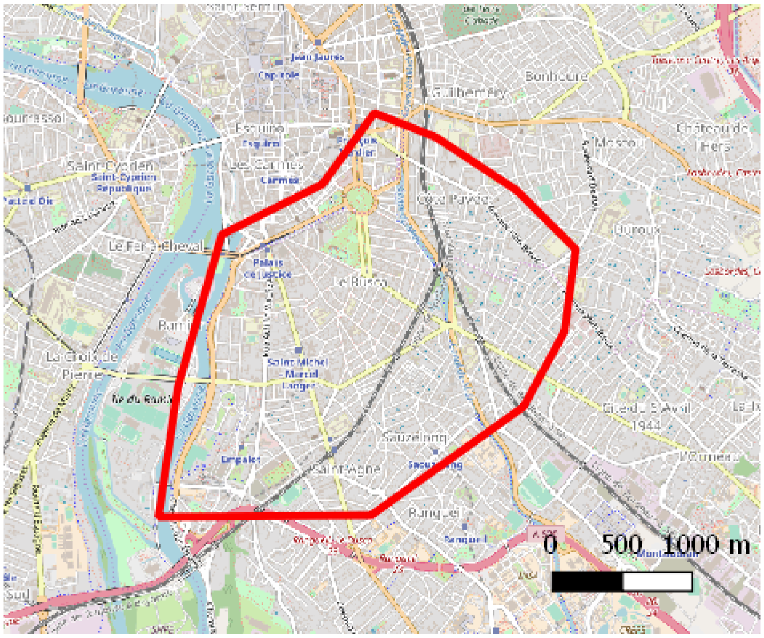

(a) Network exhibit

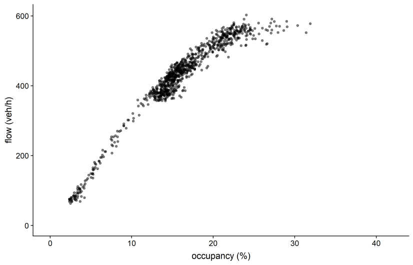

(b) Raw data

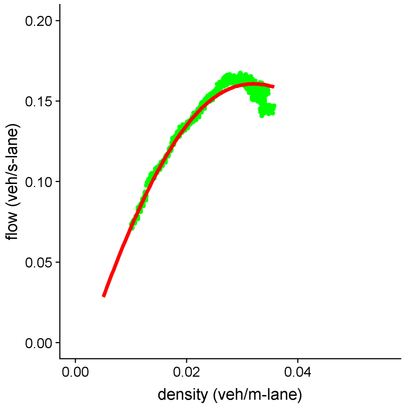

(c) Upper bound and fitted curve

Figure 96: Toulouse Region IV. (**a**) shows the network exhibit used for the MFD estimation and calculation of network topology variables. The map is oriented to the north and is obtained from OpenStreetMap. (**b**) presents the scatter plot of the raw, i.e., unfiltered, data of the network shown in **a**. This includes all malfunctioning detectors, oversampling of lanes, detectors on residential roads etc. (**c**) shows the upper-bound of the estimated MFD and the fitted curve based on the log-exp function by Ambühl et al. (2018). Flow and density are per lane. Occupancy is in the interval 0 to 1 and means the fraction of time in the observation interval a detector is covered by vehicles, i.e., it approximates density.

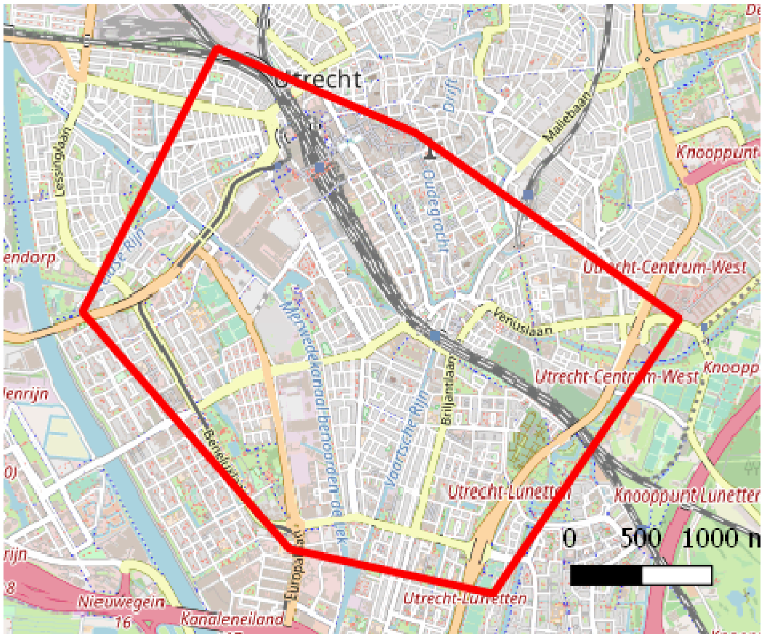

(a) Network exhibit

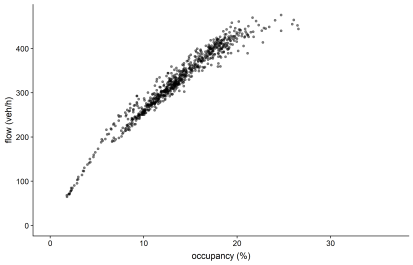

(b) Raw data

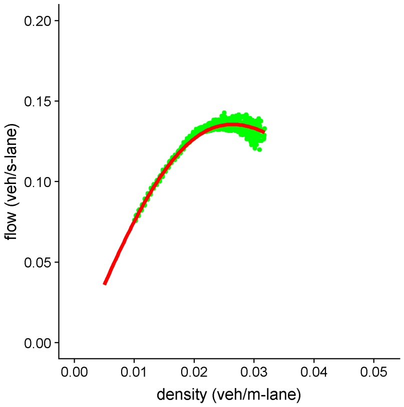

(c) Upper bound and fitted curve

Figure 97: Utrecht Region I. (**a**) shows the network exhibit used for the MFD estimation and calculation of network topology variables. The map is oriented to the north and is obtained from OpenStreetMap. (**b**) presents the scatter plot of the raw, i.e., unfiltered, data of the network shown in **a**. This includes all malfunctioning detectors, oversampling of lanes, detectors on residential roads etc. (**c**) shows the upper-bound of the estimated MFD and the fitted curve based on the log-exp function by Ambühl et al. (2018). Flow and density are per lane. Occupancy is in the interval 0 to 1 and means the fraction of time in the observation interval a detector is covered by vehicles, i.e., it approximates density.

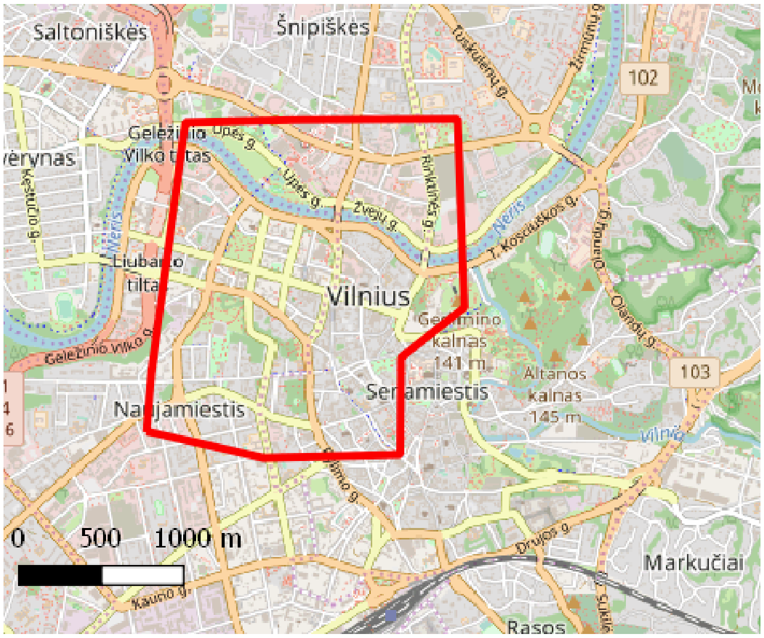

(a) Network exhibit

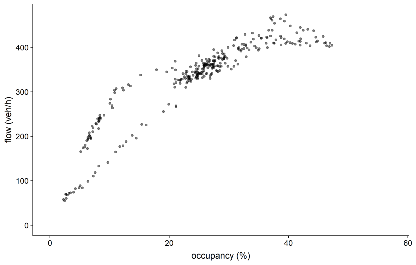

(b) Raw data

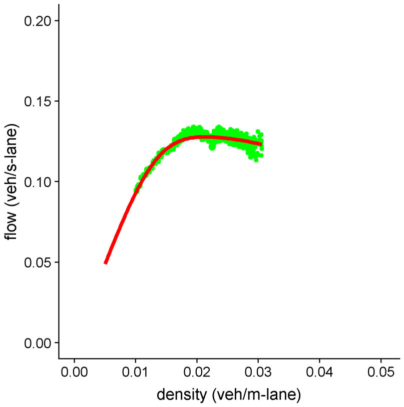

(c) Upper bound and fitted curve

Figure 98: Vilnius Region I. (**a**) shows the network exhibit used for the MFD estimation and calculation of network topology variables. The map is oriented to the north and is obtained from OpenStreetMap. (**b**) presents the scatter plot of the raw, i.e., unfiltered, data of the network shown in **a**. This includes all malfunctioning detectors, oversampling of lanes, detectors on residential roads etc. (**c**) shows the upper-bound of the estimated MFD and the fitted curve based on the log-exp function by Ambühl et al. (2018). Flow and density are per lane. Occupancy is in the interval 0 to 1 and means the fraction of time in the observation interval a detector is covered by vehicles, i.e., it approximates density.

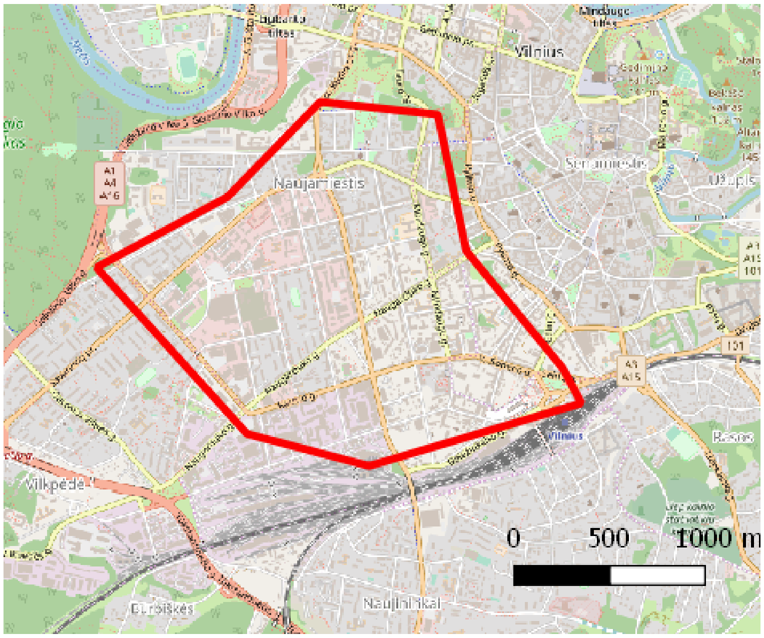

(a) Network exhibit

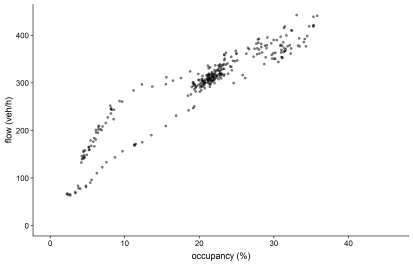

(b) Raw data

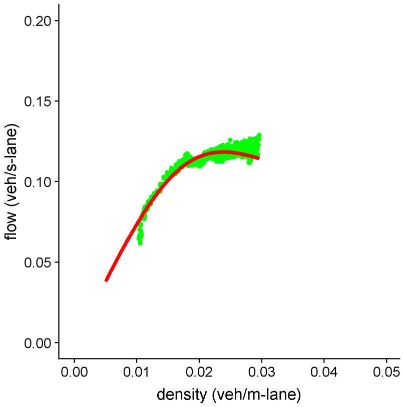

(c) Upper bound and fitted curve

Figure 99: Vilnius Region II. (**a**) shows the network exhibit used for the MFD estimation and calculation of network topology variables. The map is oriented to the north and is obtained from OpenStreetMap. (**b**) presents the scatter plot of the raw, i.e., unfiltered, data of the network shown in **a**. This includes all malfunctioning detectors, oversampling of lanes, detectors on residential roads etc. (**c**) shows the upper-bound of the estimated MFD and the fitted curve based on the log-exp function by Ambühl et al. (2018). Flow and density are per lane. Occupancy is in the interval 0 to 1 and means the fraction of time in the observation interval a detector is covered by vehicles, i.e., it approximates density.

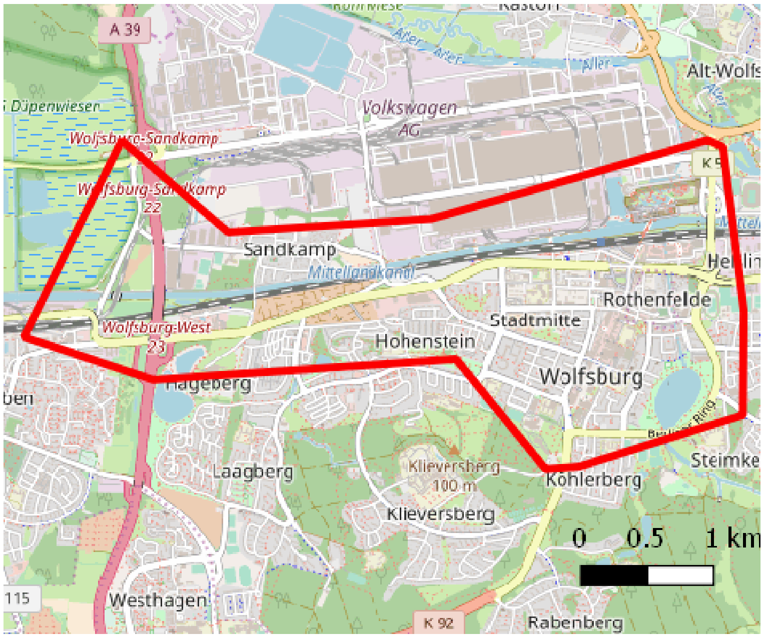

(a) Network exhibit

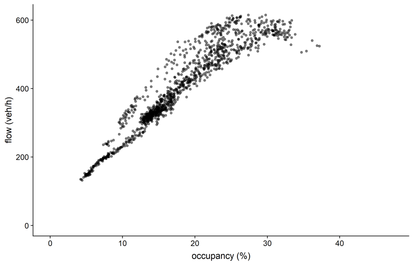

(b) Raw data

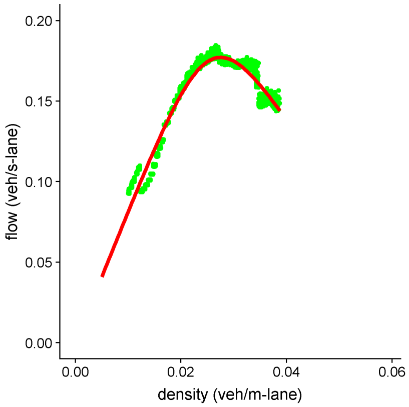

(c) Upper bound and fitted curve

Figure 100: Wolfsburg Region I. (**a**) shows the network exhibit used for the MFD estimation and calculation of network topology variables. The map is oriented to the north and is obtained from OpenStreetMap. (**b**) presents the scatter plot of the raw, i.e., unfiltered, data of the network shown in **a**. This includes all malfunctioning detectors, oversampling of lanes, detectors on residential roads etc. (**c**) shows the upper-bound of the estimated MFD and the fitted curve based on the log-exp function by Ambühl et al. (2018). Flow and density are per lane. Occupancy is in the interval 0 to 1 and means the fraction of time in the observation interval a detector is covered by vehicles, i.e., it approximates density.

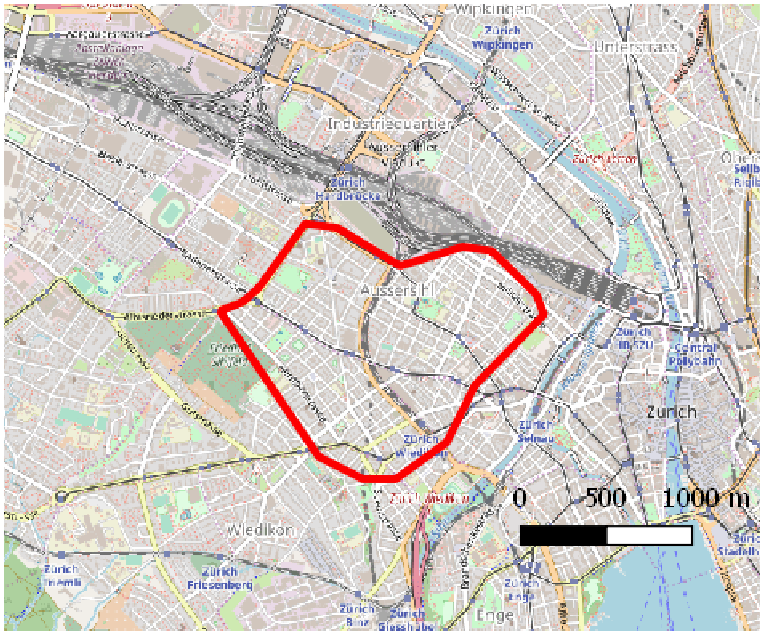

(a) Network exhibit

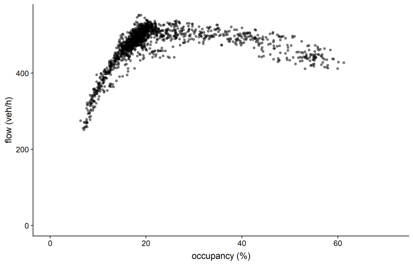

(b) Raw data

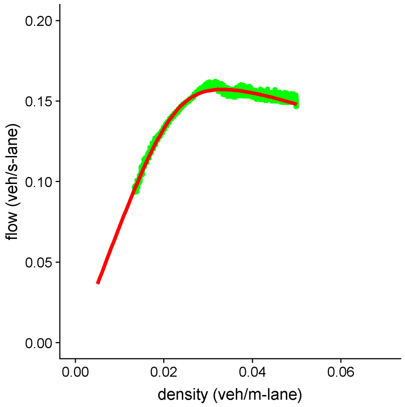

(c) Upper bound and fitted curve

Figure 101: Zurich Region I. (**a**) shows the network exhibit used for the MFD estimation and calculation of network topology variables. The map is oriented to the north and is obtained from OpenStreetMap. (**b**) presents the scatter plot of the raw, i.e., unfiltered, data of the network shown in **a**. This includes all malfunctioning detectors, oversampling of lanes, detectors on residential roads etc. (**c**) shows the upper-bound of the estimated MFD and the fitted curve based on the log-exp function by Ambühl et al. (2018). Flow and density are per lane. Occupancy is in the interval 0 to 1 and means the fraction of time in the observation interval a detector is covered by vehicles, i.e., it approximates density.

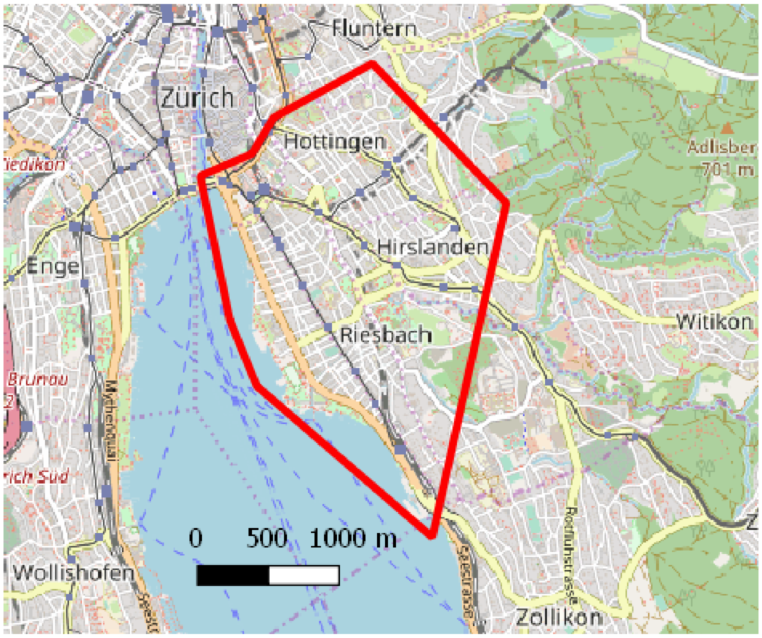

(a) Network exhibit

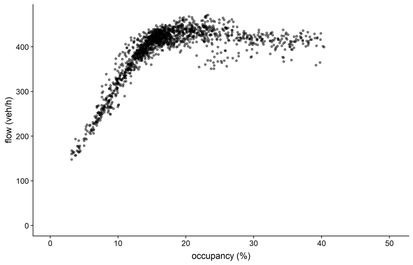

(b) Raw data

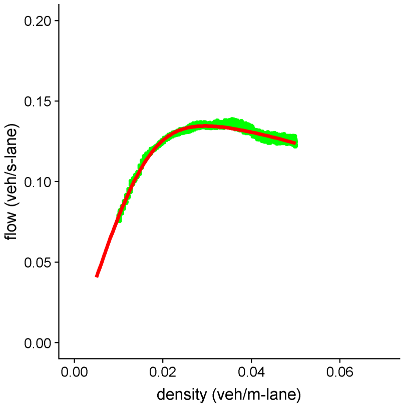

(c) Upper bound and fitted curve

Figure 102: Zurich Region II. (**a**) shows the network exhibit used for the MFD estimation and calculation of network topology variables. The map is oriented to the north and is obtained from OpenStreetMap. (**b**) presents the scatter plot of the raw, i.e., unfiltered, data of the network shown in **a**. This includes all malfunctioning detectors, oversampling of lanes, detectors on residential roads etc. (**c**) shows the upper-bound of the estimated MFD and the fitted curve based on the log-exp function by Ambühl et al. (2018). Flow and density are per lane. Occupancy is in the interval 0 to 1 and means the fraction of time in the observation interval a detector is covered by vehicles, i.e., it approximates density.

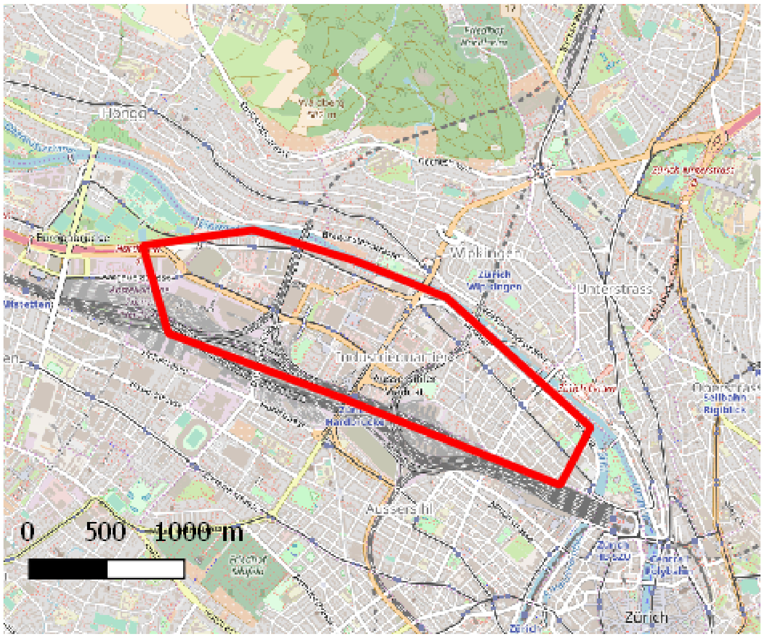

(a) Network exhibit

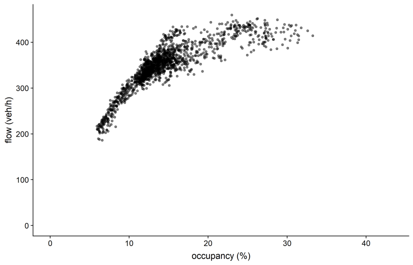

(b) Raw data

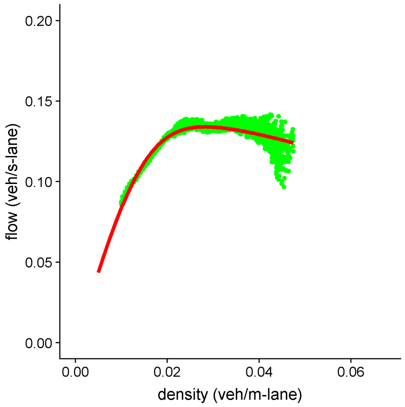

(c) Upper bound and fitted curve

Figure 103: Zurich Region III. (**a**) shows the network exhibit used for the MFD estimation and calculation of network topology variables. The map is oriented to the north and is obtained from OpenStreetMap. (**b**) presents the scatter plot of the raw, i.e., unfiltered, data of the network shown in **a**. This includes all malfunctioning detectors, oversampling of lanes, detectors on residential roads etc. (**c**) shows the upper-bound of the estimated MFD and the fitted curve based on the log-exp function by Ambühl et al. (2018). Flow and density are per lane. Occupancy is in the interval 0 to 1 and means the fraction of time in the observation interval a detector is covered by vehicles, i.e., it approximates density.

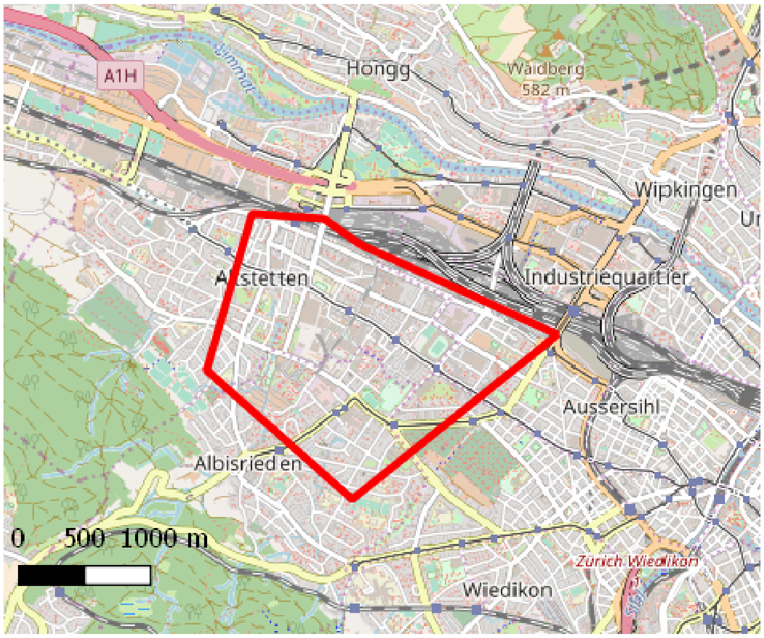

(a) Network exhibit

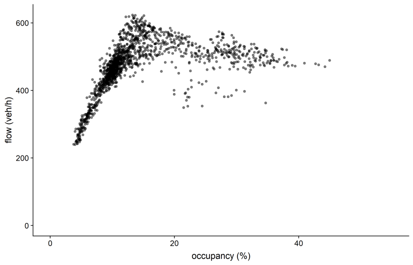

(b) Raw data

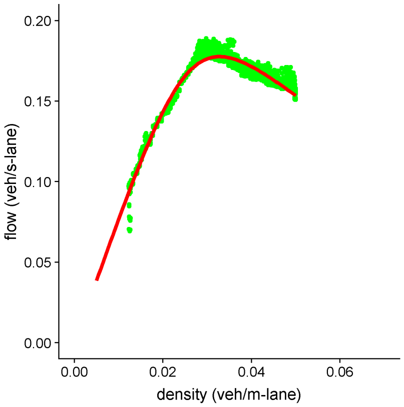

(c) Upper bound and fitted curve

Figure 104: Zurich Region IV. (**a**) shows the network exhibit used for the MFD estimation and calculation of network topology variables. The map is oriented to the north and is obtained from OpenStreetMap. (**b**) presents the scatter plot of the raw, i.e., unfiltered, data of the network shown in **a**. This includes all malfunctioning detectors, oversampling of lanes, detectors on residential roads etc. (**c**) shows the upper-bound of the estimated MFD and the fitted curve based on the log-exp function by Ambühl et al. (2018). Flow and density are per lane. Occupancy is in the interval 0 to 1 and means the fraction of time in the observation interval a detector is covered by vehicles, i.e., it approximates density.

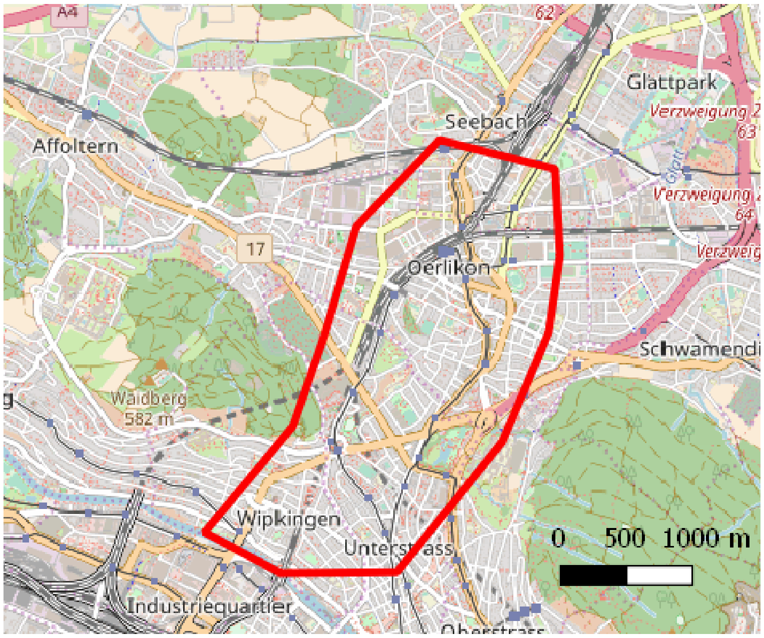

(a) Network exhibit

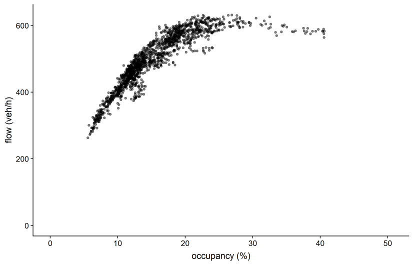

(b) Raw data

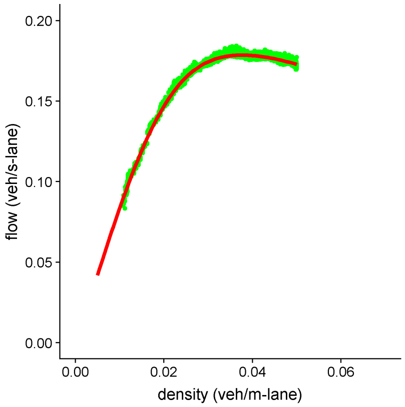

(c) Upper bound and fitted curve

Figure 105: Zurich Region V. (**a**) shows the network exhibit used for the MFD estimation and calculation of network topology variables. The map is oriented to the north and is obtained from OpenStreetMap. (**b**) presents the scatter plot of the raw, i.e., unfiltered, data of the network shown in **a**. This includes all malfunctioning detectors, oversampling of lanes, detectors on residential roads etc. (**c**) shows the upper-bound of the estimated MFD and the fitted curve based on the log-exp function by Ambühl et al. (2018). Flow and density are per lane. Occupancy is in the interval 0 to 1 and means the fraction of time in the observation interval a detector is covered by vehicles, i.e., it approximates density.

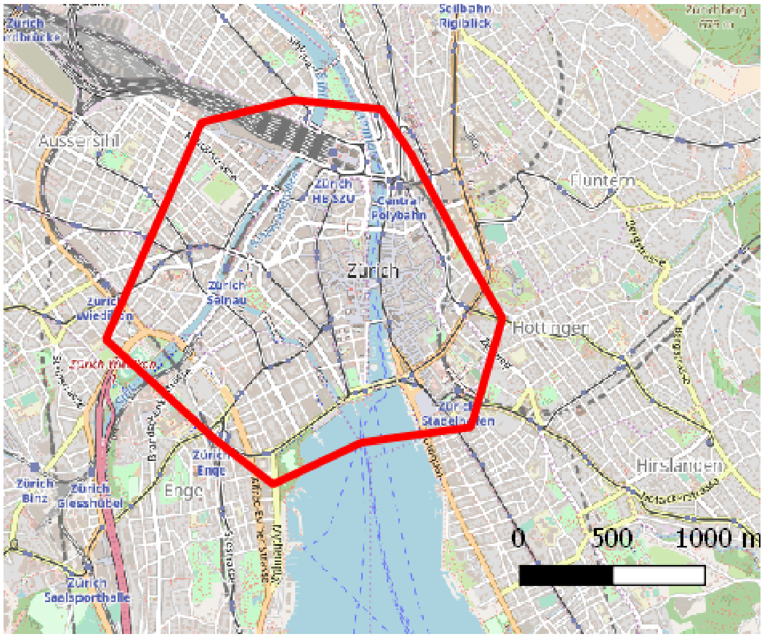

(a) Network exhibit

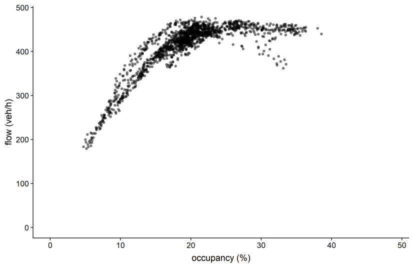

(b) Raw data

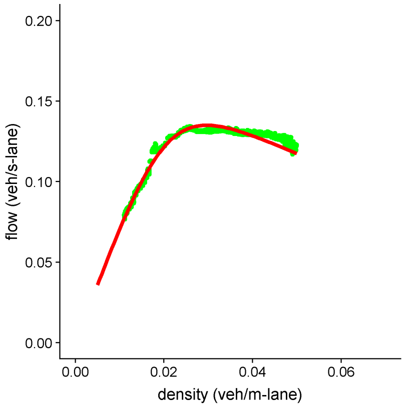

(c) Upper bound and fitted curve

Figure 106: Zurich Region VI. (**a**) shows the network exhibit used for the MFD estimation and calculation of network topology variables. The map is oriented to the north and is obtained from OpenStreetMap. (**b**) presents the scatter plot of the raw, i.e., unfiltered, data of the network shown in **a**. This includes all malfunctioning detectors, oversampling of lanes, detectors on residential roads etc. (**c**) shows the upper-bound of the estimated MFD and the fitted curve based on the log-exp function by Ambühl et al. (2018). Flow and density are per lane. Occupancy is in the interval 0 to 1 and means the fraction of time in the observation interval a detector is covered by vehicles, i.e., it approximates density.

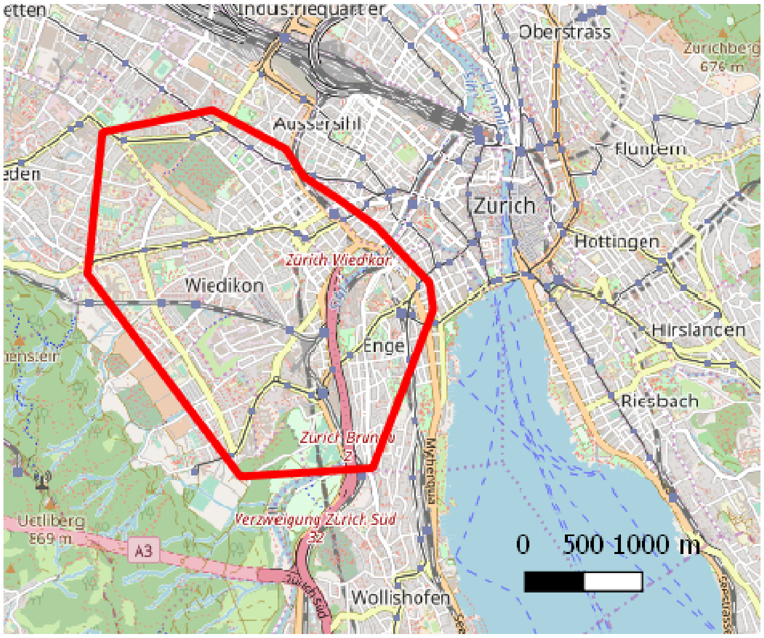

(a) Network exhibit

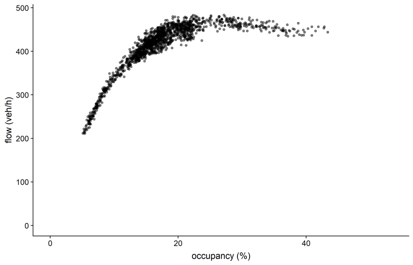

(b) Raw data

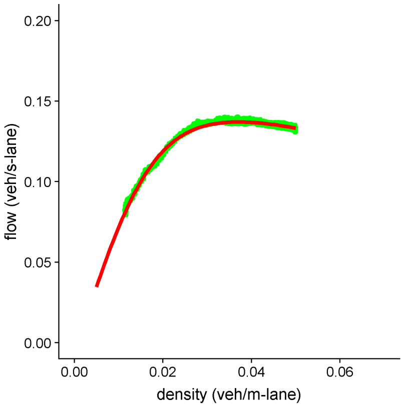

(c) Upper bound and fitted curve

Figure 107: Zurich Region VII. (**a**) shows the network exhibit used for the MFD estimation and calculation of network topology variables. The map is oriented to the north and is obtained from OpenStreetMap. (**b**) presents the scatter plot of the raw, i.e., unfiltered, data of the network shown in **a**. This includes all malfunctioning detectors, oversampling of lanes, detectors on residential roads etc. (**c**) shows the upper-bound of the estimated MFD and the fitted curve based on the log-exp function by Ambühl et al. (2018). Flow and density are per lane. Occupancy is in the interval 0 to 1 and means the fraction of time in the observation interval a detector is covered by vehicles, i.e., it approximates density.
